# Facilitating the use of the target product profile in academic research: a systematic review

**DOI:** 10.1186/s12967-024-05476-1

**Published:** 2024-07-29

**Authors:** Aliaa Ibnidris, Nektarios Liaskos, Ece Eldem, Angus Gunn, Johannes Streffer, Michael Gold, Mike Rea, Stefan Teipel, Alejandra Gardiol, Marina Boccardi

**Affiliations:** 1 German Center for Neurodegenerative Diseases (DZNE), Rostock-Greifswald site, Gehlsheimer Str. 20, 18147 Rostock, Germany; 2 Neuroscience Institute, Department of Psychiatry and Mental Health, University of Cape Town, Cape Town, South Africa; 3 European Infrastructure for Translational Medicine (EATRIS), Amsterdam, The Netherlands; 4 UCB, Brussels, Belgium; 5 Reference Center for Biological Markers of Dementia (BIODEM), Department of Biomedical Sciences, University of Antwerp, Antwerp, Belgium; 6 AriLex Life Sciences LLC, 780 Elysian Way, Deerfield, IL 60015 USA; 7 IDEA Pharma, London, UK; 8 Department of Psychosomatic Medicine and Psychotherapy, University of Medicine Rostock, Rostock, Germany; 9 Queen Mary University of London, London, UK

**Keywords:** Target product profile, TPP, Quality by design, Translational research, Translational methods, Methodology

## Abstract

**Background:**

The Target Product Profile (TPP) is a tool used in industry to guide development strategies by addressing user needs and fostering effective communication among stakeholders. However, they are not frequently used in academic research, where they may be equally useful. This systematic review aims to extract the features of accessible TPPs, to identify commonalities and facilitate their integration in academic research methodology.

**Methods:**

We searched peer-reviewed papers published in English developing TPPs for different products and health conditions in four biomedical databases. Interrater agreement, computed on random abstract and paper sets (Cohen’s Kappa; percentage agreement with zero tolerance) was > 0.91. We interviewed experts from industry contexts to gain insight on the process of TPP development, and extracted general and specific features on TPP use and structure.

**Results:**

138 papers were eligible for data extraction. Of them, 92% (*n* = 128) developed a new TPP, with 41.3% (*n* = 57) focusing on therapeutics. The addressed disease categories were diverse; the largest (47.1%, *n* = 65) was infectious diseases. Only one TPP was identified for several fields, including global priorities like dementia. Our analyses found that 56.5% of papers (*n* = 78) was authored by academics, and 57.8% of TPPs (*n* = 80) featured one threshold level of product performance. The number of TPP features varied widely across and within product types (*n* = 3–44). Common features included purpose/context of use, shelf life for drug stability and validation aspects. Most papers did not describe the methods used to develop the TPP. We identified aspects to be taken into account to build and report TPPs, as a starting point for more focused initiatives guiding use by academics.

**Discussion:**

TPPs are used in academic research mostly for infectious diseases and have heterogeneous features. Our extraction of key features and common structures helps to understand the tool and widen its use in academia. This is of particular relevance for areas of notable unmet needs, like dementia. Collaboration between stakeholders is key for innovation. Tools to streamline communication such as TPPs would support the development of products and services in academia as well as industry.

**Supplementary Information:**

The online version contains supplementary material available at 10.1186/s12967-024-05476-1.

## Introduction

A Target Product Profile (TPP) is a strategic document outlining the desired characteristics of a planned product, procedure or service intended for a particular disease or use case. Its goal is to guide in addressing users’ needs, facilitating stakeholders’ communication, and making best use of resources to develop a successful product. TPPs encompass context of use features, such as the target disease and populations, and specific desired attributes of the product, procedure or service under development [1–3]. TPPs are widely used in industry as a planning tool to guide product development and ensure that relevant product features be aligned among stakeholders. They are therefore treated confidentially, containing sensitive information about a company’s assets, product development plans and strategies (See Table S1 for a concrete example of TPP).

Although not common practice yet, TPPs may be useful in academia as well. Like industry, academics also develop therapeutics and diagnostics. However, academic research is often slower in adopting tools for systematic development, with consequent lower efficiency of translational research [4]. For example, the field of neurodegenerative disorders is validating biomarkers for Alzheimer’s disease since 2009 [5, 6], but only in 2017 did it import a systematic validation framework [7], first published in 2001 for oncology research [8] and similar to others used well before for imaging [9] and other biomarkers [10]. Adopting good practice procedures and tools commonly used in industry settings may reduce waste of efforts and costs, and increase the efficiency of academic translational research as well. Noteworthy, the World Health Organization (WHO) recommends the use of TPPs to facilitate the communication with research project funders to align funding strategies with prioritised unmet public healthcare needs. This is now urgent for the dementia field, to strive to meet the innovation goals set by the 2017 Global Action Plan, that, despite many efforts, still remain a distant ambition [11, 12]. Facilitating the incorporation of TPPs among the methods used in this field means therefore bringing a pivotal tool to upgrade translational methods and help boost its innovation efforts. The example of neurodegenerative disorders represents well several fields with a high prevalence of unmet needs.

The effort to help researchers to adopt TPPs was already initiated with a previous systematic review, summarizing the methods currently used to develop them and the sources used for the inclusion for each feature [13]. Focused on diagnostic tests, the authors found TPPs for infectious diseases only, and identified a 3-phase process for their development: (1) identifying the unmet need, (2) initial drafting of the TPP, and (3) building consensus among stakeholders. The outcome of that systematic review provided an insightful first glimpse on TPPs applications outside the pharma industry, as well as the rudiments to adapt the method to academic research. Our review aims to expand on these results and extract key structural features of TPPs across different therapeutic areas and product types, to gain a wider understanding of the tool’s structure and development and facilitate its use by academic researchers.

## Methods

This work stems from the IMI-2-funded project EPND (European Platform for Neurodegenerative Disorders – epnd.org). IMI (www.imi.europa.eu) is a collaborative initiative between the European Commission and EFPIA (European Federation of Pharmaceutical Industries and Associations), requiring the collaboration between partners from both academia and industry. EPND aims to build a platform making existing data and samples on neurodegenerative disorders FAIR (findable, accessible, interoperable and reusable). During the development of such platform, academic and industry project partners contributed to define a TPP supporting the development of the platform. As researchers, we leveraged this experience and know-how, to generate this review and try to import the tool for academic research.

We reported our methods following the Preferred Reporting Items for Systematic Reviews and Meta-Analyses (PRISMA) guidelines [14]. The PRISMA checklist is provided as supplementary material (see Supplementary material S2). The protocol for this systematic review was not registered in an open access platform. We performed the systematic review as detailed below. Then, based on the extracted data, we highlighted key features and the structure of retrieved TPPs, that we believe are useful to framework the tool.

### Information sources

#### Publication search:

We searched relevant publications in the PubMed, Medline, CINHAL, and Scopus biomedical databases in January 2023. Additionally, we hand-searched and screened primary publications in one identified systematic review. For grey literature, a Google search using the terms “target product profile” was used to identify publicly available TPPs (e.g., WHO TPPs, FIND, PATH). These TPPs were used to guide the conceptualization of the systematic review and the development of the search strategy. We also referred to the publicly available WHO TPPs [15] to understand the typical process and the methods commonly used to draft TPPs.

#### Interviews:

We asked availability to experts from the network of the last author, and to 3 regulators (Dutch, German and Norwegian), known for their activity within the European Medicine Agency. Regulators reported no familiarity with the tool (Netherlands and Germany) or did not reply (Norway). Four experts (AG, MG, MR, JS) from leading medical product development or consultancy companies accepted to be interviewed and provided general information on TPP use in the industry. The interviewed experts were senior professionals with extensive expertise on multiple aspects of research and development in pharma. Here we report their *initials*, affiliation and role at the time of the interview, and any additional expertise particularly relevant for this review: *AG*: UCB, Senior Global Director; external engagement with clinical and governmental communities for early clinical development; additional experience on regulatory intelligence through activities with CIRS (Center for Innovation and Regulatory Science). *MG*: AbbVie, Industry Co-Director. *MR*: IDEA Pharma, CEO. IDEA Pharma is a consultancy company advising on the path-to-market strategy. *JS*: AC Immune, Chief Medical Officer with broad responsibility for all clinical development functions. No confidential material was disclosed during the interviews. The collected qualitative information guided the review process and provided insights into how to process and contextualise the results.

### Search strategy

Using iterations of key terms such as “target product profile”, TPP, “quality by design” or QbD or QTTP we formulated a comprehensive search strategy applicable to all databases. The search strategy was: (“target product profile” OR TPP OR QTTP OR “quality by design” OR QdB).

### Eligibility criteria of included studies for final analysis

We included papers published in English that reported the development or revision of a target product profile (TPP) or described a pre-existing TPP used for the development of products across any health field. There were no restrictions on the publication date. Publications were eligible if they provided a TPP structure in the form of a table, figure or narrative description of the TPP features.

### Selection and data collection process

The first author screened all titles and abstracts to identify relevant publications using Rayyan, and then conducted a full-text screening of the included studies. A second reviewer (NL) independently screened 12% of the abstracts (*n* = 78) and 15% of the full-texts (*n* = 52). We calculated the inter-rater reliability between the two reviewers for both abstract and full-text screening with the Cohen’s Kappa and/or percentage agreement with zero tolerance using R Studio [16]. We used Zotero to manage and store references of included studies and created a data extraction tool using Excel.

### Data items

The target data items to be extracted included content, product type, disease category and the specific disease, performance thresholds for each TPP feature, authors’ affiliation, a full list of TPP features, the number and type of features and categories thereof, and the methods deployed to develop the TPP. We defined the data items as follows:

#### Content:

Describes whether a publication reports the development of a new TPP, revises a pre-defined TPP, or only describes an existing TPP.

#### Product type:

Indicates whether the target product consists of therapeutics, diagnostics, vaccine, medical device, or other (e.g., app, drug delivery system, etc.).

#### Disease category:

Describes whether the disease for which the TPP was reported was an infectious or non-infectious disease, as well as the specific disease.

#### Thresholds:

Reports whether each TPP feature includes one (target), two (minimal, ideal), or three (current practice, minimum acceptable, ideal) levels of possible performance or target quality achievement.

#### Affiliation of publishing authors:

Classifies the affiliation of publishing authors into academic (university, research institute, independent researchers), private (industry, consultancies, or other for-profit organisations), or public-private partnership (PPP) for collaborations between academic and private organisations.

#### List of TPP features:

The names of all features included in each TPP were stratified by product type.

#### Measure of variability:

Consists of the number of TPP features in each TPP.

#### Categories of TPP features:

Includes the number and type of categories by which TPP features were grouped. For example, the category named “Scope” may include TPP features such as target population, intended use, and the level of health care system implementation; similarly, “Operational characteristics” typically includes features such as cost of product, shelf life, power requirements, and training needs.

#### Framework:

Describes which framework was utilised to structure the TPP, e.g., the WHO or the FDA TPP guidance.

#### Criteria for the choice of TPP features:

Reports how an initial pool of TPP features and their target levels were chosen.

#### Consensus approach:

Categorises which kind of consensus procedure was followed to select the final set of TPP features. The approaches were divided into formal (e.g., Delphi process or survey) and informal (e.g., discussion in virtual or in-person meetings).

#### Experts:

Describes which experts were involved (e.g., clinicians, researchers, relevant product manufacturers, etc.).

#### Patient-public involvement:

Reports whether patient populations or the public were involved in the process of developing a TPP.

## Results

**Fig. 1:**
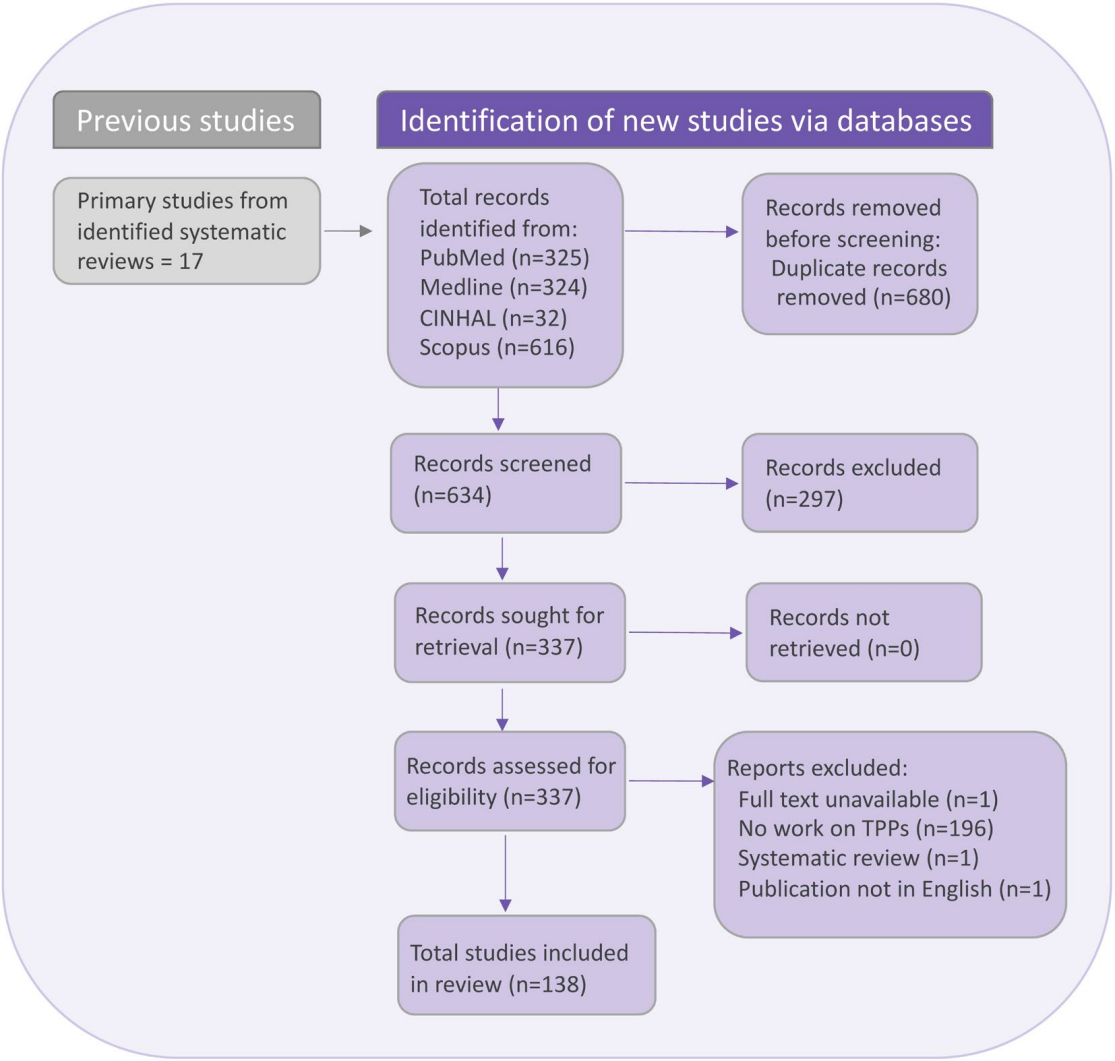
PRISMA flow chart of abstract and full text screening for including publications on target product profiles (TPP)

From the biomedical databases and hand searching, we identified 1314 records. After title and abstract screening, 337 met the eligibility criteria for full-text review, and 138 publications [3, 17–153] were eligible for data extraction (see Fig. 1 and Table S2 for the reasons and references of excluded studies). Raters had 100% agreement for abstract screening and Cohen’s Kappa = 0.912 (p-value < 0.001; 96.1% agreement with zero tolerance) for full-text screening.

**Fig. 2:**
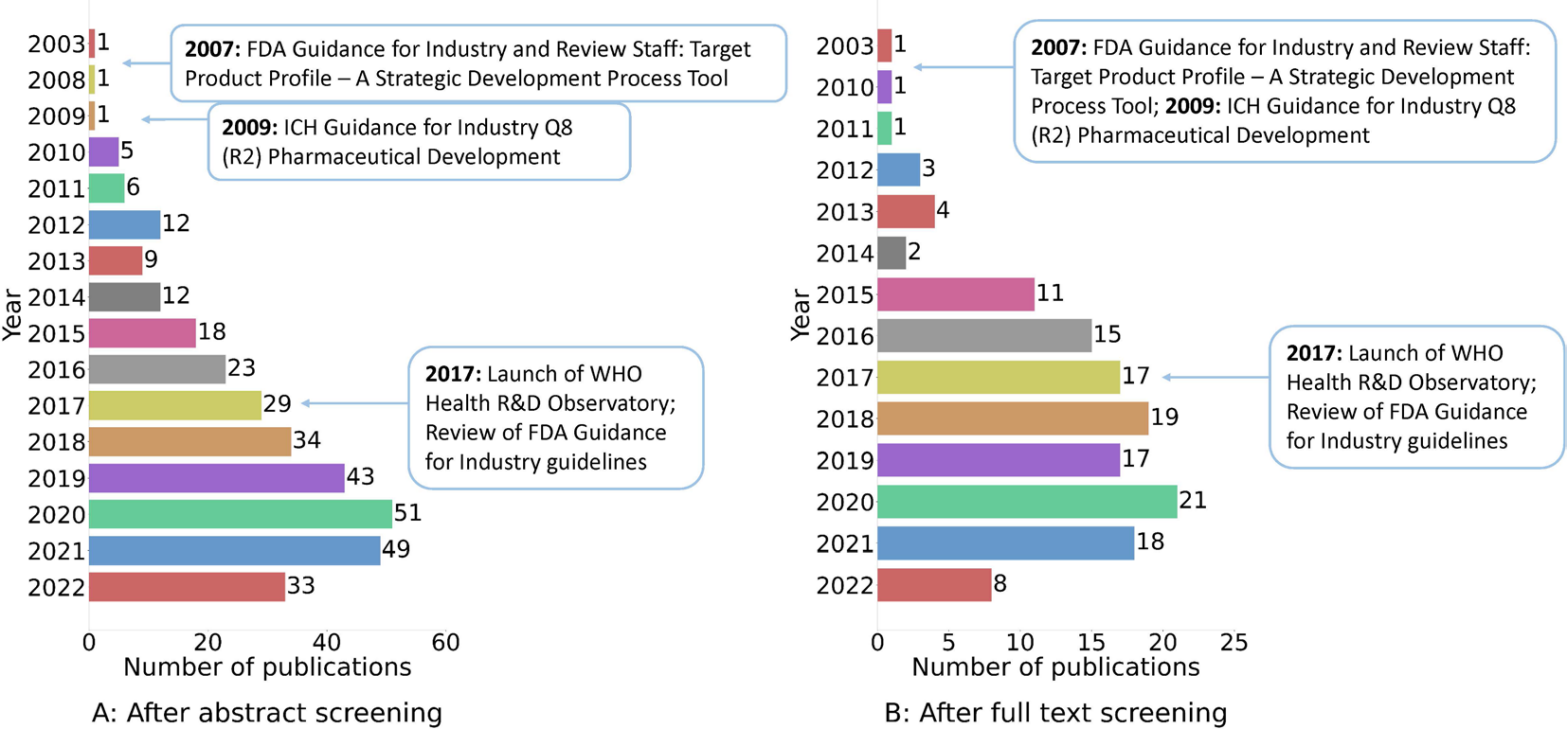
Distribution of publications on target product profiles (TPP) by year in relation to significant TPP-related events or publications by the WHO and the FDA. Panel **A** demonstrates the distribution of the 337 publications included after title and abstract screening. Panel **B** demonstrates the distribution of the 138 publications included after full text screening. *Abbreviations* TPP: Target product profile, FDA: Food and Drug Administration, WHO: World Health Organisation, R & D: Research and Development

Reports of TPPs steadily increased following the publications of the FDA [1] and ICH Q8 R2 [154] guidelines (Fig. 2), most contributions being in 2020. Of the 138 papers included in our review, 92% developed a new TPP, 4% revised a predefined TPP, and 4% described an existing TPP, with no overlap among these groups. The ICH Q8 R2 [154] was the most widely used framework (54%), followed by the FIND [155] (26%), the WHO [15] (15.4%) and the FDA guidance [1] (7.7%) (Table 1, upper panel). Combinations of frameworks (e.g., WHO and FIND) were also used.

**Fig. 3:**
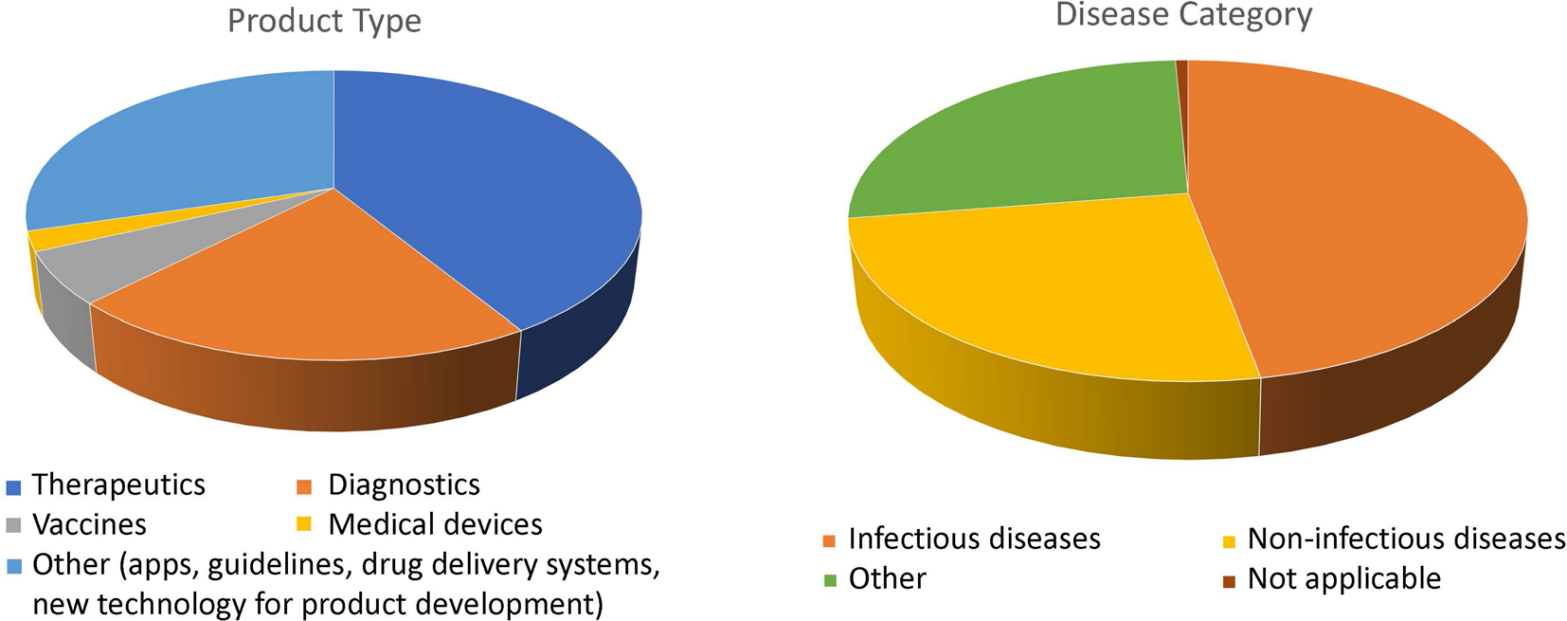
Proportion of product types (left panel) and disease categories (right panel) represented in the TPPs retrieved by our review

**Fig. 4:**
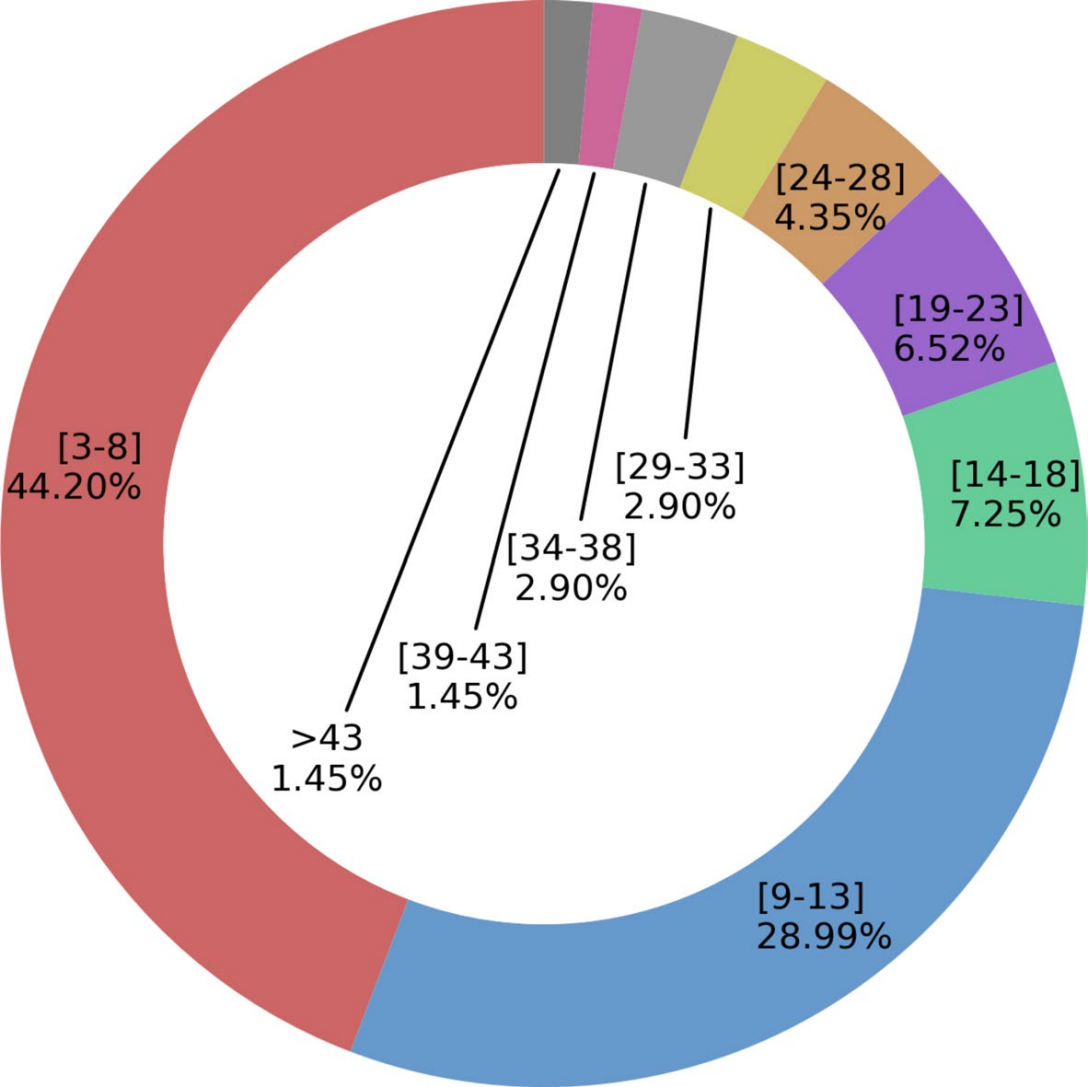
Proportion of the identified TPPs having low to high number of features (N of features in square brackets) across all product types. The pattern is replicated within specific product categories (see Figures S1-S3), with the exception of TPPs for diagnostics (Figure S1)

Product types included mostly therapeutics (41.3%) and diagnostics (21%), and a variety of products, like apps, new technology for product development, drug delivery systems, or clinical practice guidelines (“other products”: 29.7%; Fig. 3, Table S3). TPPs were used for products targeting infectious (47.1%) and non-infectious (25.4%) diseases, and unspecified disease categories, e.g., skin diseases possibly due to either infectious or non-infectious causes (26.8%; Fig. 3, Table S4). Among non-infectious diseases, one TPP was for a drug for Alzheimer’s disease [57], and one for a drug delivery system for an unspecified disease category [118]. The number of included features (range 3–44) was mostly between 3 and 8 (44%), across product types (Fig. 4; Figures S1-3). These included target population, indication, storage conditions and shelf life (Table 2; Table S5). Among TPPs reporting performance thresholds (89.2%), 57.9% set one target threshold, 28.9% set two (“minimum acceptable” and “ideal”), and 2.2% three (“current practice”, “minimum acceptable”, and “ideal”; Table S3). In the 15% of publications grouping TPP features into categories, “Scope” was the most frequent (90%; Tables S6-S7); others included “Operational”, “Performance”, and “Test characteristics” (complete list in Table S6). Criteria to define features and target levels were clarified in 17% of papers (Table 1, lower panel). Methods to agree on the TPP features were reported in 22% of the papers: these included formal consensus (e.g., Delphi process; 46%), and combined formal and informal approaches (e.g., virtual/in-person meetings or workshops; 33.3%) (Table S8).

**Table 1:** Summary of frameworks used in the development of TPPs and an overview of criteria used to select TPP features and their target levels according to appropriate criteria for product types. *Abbreviations* WHO: World Health Organization, TPP: target product profile, ICH: International Council for Harmonization, FDA: Federal Food and Drug Administration, FIND: Foundation for Innovative New Diagnostics, MSF: Médecins sans Frontières, HIV: human immunodeficiency virus

Framework	Number of TPP papers	References
ICH Q8 (R2).	14	[35, 50, 72, 73, 83, 102, 106, 113, 121, 127, 146–148, 152]
FIND TPPs.	7	[58, 62, 87, 100, 106, 119, 127]
WHO TPP.	4	[28, 87, 119, 142]
FDA guidance.	2	[50, 103]
TPP for dual HIV/syphilis tests commissioned in 2013 by UNITAID.	1	[25]
Previously published TPPs.	1	[28]
Published standards and guidelines, input from interviews.	1	[58]
Based on regulatory and practical considerations and limitations.	1	[75]
Own strategic framework, including two complementary TPPs: Target Market Profile (TMP) and Strategic Target Profile (STP).	1	[84]
MSF TPPs.	1	[142]
Own framework for discussion in regulatory and guideline development contexts.	1	[148]
**Criteria to select features**		
Literature review.	10	[31, 61, 62, 70, 72, 86, 93, 100, 128, 151]
Consensus agreement by a certain agreed percentage.	4	[87, 100, 119, 142]
Existing knowledge.	4	[32, 74, 83, 128]
Diagnostic accuracy parameters based on reference tests.	2	[64, 66]
Systematic review, published predictive models, landscape analysis.	1	[25]
Expert opinion.	1	[58]
Based on the requirements of the interested parties (clinical expectations, patients’ and industrial needs, regulatory aspects).	1	[114]

Most of the retrieved TPPs were published by authors from academia (56.5%), 11.6% by authors with non-academic affiliations, and 31.9% within hybrid collaborations. Twenty-seven (20%) of the papers mentioned which experts were involved, and their fields of expertise. These included academic researchers, clinicians, experts from the WHO, and stakeholders related to product development (end users, manufacturing companies, regulators) (Table S9). One group explicitly reported the involvement of civil societies representatives [93], but none reported involving patient populations or the public directly.

**Table 2:** TPP features most frequently reported in TPPs for different product types. The number of times each TPP feature was reported and the number of TPPs for each product type are indicated in brackets. A comprehensive list of TPP features is provided for each product type in Table S5

Diagnostics (29 TPP)	Drugs (57 TPP)	Vaccines (8 TPP)	Medical devices (3 TPP)	Other products (41 TPP)
Testing Sensitivity and specificity (*n* = 27)	Route of administration (*n* = 32)	Indication (*n* = 6)	Indication (*n* = 2)	Route of administration (*n* = 28)
Indication (*n* = 23)	Stability/shelf life (*n* = 29)	Target population (*n* = 4)	Cost of test/product/reimbursement (*n* = 2)	Stability/shelf life (*n* = 26)
Target population (*n* = 22)	Dosage form (*n* = 23)	Repeatability, stability (*n* = 4)	Data output (*n* = 2)	Dosage strength (*n* = 25)
Target user (*n* = 22)	Dosage strength (*n* = 23)	Storage conditions and shelf life (*n* = 3)	Accuracy (*n* = 2)	Dosage form (*n* = 23)
Sample type (*n* = 20)	Indication (*n* = 18)	Dose regimen and amount (*n* = 3)	Target user (*n* = 1)	Container closure system (*n* = 13)

Based on the overall extracted data, we identified essential aspects relevant to developing a new TPP, that would be good to report to enable full understanding and replication by other groups (Table 3).

**Table 3:** Key development steps and structural features (left column) relevant to understand and replicate the construction of TPPs. The right column reports possible specific items for the selected step, feature or category. These features are not exclusive, but rather add to those identified by Cocco et al., [13].

Key structural feature	Possible specific value determinants
1. Defining the purpose and perspective that the TPP will serve	e.g.,* Regulatory (meet regulatory requirements*,* e.g. evidence on validation); HTA (define competitive features in terms of cost-effectiveness); other.*
2. Choosing the appropriate framework to base the TPP on	e.g.,* ICH Q8 R2*,* WHO TPP guidance*,* FDA TPP guidance*,* other relevant framework for the specific product to be developed.*
3. Deciding on the approach/criteria to choose TPP features and their target levels	e.g.,* Criteria from previous WHO TPPs*,* criteria from specific regulations/guidance for the product*, etc.
4. Literature review to pool relevant TPP features	e.g.,* Systematic literature search.*
5. Formal consensus approach to agree on most critical TPP features	e.g.,* Delphi process.*
6. Classifying TPP features into categories	e.g.,* Using common categories appropriate for the field/product (e.g.*,* scope*,* test performance*,* operational characteristics for diagnostic tests).*
7. Involving relevant stakeholders:	e.g.: *- WHO experts. * *- Researchers in the field.* *- Manufacturers. * *- Regulators.* *- End users: clinicians*,* technicians*,* laboratory personnel*, etc. *- Public and/or Patient population.*

## Discussion

We conducted a systematic review to extract the features and structure of published TPPs across various therapeutic areas, to facilitate the incorporation of this tool in academic research. Most papers included in our review developed a new TPP, and mostly focused on developing treatments in the field of infectious diseases. Several medical fields (e.g., poliomyelitis, rheumatic fever, or tropical diseases) had only one TPP reported for any of their products. This happened also for diseases characterized by high unmet need and global prioritization, like the case of Alzheimer’s disease [57]. We found that the methods used to develop TPPs, their specific features, and the information provided to understand their structure varied considerably also within product or disease category, most papers providing limited or no explanation of how TPPs were developed.

To our knowledge, only one other systematic review on TPPs exists [13], focusing on diagnostic tests, and uniquely retrieving contributions for infectious diseases. Like us, Cocco and colleagues found a generally poor description of the methods employed to build TPPs, although they could extract a common 3-phase structure and the distinct dimensions of “activities”, “source of input information” and “contributing stakeholders”. The authors underlined that further research is needed to improve support for researchers in understanding and adopting the tool. In our review, we extended such examination beyond diagnostic tests, and tried to extract additional dimensions in TPP development that may further help to understand its general structure and logics (Table 3). Such dimensions may not be readily applicable to any field at present. For example, as a starting step, we recommend choosing a reference framework. However, as also noted by Cocco et al. [13], existing frameworks like the FDA guidance apply to drug development. They may not be directly transferable to diagnostics, and formal guidance on developing TPPs for diagnostics is not yet available. We believe that making this gap explicit and inviting researchers to identify a reference framework anyway may elicit consequent constructive steps. In this specific example, researchers developing a new TPP for diagnostics may decide to choose, as a reference framework, the structure of a previous TPP described in greater detail for a biomarker (see for example references 26, 28, 38 and 60 in Cocco’s paper); furthermore, outlining such gap explicitly may lead methodologists or other organizations or stakeholders to produce TPP guidance specifically adapted to diagnostics.

By widening the scope of therapeutic areas and products relative to the previous review, we sought to better outline the heterogeneity of TPPs, and extract more features contributing to their structure. To this regard, we underline that the TPPs scope is meant to be heterogeneous in nature, as they serve the development of specific products that need to differentiate themselves in the market. This needed heterogeneity adds to inconsistent reporting across the few documents that can escape confidentiality, which constitutes an additional hurdle to the effort of academic researchers to adopt the tool. On the other hand, a wide representation of different TPPs is needed to extract and communicate their very structure. In their review, Cocco et al. [13] not only focused on the field of diagnostics, and uniquely retrieved TPPs for infectious diseases, but also presented a quite consistent purpose of TPPs, mainly supporting the validation of diagnostic tests within a regulatory perspective. Despite our wider focus, also our study captured a mainly regulatory perspective. Indeed, a task like product validation can easily constitute a shared goal across independent research organizations, and can therefore be retrieved relatively easily in published documents. However, we underline here that TPPs are meant to support any development perspective (e.g., ensuring marketability, competitiveness or refundability within an HTA context). These perspectives, only to a limited degree captured in our review [84], may well be in the interest of academic developments as well. In industry, these other perspectives are usually represented in separate TPPs for the same product and each indication identified. We therefore underline that the mainly regulatory perspective emerging from our, as well as from Cocco’s, review is not the only nor the main purview of TPPs. Consistently, we do not support the idea that a TPP mainly serves single unitary purposes: indeed, they rather try to serve cross-functional aims, although they may not have one standard, coherent or all-inclusive form. This complexity enables the needed flexibility in operational contexts, but also makes it more difficult to understand the tool, for those who never used it. Finally, within the aim of demonstrating product validity, Cocco et al. highlighted a considerable absence of clinical utility features in TPPs for diagnostic products [13]; consistently, we found that, regardless of product type, the development of TPPs did not directly involve patient populations or the public, whose participation is important to define clinical significance in specific settings and demonstrate impact on clinically relevant outcomes. Different from academia, it is common in industrial practices to include patients or the public directly, and from the early stages of a product development; detailing this aspect in future TPP guidance may help upgrade academic product development and help it to manage the complex task of demonstrating clinical utility.

Overall, our extensive data extraction aimed to come up with a common structure helping academics to understand and use TPPs. Along with the features previously identified by Cocco et al., the items reported in Table 3 are general enough to be considered for inclusion in most TPPs, and relevant enough to be commendable for potential reporting guidelines on TPP development. Defining guidance as well as reporting recommendations requires independent dedicated efforts. With this work, we provided additional concrete elements for further initiatives supporting TPP integration in academic research. Such efforts are particularly urgent for diseases characterized by numerous unmet needs. Among these, the field of neurodegenerative diseases leading to dementia provides a concrete and current example: the 2017 Global Action Plan set goals to tackle the global priority of dementia [12], but the global status report on the public health response to dementia anticipated that these goals will not be achieved by the 2025 deadline [156]. WHO recommended using TPPs to boost efficiency in the field of infectious disease through a blueprint [157] recently provided also for dementia [11, 158]. Indeed, the use of tools like the TPP, already constituting good industrial practice, is increasingly relevant in any academic contexts, with an increasing interest in developing products also in the pharmacology field, and would support the efficiency of developing any kind of product, including medical procedures or services, by improving communication and interactions with industry and other stakeholders. Despite major dedicated efforts, like those supported by grant frameworks like the European Innovative Health Initiative, requiring that academics be paired with industry partners for large research projects, such interactions are still difficult, and concrete initiatives like ours, focusing on importing specific translational tools, methods and procedures, are essential to enable concrete steps forward. However, this overall picture raises pivotal questions about how to increase the efficiency of translational research. Which are exactly the stakeholders, collectively represented by WHO, supposedly interested in investing to translate and validate a reference methodology to develop TPPs, and promote their use in academic contexts across disease areas? Which incentives may encourage researchers to use them? Similar questions link our effort to the need to better understand and help improve the ecosystem of current translational research [159], where communication among academia, industry and relevant stakeholders is key to overcome gaps, and deserves greater attention.

Similar to the previous review, also our results show a striking majority of TPPs published for products in infectious diseases, with an exponential increase that, from the publication of the FDA Guidance for Industry guidelines in 2007, peaked during the COVID-19 epidemic. This prevalence of TPPs in the field of infectious diseases can be explained with the urgency to act and control rapidly spreading diseases [160], and can also be attributed to the successful implementation of the WHO blueprint [157]. The fact that we could identify many more publications in this rather than any other field also highlights the main constraint of our study: TPPs are usually confidential documents that cannot be circulated beyond the company producing them. The field of infectious diseases, however, may not be the most attractive area of development for industry: microbial resistance allows only limited time and distribution for a product to be effective, the treatment duration is very limited, antimicrobials’ price is generally low, and their need is mostly felt in countries with limited budget. Along with the highly unmet need posed by infectious diseases, a great public and academic involvement pushes the production of dedicated products, which may explain the disproportionate prevalence of public TPPs in this, compared to any other medical fields (Table S4). Some of the features characterizing the field of infectious diseases, like the increasing global unmet need with major distribution in LMICs, also apply to the field of dementia; this may provide additional motivation to greater adoption of TPPs in academic research in this field. On the other hand, developing TPPs in fields with similar features as that of neurodegenerative disorders may present more complex challenges. The etiology of complex diseases is often not definitively understood; genes, as well as their variable interaction with the environment, generate for example different degrees of cerebral reserve, and a number of factors interact with clinical outcomes and treatment effects; the urgency to bring innovation to clinics may lead to overlook validation steps for products or procedures, sometimes mistakenly not expected to originate negative effects, like biomarkers. Analogous considerations and analyses may help support the adoption of TPPs also in other such medical fields, where they are not yet widely used (Table S4). Moreover, the fact that independent laboratories may not be consistently aligned on a common translational methodology could provide additional rationale for producing shared and accessible TPPs, potentially benefitting all those working at a common goal. Indeed, the confidentiality protecting property for industry can well apply to academic research as well, thus it is relevant to identify the specific areas where the use of TPPs can be shared and possibly validated, to enable academics to familiarise with the methodology and then increase their use, either open or confidential. Further research in this direction may include retrieving TPPs developed for assets that subsequently failed, or by companies that are no longer active, and extract further learnings also leveraging reasons for failure.

Our findings indicate that TPP-related publications are mostly published by academics, although this finding is biased by the nature of this study. Different from the previous review [13], we only targeted full papers in scholar communications, using grey literature only to guide our understanding and framing of the data. We did find TPPs published by private organizations or public-private partnerships, however, by definition, we could only access information that was not confidential, an issue in common with the previous review [13]. We attenuated this bias by interviewing and involving some leading experts, all from industry contexts, able to provide a wider and more representative insight into this typically industrial procedure. We did not perform a formal assessment of risk of bias, however not feasible in this type of study, and did not extract data regarding the geographic location of TPP publications, connected with potential variations in unmet needs and priorities across health conditions, and consequently the product, depending on geography. From a methodological point of view, moreover, we guaranteed reliability only by assessing reviewers’ consistence on subsets of abstracts and full papers.

## Conclusion

This review highlights the heterogenous features of TPPs and their limited representation in academic literature besides the field of infectious diseases, and provides further concrete support for researchers trying to use TPPs in academic research. Our results can also feed future initiatives to adapt guidance for specific fields and to develop TPP reporting guidelines. Besides supporting researchers’ understanding and use of the tool in academic contexts, this would improve their ability to interact with regulators, HTA experts, and end users, whose contribution is needed along the whole translational continuum. Much more research is needed however to improve communication between academia and industry, stakeholder alignment, and the efficiency of academic translational research in general. Such efforts should help a wider understanding of the ecosystem and incentives structuring current translational research, and should be pursued to foster progress on global priorities.

### Electronic supplementary material

Below is the link to the electronic supplementary material.

Supplementary Material 1

Supplementary Material 2

## Data Availability

The datasets generated and analysed during the current study are available from the corresponding author on reasonable request.

## Abbreviations

AD: Alzheimer’s disease
FDA: Food and Drug Administration
HTA: Health Technology Assessment
ICH: International Conference on Harmonisation
NDD: Neurodegenerative diseases
PPP: Public-Private Partnership
QbD: Quality by design
TPP: Target product profile
WHO: World Health Organisation

## References

[1] FDA. Guidance for Industry and Review Staff Target Product Profile — A Strategic Development Process Tool. 2007.

[2] Organization WH. Links to TPP. https://www.who.int/observatories/global-observatory-on-health-research-and-development/analyses-and-syntheses/target-product-profile/who-target-product-profiles.

[3] Singh G. Target Product Profile and Clinical Development Plan. Pharmaceutical Medicine and Translational Clinical Research. Elsevier; 2018. pp. 65–80.

[4] Boccardi M. Translational process. J Transl Med. 2023;21(1):677. 37770943 10.1186/s12967-023-04507-7 PMC10540412

[5] Vemuri P, Wiste HJ, Weigand SD, Shaw LM, Trojanowski JQ, Weiner MW, et al. MRI and CSF biomarkers in normal, MCI, and AD subjects: predicting future clinical change. Neurology. 2009;73(4):294–301. 19636049 10.1212/WNL.0b013e3181af79fb PMC2715214

[6] Jack CR, Knopman DS, Jagust WJ, Shaw LM, Aisen PS, Weiner MW, et al. Hypothetical model of dynamic biomarkers of the Alzheimer’s pathological cascade. Lancet Neurol. 2010;9(1):119–28. 20083042 10.1016/S1474-4422(09)70299-6 PMC2819840

[7] Boccardi M, Gallo V, Yasui Y, Vineis P, Padovani A, Mosimann U, et al. The biomarker-based diagnosis of Alzheimer’s disease. 2-lessons from oncology. Neurobiol Aging. 2017;52:141–52. 28317645 10.1016/j.neurobiolaging.2017.01.021

[8] Pepe MS, Etzioni R, Feng Z, Potter JD, Thompson ML, Thornquist M, et al. Phases of biomarker development for early detection of cancer. J Natl Cancer Inst. 2001;93(14):1054–61. 11459866 10.1093/jnci/93.14.1054

[9] Fryback DG, Thornbury JR. The efficacy of diagnostic imaging. Med Decis Mak. 1991;11(2):88–94. 10.1177/0272989X9101100203 1907710

[10] Lijmer JG, Leeflang M, Bossuyt PMM. Proposals for a phased evaluation of medical tests. Med Decis Mak. 2009;29(5):E13–21. 10.1177/0272989X09336144 19605881

[11] Cataldi R, Chowdhary N, Seeher K, Moorthy V, Dua T. A blueprint for the worldwide research response to dementia. Lancet Neurol. 2022;21(8):690–1. 35841907 10.1016/S1474-4422(22)00269-1

[12] Organization WH. Global action plan on the public health response to dementia 2017–2025. 2017. https://www.who.int/publications/i/item/global-action-plan-on-the-public-health-response-to-dementia-2017---2025.

[13] Cocco P, Ayaz-Shah A, Messenger MP, West RM, Shinkins B. Target product profiles for medical tests: a systematic review of current methods. BMC Med. 2020;18(1):119. 32389127 10.1186/s12916-020-01582-1 PMC7212678

[14] Page MJ, McKenzie JE, Bossuyt PM, Boutron I, Hoffmann TC, Mulrow CD, et al. The PRISMA 2020 statement: an updated guideline for reporting systematic reviews. BMJ. 2021;372:n71. 33782057 10.1136/bmj.n71 PMC8005924

[15] Organization WH. Links to WHO TPPs and PPCs. https://www.who.int/observatories/global-observatory-on-health-research-and-development/analyses-and-syntheses/target-product-profile/links-to-who-tpps-and-ppcs.

[16] Team R, RStudio. Integrated Development for R. RStudio [Internet]. In: PBC, editor. Boston, MA2020.

[17] Chua A, Prat I, Nuebling CM, Wood D, Moussy F. Update on Zika Diagnostic tests and WHO’s related activities. PLoS Negl Trop Dis. 2017;11(2):e0005269. 28151953 10.1371/journal.pntd.0005269 PMC5289415

[18] Denkinger CM, Kik SV, Cirillo DM, Casenghi M, Shinnick T, Weyer K, et al. Defining the needs for next generation assays for tuberculosis. J Infect Dis. 2015;211(suppl2):S29–38. 25765104 10.1093/infdis/jiu821 PMC4447829

[19] Ding XC, Ade MP, Baird JK, Cheng Q, Cunningham J, Dhorda M, et al. Defining the next generation of Plasmodium Vivax diagnostic tests for control and elimination: target product profiles. PLoS Negl Trop Dis. 2017;11(4):e0005516. 28369085 10.1371/journal.pntd.0005516 PMC5391123

[20] Donadeu M, Fahrion AS, Olliaro PL, Abela-Ridder B. Target product profiles for the diagnosis of Taenia solium taeniasis, neurocysticercosis and porcine cysticercosis. PLoS Negl Trop Dis. 2017;11(9):e0005875. 28892472 10.1371/journal.pntd.0005875 PMC5608417

[21] Ebels KB, Clerk C, Crudder CH, McGray S, Magnuson K, Tietje K et al. editors. Incorporating user needs into product development for improved infection detection for malaria elimination programs. 2014 IEEE Global Humanitarian Technology Conference (GHTC); 2014 2014/10//. San Jose, CA: IEEE.

[22] Lim MD, Brooker SJ, Belizario VY, Gay-Andrieu F, Gilleard J, Levecke B, et al. Diagnostic tools for soil-transmitted helminths control and elimination programs: a pathway for diagnostic product development. PLoS Negl Trop Dis. 2018;12(3):e0006213. 29494581 10.1371/journal.pntd.0006213 PMC5832200

[23] Pal S, Jasper LE, Lawrence KL, Walter M, Gilliland T, Dauner AL, et al. Assessing the Dengue Diagnosis Capability Gap in the Military Health System. Mil Med. 2016;181(8):756–66. 27483511 10.7205/MILMED-D-15-00231

[24] Solomon AW, Engels D, Bailey RL, Blake IM, Brooker S, Chen J-X, et al. A Diagnostics platform for the Integrated Mapping, Monitoring, and Surveillance of Neglected Tropical diseases: Rationale and Target Product profiles. PLoS Negl Trop Dis. 2012;6(7):e1746. 22860146 10.1371/journal.pntd.0001746 PMC3409112

[25] Toskin I, Murtagh M, Peeling RW, Blondeel K, Cordero J, Kiarie J. Advancing prevention of sexually transmitted infections through point-of-care testing: target product profiles and landscape analysis. Sex Transm Infect. 2017;93(S4):S69–80. 29223965 10.1136/sextrans-2016-053071

[26] Utzinger J, Becker SL, Van Lieshout L, Van Dam GJ, Knopp S. New diagnostic tools in schistosomiasis. Clin Microbiol Infect. 2015;21(6):529–42. 25843503 10.1016/j.cmi.2015.03.014

[27] Abdulgader SM, Okunola AO, Ndlangalavu G, Reeve BWP, Allwood BW, Koegelenberg CFN, et al. Diagnosing tuberculosis: what do New Technologies allow us to (not). Do? Respiration. 2022;101(9):797–813. 35760050 10.1159/000525142 PMC9533455

[28] Adepoyibi T, Lilis L, Greb H, Boyle D. Which attributes within target product profiles for tuberculosis diagnostics are the most important to focus on? int j Tuberc lung dis. 2018;22(4):425–8. 29562991 10.5588/ijtld.17.0312

[29] Alafeef M, Pan D. Diagnostic approaches for COVID-19: lessons learned and the path Forward. ACS Nano. 2022;16(8):11545–76. 35921264 10.1021/acsnano.2c01697

[30] Alonso-Padilla J, Abril M, De Alarcón B, Almeida IC, Angheben A, Araujo Jorge T, et al. Target product profile for a test for the early assessment of treatment efficacy in Chagas disease patients: an expert consensus. PLoS Negl Trop Dis. 2020;14(4):e0008035. 32324735 10.1371/journal.pntd.0008035 PMC7179829

[31] Amasya G, Badilli U, Aksu B, Tarimci N. Quality by design case study 1: design of 5-fluorouracil loaded lipid nanoparticles by the W/O/W double emulsion — solvent evaporation method. Eur J Pharm Sci. 2016;84:92–102. 26780593 10.1016/j.ejps.2016.01.003

[32] Apolinário AC, Ferraro RB, De Oliveira CA, Pessoa A Jr, De Oliveira Rangel-Yagui C. Quality-by-Design Approach for Biological API encapsulation into Polymersomes using off-the-Shelf materials: a study on L-Asparaginase. AAPS PharmSciTech. 2019;20(6):251. 31300911 10.1208/s12249-019-1465-1

[33] Arnold SLM. Target Product Profile and Development path for Shigellosis Treatment with antibacterials. ACS Infect Dis. 2021;7(5):948–58. 33689318 10.1021/acsinfecdis.0c00889

[34] Arora D, Nanda S. Quality by design driven development of resveratrol loaded ethosomal hydrogel for improved dermatological benefits via enhanced skin permeation and retention. Int J Pharm. 2019;567:118448. 31226472 10.1016/j.ijpharm.2019.118448

[35] Arranja A, Gouveia LF, Gener P, Rafael DF, Pereira C, Schwartz S, et al. Self-assembly PEGylation assists SLN-paclitaxel delivery inducing cancer cell apoptosis upon internalization. Int J Pharm. 2016;501(1–2):180–9. 26853316 10.1016/j.ijpharm.2016.01.075

[36] Awotwe-Otoo D, Agarabi C, Wu GK, Casey E, Read E, Lute S, et al. Quality by design: impact of formulation variables and their interactions on quality attributes of a lyophilized monoclonal antibody. Int J Pharm. 2012;438(1–2):167–75. 22944306 10.1016/j.ijpharm.2012.08.033

[37] Beg S, Katare OP, Singh B. Formulation by design approach for development of ultrafine self-nanoemulsifying systems of rosuvastatin calcium containing long-chain lipophiles for hyperlipidemia management. Colloids Surf B. 2017;159:869–79. 10.1016/j.colsurfb.2017.08.050 28892871

[38] Beg S, Saini S, Bandopadhyay S, Katare OP, Singh B. QbD-driven development and evaluation of nanostructured lipid carriers (NLCs) of Olmesartan medoxomil employing multivariate statistical techniques. Drug Dev Ind Pharm. 2018;44(3):407–20. 29048242 10.1080/03639045.2017.1395459

[39] Beg S, Sandhu PS, Batra RS, Khurana RK, Singh B. QbD-based systematic development of novel optimized solid self-nanoemulsifying drug delivery systems (SNEDDS) of lovastatin with enhanced biopharmaceutical performance. Drug Delivery. 2015;22(6):765–84. 24673611 10.3109/10717544.2014.900154

[40] Bell M, Webster L, Woodland A. Research Techniques made simple: an introduction to Drug Discovery for Dermatology. J Invest Dermatology. 2019;139(11):2252–e71. 10.1016/j.jid.2019.07.699 31648685

[41] Bengtson M, Bharadwaj M, Bosch AT, Nyakundi H, Matoke-Muhia D, Dekker C, et al. Matching development of point-of-care diagnostic tests to the local context: a case study of visceral leishmaniasis in Kenya and Uganda. Glob Health Sci Pract. 2020;8(3):549–65. 33008863 10.9745/GHSP-D-20-00028 PMC7541118

[42] Bergström F, Lindmark B. Accelerated drug discovery by rapid candidate drug identification. Drug Discovery Today. 2019;24(6):1237–41. 30946980 10.1016/j.drudis.2019.03.026

[43] Bernatchez JA, Tran LT, Li J, Luan Y, Siqueira-Neto JL, Li R. Drugs for the treatment of Zika Virus infection. J Med Chem. 2020;63(2):470–89. 31549836 10.1021/acs.jmedchem.9b00775 PMC12753316

[44] Burrows JN, Duparc S, Gutteridge WE, Van Hooft R, Kaszubska W, Macintyre F, et al. New developments in anti-malarial target candidate and product profiles. Malar J. 2017;16(1):26. 28086874 10.1186/s12936-016-1675-x PMC5237200

[45] Burrows J, Slater H, Macintyre F, Rees S, Thomas A, Okumu F, et al. A discovery and development roadmap for new endectocidal transmission-blocking agents in malaria. Malar J. 2018;17(1):462. 30526594 10.1186/s12936-018-2598-5 PMC6287360

[46] Campbell A, Brieva T, Raviv L, Rowley J, Niss K, Brandwein H, et al. Concise review: process development considerations for cell therapy. Stem Cells Translational Med. 2015;4(10):1155–63. 10.5966/sctm.2014-0294 PMC4572896 26315572

[47] Carballar-Lejarazú R, Ogaugwu C, Tushar T, Kelsey A, Pham TB, Murphy J, et al. Next-generation gene drive for population modification of the malaria vector mosquito, <i > Anopheles gambiae</i >. Proc Natl Acad Sci USA. 2020;117(37):22805–14. 32839345 10.1073/pnas.2010214117 PMC7502704

[48] Chang R-K, Raw A, Lionberger R, Yu L. Generic development of topical dermatologic products, part II: quality by design for Topical Semisolid products. AAPS J. 2013;15(3):674–83. 23572241 10.1208/s12248-013-9472-8 PMC3691439

[49] Chappidi SR, Bhargav E, Marikunte V, Chinthaginjala H, Vijaya Jyothi M, Pisay M, et al. A cost effective (QbD) Approach in the Development and Optimization of Rosiglitazone Maleate Mucoadhesive Extended Release tablets – in Vitro and Ex vivo. Adv Pharm Bull. 2019;9(2):281–8. 31380254 10.15171/apb.2019.032 PMC6664114

[50] Charoo NA, Shamsher AAA, Zidan AS, Rahman Z. Quality by design approach for formulation development: a case study of dispersible tablets. Int J Pharm. 2012;423(2):167–78. 22209997 10.1016/j.ijpharm.2011.12.024

[51] Chudiwal SS, Dehghan MHG. Quality by design approach for development of suspension nasal spray products: a case study on budesonide nasal suspension. Drug Dev Ind Pharm. 2016;42(10):1643–52. 26943653 10.3109/03639045.2016.1160108

[52] Chudiwal SS, Dehghan MHG. Quality by design (QbD) approach for design and development of drug-device combination products: a case study on flunisolide nasal spray. Pharm Dev Technol. 2018;23(10):1077–87. 27616074 10.1080/10837450.2016.1236130

[53] Chudiwal VS, Shahi S, Chudiwal S. Development of sustained release gastro-retentive tablet formulation of nicardipine hydrochloride using quality by design (QbD) approach. Drug Dev Ind Pharm. 2018;44(5):787–99. 29198152 10.1080/03639045.2017.1413111

[54] Costa CP, Cunha S, Moreira JN, Silva R, Gil-Martins E, Silva V, et al. Quality by design (QbD) optimization of diazepam-loaded nanostructured lipid carriers (NLC) for nose-to-brain delivery: toxicological effect of surface charge on human neuronal cells. Int J Pharm. 2021;607:120933. 34324988 10.1016/j.ijpharm.2021.120933

[55] Crcarevska M, Dimitrovska A, Sibinovska N, Mladenovska K, Slavevska Raicki R, Glavas Dodov M. Implementation of quality by design principles in the development of microsponges as drug delivery carriers: identification and optimization of critical factors using multivariate statistical analyses and design of experiments studies. Int J Pharm. 2015;489(1–2):58–72. 25895722 10.1016/j.ijpharm.2015.04.038

[56] Cruz I, Albertini A, Barbeitas M, Arana B, Picado A, Ruiz-Postigo JA, et al. Target Product Profile for a point-of-care diagnostic test for dermal leishmaniases. Parasite Epidemiol Control. 2019;5:e00103. 30923755 10.1016/j.parepi.2019.e00103 PMC6423987

[57] Cunha S, Costa CP, Loureiro JA, Alves J, Peixoto AF, Forbes B, et al. Double optimization of Rivastigmine-loaded nanostructured lipid carriers (NLC) for nose-to-brain delivery using the quality by Design (QbD) Approach: formulation variables and instrumental parameters. Pharmaceutics. 2020;12(7):599. 32605177 10.3390/pharmaceutics12070599 PMC7407548

[58] Dailey P, Osborn J, Ashley E, Baron E, Dance D, Fusco D, et al. Defining System requirements for simplified blood culture to enable widespread use in resource-limited settings. Diagnostics. 2019;9(1):10. 30641976 10.3390/diagnostics9010010 PMC6468589

[59] Dalal R, Shah J, Gorain B, Choudhury H, Jacob S, Mehta TA, et al. Development and optimization of Asenapine Sublingual Film using QbD Approach. AAPS PharmSciTech. 2021;22(7):244. 34608546 10.1208/s12249-021-02132-5

[60] Deng Y, Zhong G, Wang Y, Wang N, Yu Q, Yu X. Quality by design approach for the preparation of fat-soluble vitamins lipid injectable emulsion. Int J Pharm. 2019;571:118717. 31610279 10.1016/j.ijpharm.2019.118717

[61] Denkinger CM, Dolinger D, Schito M, Wells W, Cobelens F, Pai M, et al. Target Product Profile of a Molecular Drug-Susceptibility Test for Use in Microscopy centers. J Infect Dis. 2015;211(suppl2):S39–49. 25765105 10.1093/infdis/jiu682 PMC4425821

[62] Dittrich S, Tadesse BT, Moussy F, Chua A, Zorzet A, Tängdén T, et al. Target Product Profile for a diagnostic assay to differentiate between bacterial and non-bacterial infections and reduce antimicrobial overuse in Resource-Limited settings: an Expert Consensus. PLoS ONE. 2016;11(8):e0161721. 27559728 10.1371/journal.pone.0161721 PMC4999186

[63] Dormenval C, Lokras A, Cano-Garcia G, Wadhwa A, Thanki K, Rose F, et al. Identification of factors of importance for spray drying of small interfering RNA-Loaded lipidoid-polymer hybrid nanoparticles for inhalation. Pharm Res. 2019;36(10):142. 31376020 10.1007/s11095-019-2663-y

[64] Fongwen N, Wilder-Smith A, Gubler DJ, Ooi EE, Salvana T, De Lamballerie EM. Target product profile for a dengue pre-vaccination screening test. PLoS Negl Trop Dis. 2021;15(7):e0009557. 34324505 10.1371/journal.pntd.0009557 PMC8320982

[65] Funk CD, Laferrière C, Ardakani A. A snapshot of the global race for vaccines targeting SARS-CoV-2 and the COVID-19 pandemic. Front Pharmacol. 2020;11:937. 32636754 10.3389/fphar.2020.00937 PMC7317023

[66] Gal M, Francis NA, Hood K, Villacian J, Goossens H, Watkins A, et al. Matching diagnostics development to clinical need: target product profile development for a point of care test for community-acquired lower respiratory tract infection. PLoS ONE. 2018;13(8):e0200531. 30067760 10.1371/journal.pone.0200531 PMC6070214

[67] García-Basteiro AL, DiNardo A, Saavedra B, Silva DR, Palmero D, Gegia M, et al. Point of care diagnostics for tuberculosis. Pulmonology. 2018;24(2):73–85. 29426581 10.1016/j.rppnen.2017.12.002

[68] Garg B, Katare OP, Beg S, Lohan S, Singh B. Systematic development of solid self-nanoemulsifying oily formulations (S-SNEOFs) for enhancing the oral bioavailability and intestinal lymphatic uptake of lopinavir. Colloids Surf B. 2016;141:611–22. 10.1016/j.colsurfb.2016.02.012 26916320

[69] Garg S, Tambwekar KR, Vermani K, Kandarapu R, Garg A, Waller DP, et al. Development Pharmaceutics of Microbicide formulations. Part II: formulation, evaluation, and challenges. AIDS Patient Care STDs. 2003;17(8):377–99. 13678540 10.1089/108729103322277402

[70] Gavan A, Porfire A, Marina C, Tomuta I. Formulation and pharmaceutical development of quetiapine fumarate sustained release matrix tablets using a QbD approach. Acta Pharm. 2017;67(1):53–70. 28231048 10.1515/acph-2017-0009

[71] Ghaffari A, Meurant R, Ardakani A. COVID-19 point-of-Care Diagnostics that Satisfy Global Target Product profiles. Diagnostics. 2021;11(1):115. 33445727 10.3390/diagnostics11010115 PMC7828180

[72] Gurumukhi VC, Bari SB. Quality by design (QbD)–based fabrication of atazanavir-loaded nanostructured lipid carriers for lymph targeting: bioavailability enhancement using chylomicron flow block model and toxicity studies. Drug Deliv Transl Res. 2022;12(5):1230–52. 34110597 10.1007/s13346-021-01014-4

[73] Gurumukhi VC, Bari SB. Development of ritonavir-loaded nanostructured lipid carriers employing quality by design (QbD) as a tool: characterizations, permeability, and bioavailability studies. Drug Deliv Transl Res. 2022;12(7):1753–73. 34671949 10.1007/s13346-021-01083-5

[74] Ha J-M, Seo J-W, Kim S-H, Kim J-Y, Park C-W, Rhee Y-S, et al. Implementation of quality by design for Formulation of Rebamipide Gastro-retentive tablet. AAPS PharmSciTech. 2017;18(8):3129–39. 28526986 10.1208/s12249-017-0797-y

[75] Hales D, Vlase L, Porav SA, Bodoki A, Barbu-Tudoran L, Achim M, et al. A quality by design (QbD) study on enoxaparin sodium loaded polymeric microspheres for colon-specific delivery. Eur J Pharm Sci. 2017;100:249–61. 28088371 10.1016/j.ejps.2017.01.006

[76] Hastings IM, Hodel EM. Pharmacological considerations in the design of anti-malarial drug combination therapies – is matching half-lives enough? Malar J. 2014;13(1):62. 24552440 10.1186/1475-2875-13-62 PMC3975950

[77] Heal DJ, Smith SL. Prospects for new drugs to treat binge-eating disorder: insights from psychopathology and neuropharmacology. J Psychopharmacol. 2022;36(6):680–703. 34318734 10.1177/02698811211032475 PMC9150143

[78] Hernandez-Morales I, Van Loock M. An industry perspective on Dengue Drug Discovery and Development. In: Hilgenfeld R, Vasudevan SG, editors. Dengue and Zika: control and antiviral treatment strategies. Volume 1062. Singapore: Springer Singapore; 2018. pp. 333–53. 10.1007/978-981-10-8727-1_23 29845543

[79] Huston CD, Spangenberg T, Burrows J, Willis P, Wells TNC, Van Voorhis W. A proposed Target Product Profile and Developmental Cascade for New Cryptosporidiosis treatments. PLoS Negl Trop Dis. 2015;9(10):e0003987. 26447884 10.1371/journal.pntd.0003987 PMC4598153

[80] Ignjatović J, Đuriš J, Cvijić S, Dobričić V, Montepietra A, Lombardi C, et al. Development of solid lipid microparticles by melt-emulsification/spray-drying processes as carriers for pulmonary drug delivery. Eur J Pharm Sci. 2021;156:105588. 33045367 10.1016/j.ejps.2020.105588

[81] Ingvarsson PT, Yang M, Mulvad H, Nielsen HM, Rantanen J, Foged C. Engineering of an inhalable DDA/TDB liposomal adjuvant: a quality-by-design Approach towards optimization of the spray drying process. Pharm Res. 2013;30(11):2772–84. 23794038 10.1007/s11095-013-1096-2

[82] Ivanova Reipold E, Easterbrook P, Trianni A, Panneer N, Krakower D, Ongarello S, et al. Optimising diagnosis of viraemic hepatitis C infection: the development of a target product profile. BMC Infect Dis. 2017;17(S1):707. 29143620 10.1186/s12879-017-2770-5 PMC5688443

[83] Jaffar-Aghaei M, Khanipour F, Maghsoudi A, Sarvestani R, Mohammadian M, Maleki M, et al. QbD-guided pharmaceutical development of Pembrolizumab biosimilar candidate PSG-024 propelled to industry meeting primary requirements of comparability to Keytruda^®^. Eur J Pharm Sci. 2022;173:106171. 35378209 10.1016/j.ejps.2022.106171

[84] Jambulingam T. The R&D Marketing Interface in Biopharma and MedTech. JCB. 2019;24(4).

[85] Javed MN, Kohli K, Amin S. Risk Assessment Integrated QbD Approach for Development of Optimized Bicontinuous Mucoadhesive Limicubes for oral delivery of Rosuvastatin. AAPS PharmSciTech. 2018;19(3):1377–91. 29388027 10.1208/s12249-018-0951-1

[86] Joshi M, Yadav KS, Prabhakar B. Quality by Design Approach for Development and Optimization of Rifampicin Loaded Bovine Serum Albumin Nanoparticles and characterization. CDD. 2021;18(9):1338–51. 10.2174/1567201818666210212090451 33583375

[87] Kadam R, White W, Banks N, Katz Z, Dittrich S, Kelly-Cirino C. Target Product Profile for a mobile app to read rapid diagnostic tests to strengthen infectious disease surveillance. PLoS ONE. 2020;15(1):e0228311. 31995628 10.1371/journal.pone.0228311 PMC6988927

[88] Kakade P, Gite S, Patravale V. Development of Atovaquone Nanosuspension: quality by Design Approach. CDD. 2020;17(2):112–25. 10.2174/1567201817666191227095019 31880260

[89] Kraan H, Van Der Stel W, Kersten G, Amorij J-P. Alternative administration routes and delivery technologies for polio vaccines. Expert Rev Vaccines. 2016;15(8):1029–40. 26912100 10.1586/14760584.2016.1158650

[90] Kuk D-H, Ha E-S, Ha D-H, Sim W-Y, Lee S-K, Jeong J-S, et al. Development of a Resveratrol Nanosuspension using the Antisolvent Precipitation Method without Solvent removal, based on a quality by Design (QbD) Approach. Pharmaceutics. 2019;11(12):688. 31861173 10.3390/pharmaceutics11120688 PMC6955680

[91] Lambert WJ. Considerations in developing a Target Product Profile for Parenteral Pharmaceutical products. AAPS PharmSciTech. 2010;11(3):1476–81. 20842540 10.1208/s12249-010-9521-x PMC2974137

[92] Leng D, Thanki K, Fattal E, Foged C, Yang M. Engineering of budesonide-loaded lipid-polymer hybrid nanoparticles using a quality-by-design approach. Int J Pharm. 2018;548(2):740–6. 28847667 10.1016/j.ijpharm.2017.08.094

[93] Lewin SR, Attoye T, Bansbach C, Doehle B, Dubé K, Dybul M, et al. Multi-stakeholder consensus on a target product profile for an HIV cure. Lancet HIV. 2021;8(1):e42–50. 33271125 10.1016/S2352-3018(20)30234-4 PMC7773628

[94] Lokras A, Thakur A, Wadhwa A, Thanki K, Franzyk H, Foged C. Optimizing the intracellular delivery of therapeutic anti-inflammatory TNF-α siRNA to activated macrophages using lipidoid-polymer hybrid nanoparticles. Front Bioeng Biotechnol. 2021;8:601155. 33520957 10.3389/fbioe.2020.601155 PMC7841370

[95] Macintyre F, Ramachandruni H, Burrows JN, Holm R, Thomas A, Möhrle JJ, et al. Injectable anti-malarials revisited: discovery and development of new agents to protect against malaria. Malar J. 2018;17(1):402. 30384848 10.1186/s12936-018-2549-1 PMC6211409

[96] Malvolti S, Malhame M, Mantel CF, Le Rutte EA, Kaye PM. Human leishmaniasis vaccines: use cases, target population and potential global demand. PLoS Negl Trop Dis. 2021;15(9):e0009742. 34547025 10.1371/journal.pntd.0009742 PMC8486101

[97] Manjunatha UH, Chao AT, Leong FJ, Diagana TT. Cryptosporidiosis Drug Discovery: opportunities and challenges. ACS Infect Dis. 2016;2(8):530–7. 27626293 10.1021/acsinfecdis.6b00094

[98] Martín-Escolano J, Medina-Carmona E, Martín-Escolano R. Chagas Disease: current view of an ancient and global chemotherapy challenge. ACS Infect Dis. 2020;6(11):2830–43. 33034192 10.1021/acsinfecdis.0c00353

[99] Mateus D, Marto J, Trindade P, Gonçalves H, Salgado A, Machado P, et al. Improved morphine-loaded hydrogels for Wound-Related Pain Relief. Pharmaceutics. 2019;11(2):76. 30759886 10.3390/pharmaceutics11020076 PMC6409998

[100] Mather RG, Hopkins H, Parry CM, Dittrich S. Redefining typhoid diagnosis: what would an improved test need to look like? BMJ Glob Health. 2019;4(5):e001831. 31749999 10.1136/bmjgh-2019-001831 PMC6830052

[101] Mercuri AM. Quality by Design Applied to Ocular solid lipid nanoparticles containing a hydrophilic peptide prepared via Hot High pressure Homogeniser. CDD. 2016;13(8):1247–60. 10.2174/1567201813666160325131831 27012669

[102] Mirani AG, Patankar SP, Kadam VJ. Risk-based approach for systematic development of gastroretentive drug delivery system. Drug Deliv Transl Res. 2016;6(5):579–96. 27468861 10.1007/s13346-016-0315-x

[103] Mishra SM, Rohera BD. An integrated, quality by design (QbD) approach for design, development and optimization of orally disintegrating tablet formulation of carbamazepine. Pharm Dev Technol. 2017;22(7):889–903. 27346282 10.1080/10837450.2016.1199566

[104] Mo AX, Colley DG. Workshop report: Schistosomiasis vaccine clinical development and product characteristics. Vaccine. 2016;34(8):995–1001. 26721329 10.1016/j.vaccine.2015.12.032

[105] Monath TP, Kortekaas J, Watts DM, Christofferson RC, Desiree LaBeaud A, Gowen BB et al. Theoretical risk of genetic reassortment should not impede development of live, attenuated Rift Valley fever (RVF) vaccines commentary on the draft WHO RVF Target Product Profile. Vaccine: X. 2020;5:100060. 10.1016/j.jvacx.2020.100060 PMC7176985 32337506

[106] Nakas A, Dalatsi AM, Kapourani A, Kontogiannopoulos KN, Assimopoulou AN, Barmpalexis P. Quality Risk Management and Quality by Design for the Development of Diclofenac Sodium Intra-articular Gelatin Microspheres. AAPS PharmSciTech. 2020;21(4):127. 32390062 10.1208/s12249-020-01678-0

[107] Namjoshi S, Dabbaghi M, Roberts MS, Grice JE, Mohammed Y. Quality by design: development of the Quality Target Product Profile (QTPP) for Semisolid Topical products. Pharmaceutics. 2020;12(3):287. 32210126 10.3390/pharmaceutics12030287 PMC7150996

[108] Nazari K, Mehta P, Arshad MS, Ahmed S, Andriotis EG, Singh N, et al. Quality by Design Micro-engineering Optimisation of NSAID-Loaded Electrospun Fibrous patches. Pharmaceutics. 2019;12(1):2. 31861296 10.3390/pharmaceutics12010002 PMC7022274

[109] Neale G, Gaihre S, O’Gorman P, Price RK, Balzategi AG, Barrientos CH, et al. Review of recent innovations in portable child growth measurement devices for use in low- and middle-income countries. J Med Eng Technol. 2021;45(8):642–55. 34309474 10.1080/03091902.2021.1946181

[110] Németh Z, Pallagi E, Dobó DG, Csóka I. A proposed methodology for a Risk Assessment-based Liposome Development process. Pharmaceutics. 2020;12(12):1164. 33260443 10.3390/pharmaceutics12121164 PMC7760874

[111] Nsanzabana C, Ariey F, Beck H-P, Ding XC, Kamau E, Krishna S, et al. Molecular assays for antimalarial drug resistance surveillance: a target product profile. PLoS ONE. 2018;13(9):e0204347. 30235327 10.1371/journal.pone.0204347 PMC6147503

[112] Oh G-H, Park J-H, Shin H-W, Kim J-E, Park Y-J. Quality-by-design approach for the development of telmisartan potassium tablets. Drug Dev Ind Pharm. 2018;44(5):837–48. 29252038 10.1080/03639045.2017.1414233

[113] Pallagi E, Ambrus R, Szabó-Révész P, Csóka I. Adaptation of the quality by design concept in early pharmaceutical development of an intranasal nanosized formulation. Int J Pharm. 2015;491(1–2):384–92. 26134895 10.1016/j.ijpharm.2015.06.018

[114] Pallagi E, Jójárt-Laczkovich O, Németh Z, Szabó-Révész P, Csóka I. Application of the QbD-based approach in the early development of liposomes for nasal administration. Int J Pharm. 2019;562:11–22. 30877028 10.1016/j.ijpharm.2019.03.021

[115] Patadia R, Vora C, Mittal K, Mashru RC. Quality by Design Empowered Development and Optimisation of Time-controlled pulsatile release platform Formulation employing Compression Coating Technology. AAPS PharmSciTech. 2017;18(4):1213–27. 27460936 10.1208/s12249-016-0590-3

[116] Patel GM, Shelat PK, Lalwani AN. QbD based development of proliposome of lopinavir for improved oral bioavailability. Eur J Pharm Sci. 2017;108:50–61. 27586019 10.1016/j.ejps.2016.08.057

[117] Patel HP, Chaudhari PS, Gandhi PA, Desai BV, Desai DT, Dedhiya PP, et al. Nose to brain delivery of tailored clozapine nanosuspension stabilized using (+)-alpha-tocopherol polyethylene glycol 1000 succinate: optimization and in vivo pharmacokinetic studies. Int J Pharm. 2021;600:120474. 33737093 10.1016/j.ijpharm.2021.120474

[118] Patel H, Patel K, Tiwari S, Pandey S, Shah S, Gohel M. Quality by Design (QbD) Approach for Development of Co-processed Excipient Pellets (MOMLETS) by extrusion-spheronization technique. DDF. 2016;10(3):192–206. 10.2174/1872211310666160709193540 27396400

[119] Pellé KG, Rambaud-Althaus C, D’Acremont V, Moran G, Sampath R, Katz Z, et al. Electronic clinical decision support algorithms incorporating point-of-care diagnostic tests in low-resource settings: a target product profile. BMJ Glob Health. 2020;5(2):e002067. 32181003 10.1136/bmjgh-2019-002067 PMC7050342

[120] Peraman R, Bhadraya K, Reddy Y, Reddy C, Lokesh T. Analytical quality by design approach in RP-HPLC method development for the assay of etofenamate in dosage forms. Indian J Pharm Sci. 2015;77(6):751. 26997704 10.4103/0250-474X.174971 PMC4778236

[121] Perrier Q, Piquemal M, Leenhardt J, Choisnard L, Mazet R, Desruet M-D, et al. A quality by design approach for the qualification of automating compounding device for parenteral nutrition. Eur J Pharm Sci. 2022;179:106275. 35987326 10.1016/j.ejps.2022.106275

[122] Porrás AI, Yadon ZE, Altcheh J, Britto C, Chaves GC, Flevaud L, et al. Target Product Profile (TPP) for Chagas Disease Point-of-care diagnosis and Assessment of response to treatment. PLoS Negl Trop Dis. 2015;9(6):e0003697. 26042730 10.1371/journal.pntd.0003697 PMC4456144

[123] Reinhard-Rupp J, Klohe K. Developing a comprehensive response for treatment of children under 6 years of age with schistosomiasis: research and development of a pediatric formulation of praziquantel. Infect Dis Poverty. 2017;6(1):122. 28768535 10.1186/s40249-017-0336-9 PMC5541653

[124] Romano J, Manning J, Hemmerling A, McGrory E, Young Holt B. Prioritizing multipurpose prevention technology development and investments using a target product profile. Antiviral Res. 2013;100:S32–8. 24188707 10.1016/j.antiviral.2013.09.016

[125] Russell C, Hussain M, Huen D, Rahman AS, Mohammed AR. Profiling gene expression dynamics underpinning conventional testing approaches to better inform pre-clinical evaluation of an age appropriate spironolactone formulation. Pharm Dev Technol. 2021;26(1):101–9. 33078682 10.1080/10837450.2020.1839496

[126] Salami K, Gsell P-S, Olayinka A, Maiga D, Formenty P, Smith PG, et al. Meeting report: WHO consultation on accelerating Lassa fever vaccine development in endemic countries, Dakar, 10–11 September 2019. Vaccine. 2020;38(26):4135–41. 31952873 10.1016/j.vaccine.2020.01.017

[127] Saydam M, Takka S. Development and < i > in vitro evaluation of pH-independent release matrix tablet of weakly acidic drug valsartan using quality by design tools. Drug Dev Ind Pharm. 2018;44(12):1905–17. 29969042 10.1080/03639045.2018.1496450

[128] Simões A, Veiga F, Figueiras A, Vitorino C. A practical framework for implementing quality by design to the development of topical drug products: Nanosystem-based dosage forms. Int J Pharm. 2018;548(1):385–99. 29953928 10.1016/j.ijpharm.2018.06.052

[129] Simões A, Veiga F, Vitorino C, Figueiras A. A Tutorial for developing a topical cream formulation based on the quality by Design Approach. J Pharm Sci. 2018;107(10):2653–62. 29935297 10.1016/j.xphs.2018.06.010

[130] Singh B, Kaur A, Dhiman S, Garg B, Khurana RK, Beg S. QbD-Enabled development of Novel Stimuli-Responsive Gastroretentive systems of Acyclovir for Improved Patient Compliance and Biopharmaceutical Performance. AAPS PharmSciTech. 2016;17(2):454–65. 26238805 10.1208/s12249-015-0367-0 PMC4984879

[131] Staunton KM, Liu J, Townsend M, Desnoyer M, Howell P, Crawford JE, et al. Designing Aedes (Diptera: Culicidae) Mosquito traps: the evolution of the male Aedes sound trap by iterative evaluation. Insects. 2021;12(5):388. 33925425 10.3390/insects12050388 PMC8146609

[132] Swindells S, Siccardi M, Barrett SE, Olsen DB, Grobler JA, Podany AT, et al. Long-acting formulations for the treatment of latent tuberculous infection: opportunities and challenges. int j Tuberc lung dis. 2018;22(2):125–32. 29506608 10.5588/ijtld.17.0486 PMC6103451

[133] Sylvester B, Tefas L, Vlase L, Tomuţă I, Porfire A. A quality by design (QbD) approach to the development of a gradient high-performance liquid chromatography for the simultaneous assay of curcuminoids and doxorubicin from long-circulating liposomes. J Pharm Biomed Anal. 2018;158:395–404. 29966945 10.1016/j.jpba.2018.06.018

[134] Taipale-Kovalainen K, Karttunen A-P, Ketolainen J, Korhonen O. Lubricant based determination of design space for continuously manufactured high dose Paracetamol tablets. Eur J Pharm Sci. 2018;115:1–10. 29277668 10.1016/j.ejps.2017.12.021

[135] Tanaka T, Hanaoka H, Sakurai S. Optimization of the quality by design approach for gene therapy products: a case study for adeno-associated viral vectors. Eur J Pharm Biopharm. 2020;155:88–102. 32784043 10.1016/j.ejpb.2020.08.002

[136] Thakkar R, Ashour EA, Shukla A, Wang R, Chambliss WG, Bandari S, et al. A comparison between lab-scale and hot-melt-extruder-based anti-inflammatory Ointment Manufacturing. AAPS PharmSciTech. 2020;21(5):200. 32676978 10.1208/s12249-020-01738-5

[137] Thanki K, Papai S, Lokras A, Rose F, Falkenberg E, Franzyk H, et al. Application of a Quality-By-Design Approach to optimise lipid-polymer hybrid nanoparticles loaded with a splice-correction antisense oligonucleotide: Maximising Loading and Intracellular Delivery. Pharm Res. 2019;36(3):37. 30623253 10.1007/s11095-018-2566-3

[138] Thanki K, Zeng X, Justesen S, Tejlmann S, Falkenberg E, Van Driessche E, et al. Engineering of small interfering RNA-loaded lipidoid-poly(DL -lactic-co-glycolic acid) hybrid nanoparticles for highly efficient and safe gene silencing: a quality by design-based approach. Eur J Pharm Biopharm. 2017;120:22–33. 28756280 10.1016/j.ejpb.2017.07.014

[139] The mal ERACGoV. A Research Agenda for Malaria Eradication: vaccines. PLoS Med. 2011;8(1):e1000398. 21311586 10.1371/journal.pmed.1000398 PMC3026701

[140] Timpe C, Stegemann S, Barrett A, Mujumdar S. Challenges and opportunities to include patient-centric product design in industrial medicines development to improve therapeutic goals. Brit J Clin Pharma. 2020;86(10):2020–7. 10.1111/bcp.14388 PMC7495299 32441052

[141] Torregrosa A, Ochoa-Andrade AT, Parente ME, Vidarte A, Guarinoni G, Savio E. Development of an emulgel for the treatment of rosacea using quality by design approach. Drug Dev Ind Pharm. 2020;46(2):296–308. 31944126 10.1080/03639045.2020.1717515

[142] Vetter B, Beran D, Boulle P, Chua A, De La Tour R, Hattingh L, et al. Development of a target product profile for a point-of-care cardiometabolic device. BMC Cardiovasc Disord. 2021;21(1):486. 34627153 10.1186/s12872-021-02298-7 PMC8501932

[143] Villamagna AH, Gore SJ, Lewis JS, Doggett JS. The need for antiviral drugs for pandemic coronaviruses from a Global Health Perspective. Front Med. 2020;7:596587. 10.3389/fmed.2020.596587 PMC7783399 33415116

[144] Vitoria M, Rangaraj A, Ford N, Doherty M. Current and future priorities for the development of optimal HIV drugs. Curr Opin HIV AIDS. 2019;14(2):143–9. 30562177 10.1097/COH.0000000000000527

[145] Vliegenthart ADB, Antoine DJ, Dear JW. Target biomarker profile for the clinical management of Paracetamol overdose. Brit J Clin Pharma. 2015;80(3):351–62. 10.1111/bcp.12699 PMC4574821 26076366

[146] Vora C, Patadia R, Mittal K, Mashru R. Risk based approach for design and optimization of stomach specific delivery of rifampicin. Int J Pharm. 2013;455(1–2):169–81. 23916823 10.1016/j.ijpharm.2013.07.043

[147] Waghule T, Dabholkar N, Gorantla S, Rapalli VK, Saha RN, Singhvi G. Quality by design (QbD) in the formulation and optimization of liquid crystalline nanoparticles (LCNPs): a risk based industrial approach. Biomed Pharmacother. 2021;141:111940. 34328089 10.1016/j.biopha.2021.111940

[148] Walsh J, Masini T, Huttner B, Moja L, Penazzato M, Cappello B. Assessing the appropriateness of formulations on the WHO Model list of essential Medicines for children: Development of a Paediatric Quality Target Product Profile Tool. Pharmaceutics. 2022;14(3):473. 35335850 10.3390/pharmaceutics14030473 PMC8950931

[149] Walsh J, Schaufelberger D, Iurian S, Klein S, Batchelor H, Turner R, et al. Path towards efficient paediatric formulation development based on partnering with clinical pharmacologists and clinicians, a conect4children expert group white paper. Brit J Clin Pharma. 2022;88(12):5034–51. 10.1111/bcp.14989 34265091

[150] Wang Y, Müllertz A, Rantanen J. Structured approach for designing drug-loaded solid products by binder jetting 3D printing. Eur J Pharm Sci. 2022;178:106280. 36041334 10.1016/j.ejps.2022.106280

[151] Wyber R, Boyd BJ, Colquhoun S, Currie BJ, Engel M, Kado J, et al. Preliminary consultation on preferred product characteristics of benzathine penicillin G for secondary prophylaxis of rheumatic fever. Drug Deliv Transl Res. 2016;6(5):572–8. 27465618 10.1007/s13346-016-0313-z

[152] Zhang E, Xie L, Qin P, Lu L, Xu Y, Gao W, et al. Quality by design–based Assessment for Analytical Similarity of Adalimumab Biosimilar HLX03 to Humira^®^. AAPS J. 2020;22(3):69. 32385732 10.1208/s12248-020-00454-z PMC7210234

[153] Zidan A, Ahmed O, Aljaeid B. Nicotinamide polymeric nanoemulsified systems: a quality-by-design case study for a sustained antimicrobial activity. IJN. 2016:1501. 10.2147/IJN.S102945 PMC4835127 27110111

[154] guideline IQRPd-S. International Conference on Harmonisation of Technical Requirements for Registration of Pharmaceuticals for Human Use. 10.1111/j.1365-2125.1994.tb05705.x PMC1364893 8054244

[155] FIND. Target product profiles. https://www.finddx.org/tools-and-resources/rd-and-innovation/target-product-profiles/.

[156] Organization WH. Global status report on the public health response to dementia. 2021.

[157] Organization WH. An R&D blueprint for action to prevent epidemics. Plan Action. 2016.

[158] Organization WH. A blueprint for dementia research. Geneva: World Health Organization; 2022.

[159] Mahant V. Translational medicines ecosystem. J Translational Med. 2020;18(1):158. 10.1186/s12967-020-02325-9 PMC7137407 32252786

[160] Charnaud SCMV, Reeder J, Ross AL. WHO target product profiles to shape global research and development. Bull World Health Organ. 2023;101(5):326–30. 37131943 10.2471/BLT.22.289521 PMC10140686

