# Supplementary material for: Facilitating the use of the target product profile in academic research: a systematic review
Source: J Transl Med. 2024 Jul 29;22:693. doi: 10.1186/s12967-024-05476-1 (PMC11288132; doi:10.1186/s12967-024-05476-1)
Supplement: Supplementary file 1 — Supplementary Material 1 [file 12967_2024_5476_MOESM1_ESM.docx]

Supplemental material of:

**Facilitating the use of the target product profile in academic research: a systematic review**

Aliaa Ibnidris^1,2^, Nektarios Liaskos^1,3^, Ece Eldem,^1^ Angus Gunn^4^, Johannes Streffer^5^, Michael Gold^6^, Mike Rea^7^, Stefan Teipel^1,8^, Alejandra Gardiol^3,9^, Marina Boccardi.^1,8^

^1^ German Center for Neurodegenerative Diseases (DZNE), Rostock-Greifswald site, Rostock, Germany

^2^ Neuroscience Institute, Department of Psychiatry and Mental Health, University of Cape Town, Cape Town, South Africa

^3^ European Infrastructure for Translational Medicine (EATRIS), Amsterdam, The Netherlands

^4^ UCB, Brussels, Belgium

^5^ Reference Center for Biological Markers of Dementia (BIODEM), Department of Biomedical Sciences, University of Antwerp, Antwerp, Belgium

^6^ AriLex Life Sciences LLC

^7^ IDEA Pharma, London, United Kingdom

^8^ Department of Psychosomatic Medicine and Psychotherapy, University of Medicine Rostock, Rostock, Germany

^9^ Queen Mary University of London, London, UK

**Corresponding author**: Marina Boccardi ([marina.boccardi@dzne.de](mailto:marina.boccardi@dzne.de))

DZNE Rostock-Greifswald,

Gehlsheimer Str. 20, 18147, Rostock,

Germany

**Table S1: Example of a TPP for a drug.** Modified to mask the product (XXX) and included with permission of the owning company. Examples of TPPs for diagnostics are easily available online (see, for example: <https://cdn.who.int/media/docs/default-source/in-vitro-diagnostics/calls/tpp-rdt-readers---public-consultation-draft.pdf?sfvrsn=1f78566f_1>, <https://www.who.int/docs/default-source/documents/health-topics/meningitis/in-vitro-diagnostics-test-multi-pathogen-meningitis-tpp.pdf?sfvrsn=dfa859cc_2>). For every aspect addressed by the TPP (“Attribute”, first column), TPPs list a variable number of levels of performance expected for the planned product (columns 2-4). This example reports three possible levels of performance. If the product performs at the lowest level (“Current practice”), it has no or limited little advantage compared to products already available on the market. The highest level corresponds to yet not available features: the product would in this case lead the market. Higher performance levels may include the achievement of lower levels, and provide additional desirable features. Therefore, minimal or basic desirable features may be

replicated in the cells corresponding to higher performance for the same dimension.

| Attribute | Current practice | Minimally acceptable | Ideal |
| --- | --- | --- | --- |
| Indication and Usage | XXX is indicated for the adjunctive treatment of mild to moderate dementia of the Alzheimer’s type who do not carry the APOE e4 allele. An active comparator trial is required. | XXX is indicated for the adjunctive treatment of mild to moderate dementia of the Alzheimer’s type in patients who do not carry the APOE ε4 allele. APOE testing required and provided by sponsor in accordance with local regulations. | XXX is indicated for the adjunctive treatment of mild to moderate dementia of the Alzheimer’s type |
| Clinical Studies | Treatment with XXX resulted in clinically and statistically significant benefits in cognition and functional capacity compared to placebo when used adjunctively. Treatment with XXX was equally effective in mildly and moderately demented patients. The observed responder rates were comparable to that of previously approved symptomatic medications for AD | Treatment with XXX resulted in clinically and statistically significant benefits in cognition and functional capacity compared to placebo when used adjunctively. Treatment with XXX resulted in reduced caregiver stress. Treatment with XXX was equally effective in mildly and moderately demented patients. Responder rates were nominally higher than those of previously approved medications | Treatment with XXX resulted in clinically and statistically significant benefits in cognition and functional capacity compared to placebo when used adjunctively. Treatment with XXX resulted in reduced caregiver stress and reduced resource utilization for patients and caregivers. Treatment with XXX was equally effective in mildly and moderately demented patients. Responder rates were nominally higher and of longer duration than those of previously approved medications. |
| Dosage and Administration | Pharmaceutical grade XXX is is intended for oral administration. Dose adjustment may be needed due to APOE status and BMI | Pharmaceutical grade XXX is intended for oral administration. No adjustments are needed due to gender, BMI, diet or concomitant medications | Pharmaceutical grade XXX is intended for oral administration. No adjustments are needed due to APOE status, gender, BMI, diet or concomitant medications |
| Dosage Forms and Strengths | XXX comes in capsules that contain XX gm of XX and XX gm of excipients. | XXX comes in tablets or capsules that contain XX gm of XX and XX gm of excipients. | XXX comes in tablets or capsules that contain XX gm of XX and XX gm of excipients. administration |
| Contraindication | XXX is contraindicated in patients with suspected inflammatory bowel disease or malabsorption or short-bowel syndrome. XXX is contraindicated in patients with severe liver disease and in patients who have experienced episodes of “X" | XXX is contraindicated in patients with a diagnosis of inflammatory bowel disease (UC, Crohn's Disease), a history of mal-absorption or short-bowel syndrome. | XXX is contraindicated in patients with a confirmed allergy to XXX. |
| Warnings and Precautions | No evidence of clinically relevant changes was obtained with regards to physical examinations, ECGs. Serum XXX needs to be monitored periodically during the initiation of therapy | No evidence of clinically relevant changes was obtained with regards to physical examinations, laboratory values or ECGs. | No evidence of clinically relevant changes was obtained with regards to physical examinations, laboratory values or ECGs. |
| Adverse Reactions | XXX is generally safe and well-tolerated. The most common adverse events observed were related to GI symptoms and were mild, transient and generally did not lead to discontinuation. Adverse events were more common in subjects radomized to XXX than placebo but the difference was less than 5%. The side effect profile of XXX is consistent with the known profile of other compounds in this class. | XXX is generally safe and well-tolerated. The most common adverse events observed were related to GI symptoms and were mild, transient and did not lead to discontinuation. No adverse events occurred with a frequency > 5%. The side effect profile of XXX is consistent with the known adverse event profile of other compounds in this class | XXX is generally safe and well-tolerated. The most common adverse events observed were related to GI symptoms and were mild, transient and did not lead to discontinuation. No adverse event occurred with a frequency > 2%. The side effect profile of XXX is consistent with the known profile of other compounds in this class. |
| Drug Interaction | XXX interact with known CYP2D6 inhibitors requiring potential adjustments to dose. | XXX does not have significant interactions with medications commonly used in patients with Alzheimer's Disease. | XXX does not have significant interactions with medications commonly used in patients with Alzheimer's Disease. |
| Drug Abuse and Dependence | XXX has no potential for abuse or dependence. | XXX has no potential for abuse or dependence. | XXX has no potential for abuse or dependence. |
| Overdosage | X cases of overdoses with XXX were identified during the conduct of phase II and III clinical trials. These overdoses were associated with serious but transient diarrhoea requiring aggressive hydration. No SAEs or deaths were reported in association with any of the overdoses. | X cases of overdoses with XXX were identified during the conduct of phase II and III clinical trials. These overdoses were associated with significant, but transient diarrhoea. No SAEs or deaths were reported in association with any of the overdoses. | X cases of overdoses with XXX were identified during the conduct of phase II and III clinical trials. These overdoses were not associated with any adverse events. No SAEs or deaths were reported in association with any of the overdoses. |
| Non-Clinical Toxicology | No evidence of carcinogenicity. Clinical dose has ~ 3X margin over the NOAEL | No evidence of carcinogenicity. Clinical dose has ~ 10X margin over the NOAEL | No evidence of carcinogenicity. Clinical dose has ~ 20X margin over the NOAEL |

Table S2: Excluded papers after full-text review with reasons for exclusion.

| **Authors** | **Year** | **Reason** |
| --- | --- | --- |
| Adu-Gyamfi et al. (1) | 2021 | No TPP structure |
| Adu-Gyamfi et al. (2) | 2020 | No TPP structure |
| Ahmad et al. (3) | 2019 | No TPP structure |
| Alonzo et al. (4) | 2022 | No TPP structure |
| Armistead et al. (5) | 2014 | No TPP structure |
| Azimi et al. (6) | 2017 | No TPP structure |
| Bachman et al. (7) | 2021 | No TPP structure |
| Balaña-Fouce et al. (8) | 2019 | No TPP structure |
| Bardon et al. (9) | 2020 | No TPP structure |
| Bartsch et al. (10) | 2016 | No TPP structure |
| Basilico et al. (11) | 2017 | No TPP structure |
| Bayaa et al. (12) | 2021 | No TPP structure |
| Beck and Liu (13) | 2019 | No TPP structure |
| Hong-Geller et al. (14) | 2010 | No TPP structure |
| Bhargav et al. (15) | 2021 | No TPP structure |
| Biagini et al. (16) | 2012 | No TPP structure |
| Biter et al. (17) | 2018 | No TPP structure |
| Bolla et al. (18) | 2020 | No TPP structure |
| Bolton et al. (19) | 2011 | No TPP structure |
| Borges et al. (20) | 2021 | No TPP structure |
| Breder et al. (21) | 2017 | No TPP structure |
| Broger et al. (22) | 2017 | No TPP structure |
| Brussee et al. (23) | 2021 | No TPP structure |
| Buckner and Navabi (24) | 2010 | No TPP structure |
| Burrows et al. (25) | 2019 | No TPP structure |
| Callahan et al. (26) | 2020 | No TPP structure |
| Calvet et al. (27) | 2017 | No TPP structure |
| Campbell (28) | 2014 | No TPP structure |
| Campbell and Vallejo (29) | 2015 | No TPP structure |
| Charoo and Ali (30) | 2013 | No TPP structure |
| Chavez et al. (31) | 2015 | No TPP structure |
| Chen et al. (32) | 2020 | No TPP structure |
| Chirmule et al. (33) | 2020 | No TPP structure |
| Cocco et al. (34) | 2021 | No TPP structure |
| Cocco et al. (35) | 2021 | Systematic review |
| Conde et al. (36) | 2021 | No TPP structure |
| Cook et al. (37) | 2014 | No TPP structure |
| Craciunas et al. (38) | 2020 | No TPP structure |
| Csóka et al. (39) | 2018 | No TPP structure |
| Dartois et al. (40) | 2013 | No TPP structure |
| Ding et al. (41) | 2021 | No TPP structure |
| Ditter et al. (42) | 2018 | No TPP structure |
| Dostalek et al. (43) | 2017 | No TPP structure |
| Drain et al. (44) | 2019 | No TPP structure |
| Durbin and Wilder-Smith (45) | 2017 | No TPP structure |
| Dybul et al. (46) | 2021 | No TPP structure |
| El Azab (47) | 2021 | No TPP structure |
| El-Say et al. (48) | 2021 | No TPP structure |
| Emperador et al. (49) | 2019 | No TPP structure |
| Entrican et al. (50) | 2021 | No TPP structure |
| Fasiolo et al. (51) | 2019 | No TPP structure |
| Fernandez-Carballo et al. (52) | 2021 | No TPP structure |
| Francis (53) | 2020 | No TPP structure |
| Funk et al. (54) | 2021 | No TPP structure |
| Georghiou et al. (55) | 2019 | No TPP structure |
| Gerlinger et al. (56) | 2020 | No TPP structure |
| Gomez et al. (57) | 2018 | No TPP structure |
| Goscé et al. (58) | 2019 | No TPP structure |
| Grant et al. (59) | 2021 | No TPP structure |
| Günther et al. (60) | 2022 | No TPP structure |
| Gupta et al. (61) | 2020 | No TPP structure |
| Halpern et al. (62) | 2015 | No TPP structure |
| Ham et al. (63) | 2015 | No TPP structure |
| Ham et al. (64) | 2012 | No TPP structure |
| Ham et al. (65) | 2012 | No TPP structure |
| Hampson et al. (66) | 2022 | No TPP structure |
| Hemingway (67) | 2014 | No TPP structure |
| Henríquez et al. (68) | 2019 | No TPP structure |
| Hogan et al. (69) | 2018 | No TPP structure |
| Hotez et al. (70) | 2016 | No TPP structure |
| Jackson et al. (71) | 2019 | No TPP structure |
| Jacobs et al. (72) | 2020 | No TPP structure |
| Januskaite et al. (73) | 2020 | No TPP structure |
| Kapoor et al. (74) | 2021 | No TPP structure |
| Karlen et al. (75) | 2014 | No TPP structure |
| Karp et al. (76) | 2015 | No TPP structure |
| Kauss et al. (77) | 2013 | No TPP structure |
| Keiser and Utzinger (78) | 2012 | No TPP structure |
| Khanolkar et al. (79) | 2017 | No TPP structure |
| Khurana et al. (80) | 2020 | No TPP structure |
| Kik et al. (81) | 2018 | No TPP structure |
| Killeen et al. (82) | 2011 | No TPP structure |
| Kondo and Sako (83) | 2015 | Publication not in English (Japanese) |
| Kotraiah et al. (84) | 2020 | No TPP structure |
| Kruchten et al. (85) | 2014 | No TPP structure |
| Kumar et al. (86) | 2021 | No TPP structure |
| Kumari et al. (87) | 2019 | No TPP structure |
| Kumari et al. (88) | 2022 | No TPP structure |
| Lafeber et al. (89) | 2022 | No TPP structure |
| Lee and McGlone (90) | 2010 | No TPP structure |
| Lingg et al. (91) | 2012 | No TPP structure |
| Liu et al. (92) | 2020 | No TPP structure |
| Llibre et al. (93) | 2018 | No TPP structure |
| Love et al. (94) | 2017 | No TPP structure |
| Luciani et al. (95) | 2015 | No TPP structure |
| Lujan et al. (96) | 2019 | No TPP structure |
| Lukashevich et al. (97) | 2019 | No TPP structure |
| Lyu et al. (98) | 2021 | No TPP structure |
| Mabille et al. (99) | 2022 | No TPP structure |
| MacLean et al. (100) | 2019 | No TPP structure |
| Makepeace et al. (101) | 2009 | No TPP structure |
| Malebo et al. (102) | 2020 | No TPP structure |
| Manga et al. (103) | 2016 | No TPP structure |
| Marcantonio et al. (104) | 2022 | No TPP structure |
| Marie et al. (105) | 2022 | No TPP structure |
| Marshall et al. (106) | 2019 | No TPP structure |
| Martín-Escolano et al. (107) | 2022 | No TPP structure |
| Martín-Escolano et al. (108) | 2021 | No TPP structure |
| Martinez-Marquez et al. (109) | 2020 | No TPP structure |
| Mduluza and Mutapi (110) | 2017 | No TPP structure |
| Mendelsohn et al. (111) | 2021 | No TPP structure |
| Meyer et al. (112) | 2020 | No TPP structure |
| Michel et al. (113) | 2020 | No TPP structure |
| Milner et al. (114) | 2010 | No TPP structure |
| Mordt et al. (115) | 2022 | No TPP structure |
| Moreira et al. (116) | 2021 | No TPP structure |
| Mukadi-Bamuleka et al. (117) | 2022 | No TPP structure |
| Mulenga et al. (118) | 2020 | No TPP structure |
| Muller et al. (119) | 2014 | No TPP structure |
| Murphy et al. (120) | 2017 | No TPP structure |
| Mutavhatsindi et al. (121) | 2021 | No TPP structure |
| Muzembo et al. (122) | 2022 | No TPP structure |
| Nukala et al. (123) | 2019 | No TPP structure |
| Ogoma et al. (124) | 2012 | No TPP structure |
| Olías-Molero et al. (125) | 2021 | No TPP structure |
| Olliaro et al. (126) | 2011 | No TPP structure |
| Orlandini et al. (127) | 2013 | No TPP structure |
| Page et al. (128) | 2016 | No TPP structure |
| Pan-Ngum et al. (129) | 2017 | No TPP structure |
| Pantelias et al. (130) | 2022 | No TPP structure |
| Paolantonacci et al. (131) | 2018 | No TPP structure |
| Patel and Rohan (132) | 2017 | No TPP structure |
| Peck et al. (From Cocco’s (35) references; full citation unavaiable) | 2012 | Full-text unavailable, online page does not exist |
| Penn-Nicholson et al.(133) | 2022 | No TPP structure |
| Penn-Nicholson et al. (134) | 2019 | No TPP structure |
| Pham et al. (135) | 2020 | No TPP structure |
| Pham et al. (136) | 2019 | No TPP structure |
| Phillips et al. (137) | 2016 | No TPP structure |
| Pidathala et al. (138) | 2012 | No TPP structure |
| Politis et al. (139) | 2017 | No TPP structure |
| Polli et al. (140) | 2012 | No TPP structure |
| Pulkstenis et al. (141) | 2017 | No TPP structure |
| Qin et al. (142) | 2021 | No TPP structure |
| R &D Alfimepraze (143) | 2008 | No TPP structure |
| Rathod et al. (144) | 2020 | No TPP structure |
| Raw et al. (145) | 2011 | No TPP structure |
| Rawal et al. (146) | 2019 | No TPP structure |
| Rayfield et al. (147) | 2017 | No TPP structure |
| Roudier et al. (148) | 2015 | No TPP structure |
| Salave et al. (149) | 2022 | No TPP structure |
| Sam et al. (150) | 2012 | No TPP structure |
| Sammut-Powell et al. (151) | 2022 | No TPP structure |
| Santos et al. (152) | 2021 | No TPP structure |
| Schiefer et al. (153) | 2020 | No TPP structure |
| Schlesinger et al. (154) | 2018 | No TPP structure |
| Schneider et al. (155) | 2020 | No TPP structure |
| Selen et al. (156) | 2014 | No TPP structure |
| Shah et al. (157) | 2020 | No TPP structure |
| Shoukat et al. (158) | 2018 | No TPP structure |
| Silva et al. (159) | 2018 | No TPP structure |
| Simpson et al. (160) | 2020 | No TPP structure |
| Singhai et al. (161) | 2021 | No TPP structure |
| Soeiro (162) | 2022 | No TPP structure |
| Specht et al. (163) | 2018 | No TPP structure |
| Spooner et al. (164) | 2022 | No TPP structure |
| Stegemann (165) | 2018 | No TPP structure |
| Stegemann et al. (166) | 2022 | No TPP structure |
| Stevenson et al. (167) | 2021 | No TPP structure |
| Subramanian et al. (168) | 2022 | No TPP structure |
| Sulaiman et al. (169) | 2019 | No TPP structure |
| Sumner et al. (170) | 2021 | No TPP structure |
| Sutherland et al. (171) | 2022 | No TPP structure |
| Swain et al. (172) | 2019 | No TPP structure |
| Tan et al. (173) | 2016 | No TPP structure |
| Tari (174) | 2012 | No TPP structure |
| Teng et al. (175) | 2018 | No TPP structure |
| Thakkar et al. (176) | 2020 | No TPP structure |
| Than and Titapiwatanakun (177) | 2021 | No TPP structure |
| Tietje et al. (178) | 2014 | No TPP structure |
| Tolley et al. (179) | 2014 | No TPP structure |
| Troiano et al. (180) | 2016 | No TPP structure |
| Turner et al. (181) | 2020 | No TPP structure |
| Turner et al. (182) | 2016 | No TPP structure |
| Tyndall et al. (183) | 2017 | No TPP structure |
| Vannice et al. (184) | 2019 | No TPP structure |
| Visser et al. (185) | 2015 | No TPP structure |
| Wang et al. (186) | 2022 | No TPP structure |
| Warman et al. (187) | 2013 | No TPP structure |
| Warsinske et al. (188) | 2018 | No TPP structure |
| Watson and Nosal (189) | 2019 | No TPP structure |
| Wen and Jawa (190) | 2021 | No TPP structure |
| White and Nosten (191) | 2021 | No TPP structure |
| Winter et al. (192) | 2019 | No TPP structure |
| Wyatt et al. (193) | 2011 | No TPP structure |
| Xu et al. (194) | 2020 | No TPP structure |
| Yang et al. (195) | 2019 | No TPP structure |
| Yao et al. (196) | 2022 | No TPP structure |
| Yost et al. (197) | 2022 | No TPP structure |
| Yu et al. (198) | 2014 | No TPP structure |

Table S3: List of citations for the extracted data items “content”, “product type”,

| **Data item** | **Values** | **Citations** |
| --- | --- | --- |
| **Content** | Developed a new TPP | (199–258,258–325) |
|  | Revised a predefined TPP | (326–330) |
|  | Described a pre-existing TPP | (331–335) |
| **Product type** | Therapeutics | (211–213,215,217,218,220–223,225–227,229–231,234,240,246,247,249,251–254,258,259,266–268,270,272,273,277,280,282,285,292,295,296,299,301,305–308,312,315,316,319–321,323,324,328,334,335) |
|  | Diagnostics | (199–209,218,233,235,238,239,241,243,244,262,274,283,294,295,317,326,327,331,333) |
|  | Vaccines | (242,256,264,271,278,298,311,329) |
|  | Medical devices | (293,314,330) |
|  | Other products (e.g., apps, technology, etc.) | (21,25,27–29,37,41,45,49,50,58,63,65,70,75,76,78,80,84,91,92,96,98,102,104–109,115,118,120,122,123,128,129,132,137,141,144) |
| **Disease category** | Infectious disease | (7–20,23,31,33–35,37,46,48,51,52,54–59,61–63,66,68,69,71,72,76–79,81,83,85–88,90,94,95,101,106,112–114,116, 120,122,123,130,134,135,137,142) |
|  | Non-infectious disease | (210,211,213–217,226,231,234,236,237,240,247,249,250,252,260,265,267,269,273,277,279,284,289,297,299,305,309,310,313,314,317,330) |
|  | Disease category not specified | (219,223,225,227–230,232,255,258,259,275,276,280–282,285–287,291–293,300,301,306–308,312,319–322,324,325,332,335,336) |
| **Affiliation** | Academia | (208–218,220,224–226,228–232,236,237,240,241,243,245,247–251,255,256,259–261,263–265,267,269,273,276,277,279–282,284–290,293,299–302,305,306,308–310,315,317–319,323,325,327,328,331,332,334,336) |
|  | Non-academia | (219,221,222,252,258,266,270,311,275,257,335,314,316,324,205,203) |
|  | Collaborators from academic and non-academic affiliations | (199–202,204,206,207,223,227,233–235,238,239,242,244,246,253,254,262,268,271,272,274,278,283,291,292,294–298,303,304,307,312,313,320–322,329,330,333) |
| **Thresholds** | One threshold | (201,203,205,206,208,211,213,215,216,218,228–232,234,236,237,240,242,244,245,247–249,253,255,256,258,260,261,263–265,267,269,271,273,275–277,279–282,284–290,292,293,295–297,299–302,305–310,312,313,316,318–322,324,325,328,331,334) |
|  | Two thresholds | (199,200,202,204,207,209,210,212,214,221–224,233,235,238,239,241,243,246,254,257,262,266,268,270,274,283,291,298,303,304,311,314,315,317,323,326,330,333) |
|  | Three thresholds | (259,272,278) |
|  | Not reported | (217,219,220,225–227,250–252,294,327,329,332,335,336) |
| **Categories** | Papers categorising TPP features | (200–202,204,207,209,218,233,235,238,239,241,243,257,262,266,274,283,291,293,314) |
|  | Papers reporting the category “Scope” (most reported category) | (200–202,204,207,209,218,233,235,238,239,241,243,257,262,274,283,291,314) |

Table S4: Distribution of target product profiles (TPPs) by *disease category and* specific disease. Abbreviations: TB: Tuberculosis; HIV: Human Immunodeficiency Virus; COVID-19: Coronavirus Disease 19; COPD: Chronic Obstructive Pulmonary Disease; STDs: Sexually Transmitted Diseases

| **Disease category** | **Specific disease** | **Number of publications** |
| --- | --- | --- |
| **Infectious** | Malaria | 12 |
|  | TB | 8 |
|  | HIV | 6 |
|  | COVID-19 | 4 |
|  | Dengue fever | 3 |
|  | Schistosomiasis | 3 |
|  | Chagas disease | 3 |
|  | Leishmaniasis | 3 |
|  | STDs | 2 |
|  | Cryptosporidiosis | 2 |
|  | Zika virus | 2 |
|  | Rift Valley fever | 1 |
|  | Fungal infection | 1 |
|  | Soil-transmitted helminths | 1 |
|  | Poliomyelitis | 1 |
|  | Sepsis | 1 |
|  | Bacterial and non-bacterial infections | 1 |
|  | HIV and STDs | 1 |
|  | Hepatitis C | 1 |
|  | Taenia solium | 1 |
|  | Shigellosis | 1 |
|  | Lower respiratory infections | 1 |
|  | Viral infections | 1 |
|  | Typhoid fever | 1 |
|  | Rheumatic fever | 1 |
|  | Lassa virus | 1 |
|  | Neglected Tropical Diseases | 1 |
|  | NA | 1 |
| Total |  | **65** |
| **Non-infectious** | NA | 3 |
|  | Psychiatric (general) | 3 |
|  | Hypertension | 2 |
|  | Schizophrenia | 2 |
|  | Genetic disorders | 2 |
|  | Cholesterolemia | 1 |
|  | Skin disease | 1 |
|  | Cancer | 1 |
|  | Osteoarthritis | 1 |
|  | COPD | 1 |
|  | Skin cancer | 1 |
|  | Cardiac disease (heart failure) | 1 |
|  | Monoclonal antibodies production | 1 |
|  | Alzheimer's disease | **1** |
|  | Malnutrition | 1 |
|  | Dermatological disease (Rosacea) | 1 |
|  | Leukaemia | 1 |
|  | Chronic respiratory disease | 1 |
|  | Skin permeation chemicals | 1 |
|  | Cardiometabolic disease | 1 |
|  | Hyperlipidaemia | 1 |
|  | Cancer therapy delivery systems | 1 |
|  | Wound pain management | 1 |
|  | Binge eating disorder | 1 |
|  | Gastrointestinal disease | 1 |
|  | Acute liver injury | 1 |
|  | Gastric ulcer | 1 |
|  | Diabetes | 1 |
| Total |  | **35** |
| **Other** | NA | 33 |
|  | Skin disease | 1 |
|  | Eye conditions | 1 |
|  | Skin conditions | 1 |
|  | Respiratory disease | 1 |
| Total |  | **37** |
| **NA** | NA | 1 |
| Total |  | **1** |
| Grand Total |  | **138** |

Figure S1: Number of TPP features across 29 TPPs for diagnostic tests.


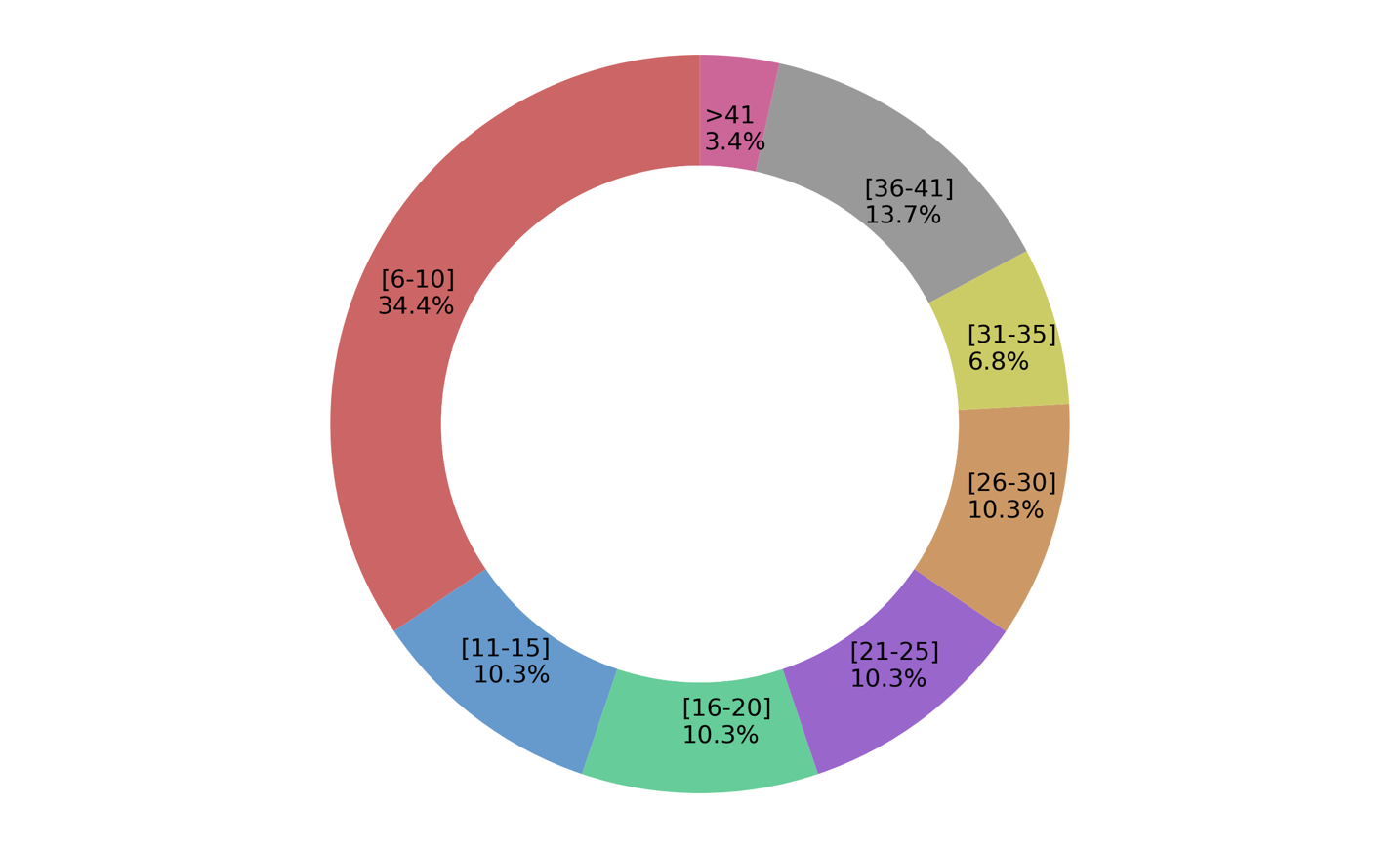


Figure S2: Number of TPP features across 57 TPPs for therapeutics.


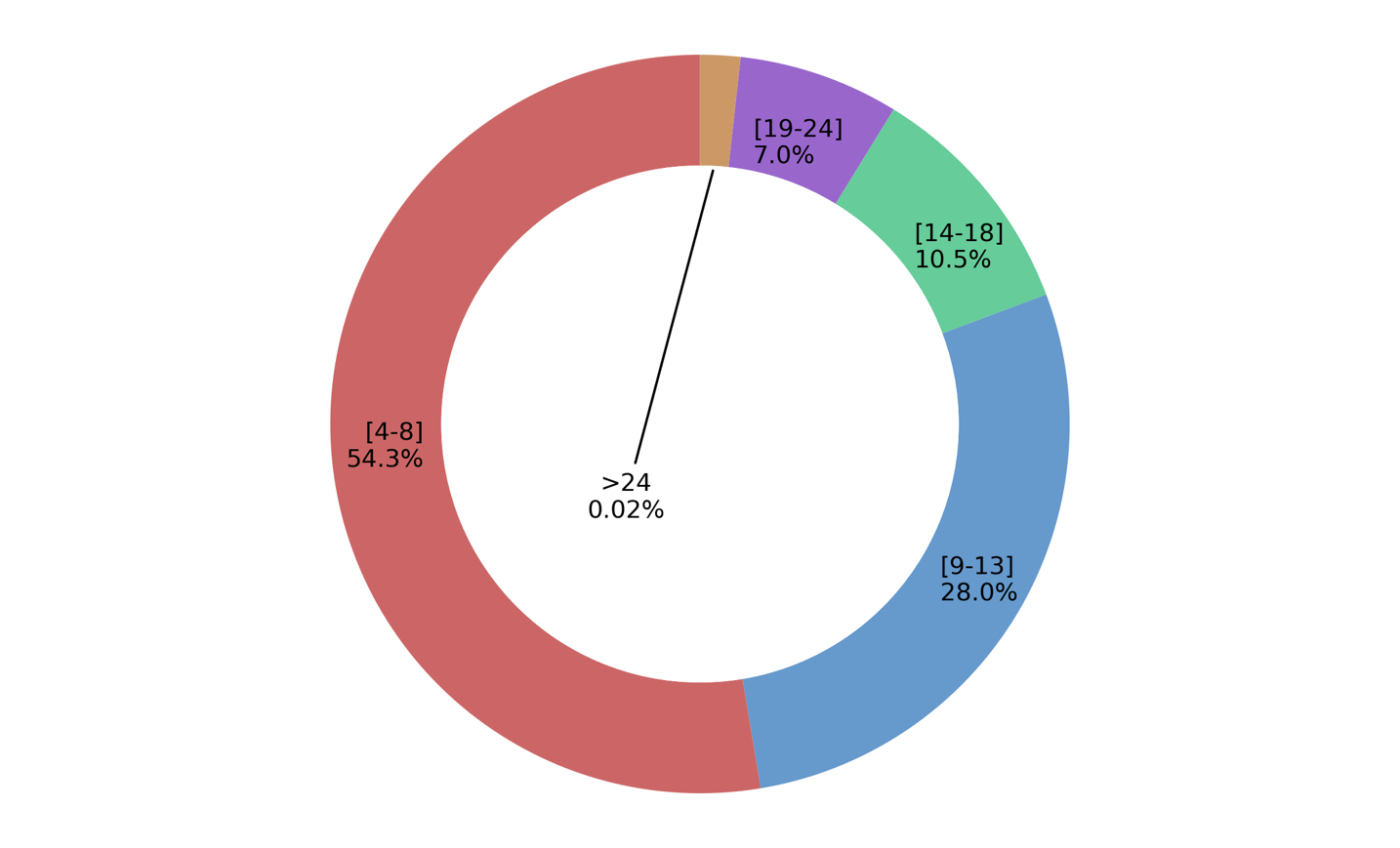


Figure S3: Number of TPP features across TPPs for other products.


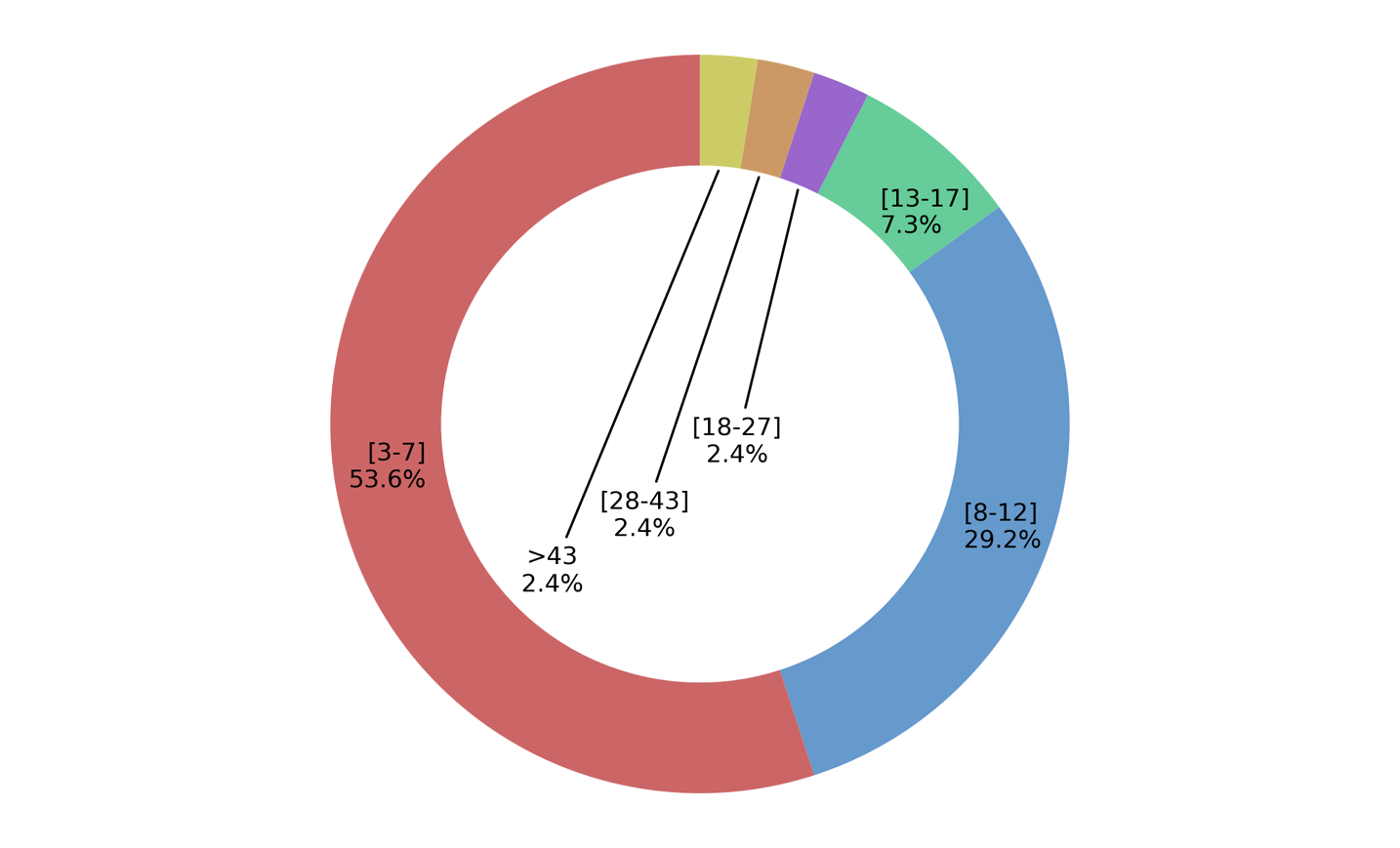


Table S5: Comprehensive list of TPP features for all product types. The frequency of each feature is reported between brackets.

| **Diagnostics** | **Drugs** | **Vaccines** | **Medical devices** | **Other products** |
| --- | --- | --- | --- | --- |
| Testing Sensitivity and specificity (n=27) | Route of administration (n=32) | Indication (n=6) | Indication (n=2) | Route of administration (n=28) |
| Indication (n=23) | Stability/shelf life (n=29) | Target population (n=4) | Cost of test/product/reimbursement (n=2) | Stability/shelf life (n=26) |
| Target population (n=23) | Dosage form (n=23) | Repeatability, stability (n=4) | Data output (n=2) | Dosage strength (n=25) |
| Target user (n=22) | Dosage strength (n=23) | Storage conditions and shelf life (n=3) | Accuracy (n=2) | Dosage form (n=23) |
| Sample type (n=20) | Indication (n=18) | Dose regimen and amount (n=3) | Target user (n=1) | Container closure system (n=13) |
| Time to result (n=19) | Dose regimen and amount (n=18) | Route of administration (n=3) | Setting (n=1) | Pharmacokinetics (n=11) |
| Cost of test/product/reimbursement (n=18) | Safety (for individuals) (n=18) | Duration of Protection (n=3) | Stability/shelf life (n=1) | Dosage form design (n=8) |
| Specimen preparation (n=17) | Cost of test/product/reimbursement (n=16) | Efficacy (n=3) | Instrument Connectivity and power (n=1) | Assay (n=7) |
| Level of implementation in the healthcare system (n=15) | Target population (n=14) | Cost of test/product/reimbursement (n=2) | Service maintenance and calibration requirements (n=1) | Polydispersity index (PDI) (n=7) |
| Repeatability, limit of detection (LOD), stability (n=15) | Storage conditions and shelf life (n=12) | Product presentation (n=2) | Reagent kit transport stability/cold chain requirements (n=1) | Indication (n=6) |
| Instrument Connectivity and power (n=15) | Container closure system (n=12) | Safety (for individuals) (n=2) | Presence of internal controls (n=1) | Storage conditions and shelf life (n=6) |
| Internal quality control (n=15) | Pharmacokinetics (n=181) | Pregnancy (n=2) | Data export (e.g. for quality assurance purposes) (n=1) | Purity/impurities (n=5) |
| Service maintenance and calibration requirements (n=14) | Clinical efficacy (n=10) | Nr. Doses per Series (n=2) | Throughput capacity (n=1) | Physical properties/attributes/description (n=5) |
| Setting (n=13) | (Drug-drug) Interactions (n=9) | Series (years of age) (n=2) | Operating temperature (n=1) | Dissolution (n=5) |
| Waste disposal requirements (n=12) | Purity/impurities (n=9) | Stability/shelf life (n=1) | Instrument size (portability) (n=1) | Dosage type (n=5) |
| Training and education needs (n=12) | Dissolution (n=8) | Waste disposal requirements (n=1) | Waste disposal requirements (n=1) | Packaging (n=5) |
| Required equipment and instrumentation (n=11) | Formulations (n=7) | Training and education needs (n=1) | Sample type (n=1) | Drug product quality attributes (n=5) |
| Throughput capacity (n=10) | Packaging (n=7) | Ease of test performance/ease of administration (n=1) | Sample volume (n=1) | Dosage shape/appearance (n=5) |
| Operating temperature (n=10) | Dosage shape/appearance (n=7) | Use-case scenarios (n=1) | Training and education needs (n=1) | Identification (n=5) |
| Sample volume (n=10) | Efficacy (n=6) | Target countries (n=1) | Biosafety (n=1) | (Alternative) device/methods of administration (n=4) |
| Reagen kit transport stability/cold chain requirements (n=9) | Mechanism of action (n=6) | Immunogenicity (n=1) | Regulatory requirements (n=1) | Particle size (n=4) |
| Biosafety (n=8) | Physical properties/attributes/description (n=6) | (Drug-drug) Interactions (n=1) | Storage conditions and shelf life (n=1) | Microbial limits/content (n=4) |
| Analytical sensitivity and specificity (n=7) | Assay (n=6) | Mass median aerodynamic diameter (MMD) (n=1) | Operation conditions (n=1) | Zeta potential (n=4) |
| Reagent kit storage and stability (n=6) | pH (n=6) | Liposome size (n=1) | Route of administration (n=1) | pH (n=4) |
| Data export (e.g. for quality assurance purposes) (n=6) | Content uniformity (n=6) | Yield (%) (n=1) | (Detection) range (n=1) | Drug release (%) (n=4) |
| Cost of instrumentation (n=6) | Contraindications (n=5) | Relative moisture content (%) (n=1) | Precision (n=1) | Target population (n=3) |
| Type of analysis (qualitative, quantitative) (n=6) | Dosage form design (n=5) | Age for first dose (n=1) | Humidity (n=1) | Dose regimen and amount (n=3) |
| (test) Format (n=6) | Dosage administration (n. per day) (n=5) | Duration of dosing (n=1) | Mass (n=1) | Contraindications (n=3) |
| Additional supplies (not included in the kit) (n=6) | Particle size (n=5) | Concomitant use (n=1) | Data collection (n=1) | Size (n=3) |
| Data/result interpretation (n=6) | Microbial limits/content (n=5) | Formulations (n=1) | Durability (n=1) | PK target safety (n=3) |
| Reference standard (n=6) | Rheological properties: Viscosity/stiffness (n=5) | Product registration and WHO prequalification (n=1) | Description of product (n=1) | Residual solvent (n=3) |
| Reading system (n=5) | Description of product (n=4) | Transmission blocking (n=1) | Device design (n=1) | Weight (n=3) |
| Ease of test performance/ease of administration (n=5) | Drug product quality attributes (n=4) | Virulence (n=1) | Device weight (n=1) | Encapsulation efficiency (n=3) |
| Sample collection (n=5) | Susceptibility to loss of efficacy due to acquired resistance (n=4) | Ease of production (n=1) | Patient identification capability (n=1) | z-average (nm) (n=3) |
| Storage conditions and shelf life (n=5) | Identification (n=4) | Safe to produce (n=1) | Result output (n=1) | Loading efficiency (n=3) |
| Water supply/requirements (n=5) | Zeta potential (n=4) | Coadministration (n=1) | Memory (n=1) | Solubility (n=3) |
| User interface (n=4) | Side effects/adverse reactions (n=4) | Coverage against other strains of virus (n=1) | Manufacturing standards (n=1) | Cost of test/product/reimbursement (n=2) |
| Treatment monitoring capacity (n=4) | Rate of onset of action (n=3) |  | Test cartridge: Analytes/test menu (n=1) | Dosage administration (n. per day) (n=2) |
| Number of steps by operator (n=4) | (Alternative) device/methods of administration (n=3) |  | Description of test cartridge/strip (n=1) | Percentage friability (n=2) |
| Reagent integration into consumable device (n=3) | Polydispersity index (PDI) (n=3) |  | Multiplexing of simultaneous tests (n=1) | Drug loading (n=2) |
| Instrument size (portability) (n=3) | Homogeneity and tube uniformity (n=3) |  | Additional third-party consumables (n=1) | In vitro release test/profile (n=2) |
| Detection of mixed pathogens (n=3) | In vitro release test/profile (n=3) |  | Interfering substances (n=1) | Content uniformity (n=2) |
| Regulatory requirements (n=3) | Encapsulation efficiency (n=3) |  | Standardization and traceability (n=1) | Site of action (n=2) |
| Multiplexing (n=3) | Release profile (n=3) |  | Test result (quantitative/qualitative) (n=1) | Cell viability (n=2) |
| Reagent/test preparation (n=3) | Drug release (%) (n=3) |  | Distribution territory (n=1) | Target user (n=1) |
| Data output (n=3) | Reagen kit transport stability/cold chain requirements (n=2) |  | Packaging (n=1) | Level of implementation in the healthcare system (n=1) |
| Target molecule (n=3) | Tolerability (n=2) |  |  | Instrument Connectivity and power (n=1) |
| Reproducibility near clinical threshold (n=3) | Duration of dosing (n=2) |  |  | Internal quality control (n=1) |
| Result output (n=3) | Product quality (n=2) |  |  | Training and education needs (n=1) |
| Data capture (n=3) | PK target efficacy (n=2) |  |  | Data output (n=1) |
| Testing outcome (n=2) | Percentage friability (n=2) |  |  | Safety (for individuals) (n=1) |
| Stability/shelf life (n=2) | Drug content (n=2) |  |  | (Drug-drug) Interactions (n=1) |
| External control (n=2) | Product registration and WHO prequalification (n=2) |  |  | Yield (%) (n=1) |
| Ideal diagnostic marker (n=2) | Disintegration (n=2) |  |  | Mechanism of action (n=1) |
| Presence of internal controls (n=2) | Residual solvent (n=2) |  |  | Posology (n=1) |
| Ability to test for other diseases/multiuse platform (n=2) | Weight (n=2) |  |  | Therapeutic (antimalarial) effects (n=1) |
| Operating altitude (n=2) | Identity (n=2) |  |  | Clinical efficacy (n=1) |
| Reagent reconstitution (n=2) | Isotonicity (n=2) |  |  | Source DNA (n=1) |
| In use stability (n=2) | Proportional reduction in parasite load (n=2) |  |  | Complexity (n=1) |
| Language (n=2) | (Relapse) prevention (n=2) |  |  | Drive target locus (n=1) |
| Pathogen identification (n=2) | Product container type/size (n=2) |  |  | Male competitiveness (n=1) |
| Interfering substance (n=2) | Oil globule size (n=2) |  |  | Female fecundity (n=1) |
| Data analysis requirements (n=2) | Preservatives content (n=2) |  |  | Strain maintenance (n=1) |
| Kit configuration (n=2) | Administration/ Concurrence with labelling (n=2) |  |  | Drive inheritance (n=1) |
| Process control (n=2) | Drug delivery system/type (n=2) |  |  | Efficacy (percent population carrying gene) (n=1) |
| Hands on time (n=2) | Osmotic pressure/osmolarity (n=2) |  |  | Time to full introduction (n=1) |
| PPVs and NPVs (n=2) | Medication size/shape (n=2) |  |  | Impact of resistant drive targets (n=1) |
| Accuracy (n=2) | Potency (n=2) |  |  | Off-target drive effects (n=1) |
| Packaging (n=2) | Human abuse/dependence (n=2) |  |  | Nontarget drive effects (n=1) |
| Data storage (n=2) | Site of action (n=2) |  |  | Bioequivalence (n=1) |
| Specimen location of preparation (n=1) | Excipients compendial (n=2) |  |  | Aerodynamic particles properties (n=1) |
| Result determination (n=1) | Water content (n=2) |  |  | Alternative available dosage form (n=1) |
| Diagnostic data reported (n=1) | Molecular weight of Active Pharmaceutical Ingredient (API) (n=2) |  |  | Cumulative drug release (n=1) |
| Patient/case data reported (n=1) | Log P (n=2) |  |  | In vivo performance (n=1) |
| Contextual data reported (n=1) | Rheological properties: Yield stress (n=2) |  |  | Pharmacopoeial compliance (n=1) |
| Availability of ideal diagnostic marker (n=1) | Warnings and precautions (n=2) |  |  | Uniformity of dosage units (n=1) |
| Cost of diagnosis per sample (n=1) | Dose flexibility (n=2) |  |  | Manufacturability (n=1) |
| Instrument & test supply reliability (n=1) | Patient acceptability (n=2) |  |  | Identification of telmisartan (n=1) |
| Test robustness requirements (n=1) | Dose preparation/manipulation (n=2) |  |  | Appearance and colour of pellets (n=1) |
| Supply: channels to marker (n=1) | Setting (n=1) |  |  | Pellet size (n=1) |
| Capacity for drug susceptibility testing (n=1) | Overdose (n=1) |  |  | Pellet shape (n=1) |
| Technology/platform type (n=1) | Target countries (n=1) |  |  | Surface texture (n=1) |
| Lighting of the operating environment (n=1) | Pregnancy (n=1) |  |  | Flowability and compressibility (n=1) |
| Geographic working range (n=1) | Mass median aerodynamic diameter (MMD) (n=1) |  |  | Drug content (n=1) |
| Type of test (n=1) | Yield (%) (n=1) |  |  | Algorithm access (n=1) |
| Speed (n=1) | Relative moisture content (%) (n=1) |  |  | Algorithm content (n=1) |
| Number of samples (n=1) | Mass (n=1) |  |  | Algorithm treatment recommendations (n=1) |
| Timing of sampling (of the first posttreatment sample) (n=1) | Distribution territory (n=1) |  |  | Compatible POC/test tools (n=1) |
| Other (n=1) | Posology (n=1) |  |  | Regulated toolkit components (n=1) |
| Scale of manufacture (n=1) | Therapeutic (antimalarial) effects (n=1) |  |  | Compatible devices (n=1) |
| Delayed entry (n=1) | Use in patients with reduced G6PD activity (n=1) |  |  | Compatible operating systems (n=1) |
| Assay design (n=1) | Use in infants/children (n=1) |  |  | Content transparency (n=1) |
| Daily throughput (n=1) | Dosage type (n=1) |  |  | Algorithm validation (n=1) |
| Walkaway operation (n=1) | Bioequivalence (n=1) |  |  | Machine learning (n=1) |
| Software (n=1) | PK target safety (n=1) |  |  | Data input (n=1) |
| Assay type (n=1) | Manufacturability (n=1) |  |  | Disease likelihood (n=1) |
| Operation conditions (n=1) | Flowability and compressibility (n=1) |  |  | System validation (n=1) |
| Shipping conditions (n=1) | Dose Range (n=1) |  |  | System access (public API) (n=1) |
| Duration of valid result (n=1) | Suitable pharmaceutical properties for intended market product (n=1) |  |  | Context configuration (n=1) |
| Cross-reactivity (n=1) | Bacteriological efficacy (n=1) |  |  | Customisation (n=1) |
| Medical decision to be influenced (n=1) | Ex vivo permeation (n=1) |  |  | User access rights (n=1) |
| Requirement for precise volume (sample / reagent) (n=1) | Drug entrapment efficiency (n=1) |  |  | Expert support (n=1) |
| Test requirements (n=1) | Infusion volume (deliverable) (n=1) |  |  | Patient management recommendation (n=1) |
| Cost of manufacturing single use device (n=1) | Concentration after reconstitution (n=1) |  |  | Navigation (n=1) |
| Competitive landscape (n=1) | Reconstitution time (n=1) |  |  | Workflow requirements to enable time delayed POC data input (n=1) |
| Commercial channels (n=1) | Reconstitution (n=1) |  |  | Task management (n=1) |
| Region(s) of commercialisation (n=1) | (Particle) aggregation (n=1) |  |  | Follow-up (n=1) |
| Market Segmentation (n=1) | Process performance (n=1) |  |  | System malfunction protection (n=1) |
| Taxonomic diagnosis (n=1) | Parasite-free (day 7) (n=1) |  |  | Scalability (n=1) |
| Rapidly assayed (n=1) | Transmission blocking (n=1) |  |  | Updates and versioning (n=1) |
| Feasibility of assay (n=1) | Bioavailability/food effect (n=1) |  |  | Data capture (n=1) |
| Invasiveness (n=1) | Cells per product unit (n=1) |  |  | Data validation |
| Conserved (translational) across in vitro models, in vivo models, and humans (n=1) | Product unit per dose (n=1) |  |  | Data ownership (n=1) |
| Time after overdose at which it is able to predict the onset of organ injury (n=1) | Cell density (n=1) |  |  | Data storage (n=1) |
| Signal to noise (n=1) | Reserve stock (n=1) |  |  | Data recovery |
| Quantitative relationship with disease severity (n=1) | Cryopreserved or fresh (n=1) |  |  | Data flow (n=1) |
| Distinguish benign and clinically relevant increase (n=1) | Phase I–III specific lot size (n=1) |  |  | Data reporting (n=1) |
| Mediator of organ injury (n=1) | Commercial lot size (n=1) |  |  | Data provenance (n=1) |
| Product presentation (n=1) | Market size (n=1) |  |  | Data dictionary (n=1) |
| Target countries (n=1) | Cell stability (n=1) |  |  | Data security and privacy (n=1) |
| Funding/IP global issues (n=1) | Rheological behaviour (n=1) |  |  | Dose Range (n=1) |
| Safety (for individuals) (n=1) | Mucoadhesion time (n=1) |  |  | Palatability (n=1) |
| (Detection) range (n=1) | Lipid-polymer hybrid nanoparticles (LPNs) size ratio (n=1) |  |  | Additional information (n=1) |
| Test cartridge: Analytes/test menu (n=1) | Bioadhesion/retention (n=1) |  |  | Number of compounds in the regimen (n=1) |
| Test result (quantitative/qualitative) (n=1) | Spreading (n=1) |  |  | Availability of DST for the index case (if known) (n=1) |
| Compatible POC/test tools v | Medication colour (n=1) |  |  | Product presentation (n=1) |
| In-app help (n=1) | Applicator (n=1) |  |  | Product registration and WHO prequalification (n=1) |
| Compatible devices (n=1) | Rheology (n=1) |  |  | Disintegration (n=1) |
| Data input (n=1) | Odour (n=1) |  |  | Tensile strength (n=1) |
| User access rights (n=1) | Unit dose (n=1) |  |  | Drug entrapment efficiency (n=1) |
| Service & support response time (n=1) | Visual defects (n=1) |  |  | (Particle) aggregation (n=1) |
| Data ownership (n=1) | Pharmaceutical ingredient (%) (n=1) |  |  | Drug delivery system/type (n=1) |
| Data flow (n=1) | Crushing resistance (n=1) |  |  | In vivo absorption (n=1) |
| Data exchange standards (n=1) | Kinetic drug release (n=1) |  |  | Osmotic pressure/osmolarity (n=1) |
| Data security and privacy (n=1) | Floating lag time (n=1) |  |  | Lysophospholipid (n=1) |
| Handling of intermittent connections (n=1) | Symptoms reduction (n=1) |  |  | F0 (n=1) |
| Product registration and WHO prequalification (n=1) | Cognitive restraint (n=1) |  |  | Odour (n=1) |
| Marketing material allowed (n=1) | Restoration of healthy eating patterns (n=1) |  |  | Release profile (n=1) |
| Product container type/size (n=1) | Reduction of body weight (n=1) |  |  | Floating lag time (n=1) |
| Administration/ Concurrence with labelling (n=1) | Weight loss effect (n=1) |  |  | Freedom to operate (n=1) |
| Need for monitoring (n=1) | Increasing satiety (n=1) |  |  | Gene silencing efficiency (n=1) |
| Polyvalency (n=1) | Pharmacological tolerance (n=1) |  |  | Toxicity (n=1) |
| Lancet (n=1) | Psychological/physical dependence (n=1) |  |  | Physical state (n=1) |
| Transfer device (n=1) | Controlled drug (n=1) |  |  | Degradation products (n=1) |
|  | Microbiologic efficacy (n=1) |  |  | Water content (n=1) |
|  | Drug concentration (n=1) |  |  | (Intermediate step) critical features/quality attributes (n=1) |
|  | Dilution (n=1) |  |  | Rheological properties: Viscosity/stiffness (n=1) |
|  | Freedom to operate (n=1) |  |  | Product/pharmaceutical form (n=1) |
|  | Intellectual property (n=1) |  |  | Fibre diameter (n=1) |
|  | Equipment needed for manufacture (n=1) |  |  | Fibre quality (n=1) |
|  | Product processing time (n=1) |  |  | Porosity (n=1) |
|  | z-average (nm) (n=1) |  |  | Surface tension (n=1) |
|  | Molecule loading |  |  | Electroconductivity (n=1) |
|  | Frequency of discontinuation during therapy (n=1) |  |  | Density (n=1) |
|  | Frequency of significant irreversible adverse events (n=1) |  |  | Instrument type (n=1) |
|  | Protection from reinfection (n=1) |  |  | Speed of high speed homogenizer (n=1) |
|  | Special populations (n=1) |  |  | Amount of surfactant (stabilizer) (n=1) |
|  | Adjunct treatment (n=1) |  |  | Crystallinity of drug (n=1) |
|  | Need for screening (n=1) |  |  | Drug permeability study (n=1) |
|  | Need for monitoring (n=1) |  |  | Skin penetration (n=1) |
|  | Need for booster (n=1) |  |  | EE (n=1) |
|  | Expected financing source (n=1) |  |  | Turbidity (n=1) |
|  | Taste (n=1) |  |  | Structure (n=1) |
|  | (Intermediate step) critical features/quality attributes (n=1) |  |  | Application method (n=1) |
|  | Polymorphism (n=1) |  |  | Expected performance (n=1) |
|  | Solubility (n=1) |  |  | Nontarget organisms and environmental risk Assessment (n=1) |
|  | Rheological properties: Storage modulus (n=1) |  |  | Challenges/risks (n=1) |
|  | Rheological properties: G” (loss modulus) (n=1) |  |  | Adhesion (n=1) |
|  | Rheological properties: LVR region (linear viscoelastic region) (n=1) |  |  | Transfection efficiency (n=1) |
|  | Volatile materials content (n=1) |  |  | Cell cytotoxicity (n=1) |
|  | Sterility (n=1) |  |  | Splice correction (slope) (n=1) |
|  | Crystallinity of drug (n=1) |  |  | Phase separation under mechanical stress (n=1) |
|  | Heavy metals (n=1) |  |  |  |
|  | Key statement (n=1) |  |  |  |
|  | Reversibility (n=1) |  |  |  |
|  | Boxed warning (n=1) |  |  |  |
|  | Nonclinical toxicology (n=1) |  |  |  |
|  | Clinical studies (n=1) |  |  |  |
|  | Value proposition (n=1) |  |  |  |
|  | Study design (n=1) |  |  |  |
|  | Patents and exclusivities (n=1) |  |  |  |
|  | Patient share (n=1) |  |  |  |
|  | Product valuation (n=1) |  |  |  |
|  | Ejection force (n=1) |  |  |  |
|  | Tablet strength (n=1) |  |  |  |
|  | Empty capsid (n=1) |  |  |  |
|  | Replication-competent adeno-associated virus (n=1) |  |  |  |
|  | Adhesion (n=1) |  |  |  |
|  | Mechanical strength (n=1) |  |  |  |
|  | Spectrum of activity (n=1) |  |  |  |
|  | Convenience (n=1) |  |  |  |
|  | Crystalline shape (n=1) |  |  |  |
|  | Contact angle (n=1) |  |  |  |
|  | Patient age (n=1) |  |  |  |
|  | Patient access (n=1) |  |  |  |
|  | Pain (n=1) |  |  |  |
|  | Target user (n=1) |  |  |  |

Table S6: List of categories of TPP features. Each category contains a number of features (e.g., “scope” can contain features like indication, target population, target user, level of healthcare system implementation).

| **Category** | **Number of mentions in TPPs** |
| --- | --- |
| Scope | 19 |
| Operational characteristics | 13 |
| Performance characteristics | 10 |
| Pricing/cost | 4 |
| Test characteristics | 2 |
| Test performance | 2 |
| Drug-related characteristics | 2 |
| Product-related characteristics | 2 |
| Test procedure | 2 |
| Data output | 2 |
| Device characteristics | 1 |
| Test cartridge/strip | 1 |
| Process of production | 1 |
| Patient access/commercialisation | 1 |
| Legal-related characteristics | 1 |
| Accessibility | 1 |
| Manufacturing-related characteristics | 1 |
| Miscellaneous characteristics | 1 |
| Level of health care setting | 1 |
| healthcare system needs | 1 |
| Target user | 1 |
| Diagnosis moment | 1 |
| Technical specification | 1 |
| Reproducibility | 1 |
| Market overview | 1 |
| Patient/population needs | 1 |
| Regulatory needs | 1 |
| Commercial and sustainability needs | 1 |
| Scope of toolkit components | 1 |
| Clinical decision support algorithm, | 1 |
| Point of Care (POC) | 1 |
| App | 1 |
| System components | 1 |
| Functional requirements | 1 |

Table S7: Description of categories of TPP features.

| **Number of categories** | **Description of categories** | **Number of publications** |
| --- | --- | --- |
| **3** | Scope, performance, operational characteristics | 4 |
|  | Scope, device characteristics, test cartridge/strip | 1 |
|  | Scope, test characteristics, operational characteristics | 1 |
|  | Scope, test characteristics, test performance | 1 |
|  | Process of production, drug-related characteristics, product-related characteristics | 1 |
| **4** | Scope, performance characteristics, operational Characteristics, pricing | 3 |
|  | Scope, performance characteristics, operational characteristics, patient access/commercialisation | 1 |
|  | Drug-related characteristics, product-related characteristics, legal-related characteristics, manufacturing-related characteristics | 1 |
| **5** | Scope, performance characteristics, operational characteristics, cost characteristics, miscellaneous characteristics | 1 |
|  | Scope, level of health care setting, target user, diagnosis moment, operational characteristics | 1 |
|  | Scope, technical specification, reproducibility, operational characteristics, market overview | 1 |
|  | Scope, patient/population needs and performance characteristics, regulatory needs, healthcare system needs, commercial and sustainability needs | 1 |
|  | Scope, performance characteristics, test procedure, operational characteristics, pricing | 1 |
| **6** | Scope, test performance, test procedure, test results, consumables, operational characteristics | 1 |
|  | General scope, scope of toolkit components, clinical decision support algorithm, POC, app, data | 1 |
| **7** | Scope, system components, functional requirements, operational requirements, data characteristics, performance requirements, pricing and accessibility | 1 |
| NA | NA | 117 |
| Total |  | **138** |

Table S8: Overview of the consensus approaches used in TPP development.

| **Consensus approach** | **Authors** | **Year** | **Method/Tools** |
| --- | --- | --- | --- |
| **Formal** | Adepoyibi et al. | 2018 | Online survey, max-trade-off method. |
|  | Cruz et al. | 2019 | Expert meetings, online survey, 5-point Likert scale to decide on the TPP features. |
|  | Dailey et al. | 2019 | Delphi-like process. |
|  | Kadam et al. | 2020 | Delphi-like survey process, Likert scale. |
|  | Mather et al. | 2019 | Delphi process, online survey, Likert scale. |
|  | Németh et al. | 2020 | LeanQbD software, Pareto diagrams. |
|  | Nsanzabana et al. | 2018 | Consensus on TPP features. |
|  | Pallagi et al. | 2019 | Ishikawa diagram to visualise TPP features. |
|  | Chua et al. | 2017 | Consensus on TPP features. |
|  | Denkinger et al. | 2015 | Consensus on TPP features, Delphi-like process. |
|  | Donadeu et al. | 2017 | Consensus meetings. |
|  | Lim et al. | 2018 | Consensus on TPP features. |
|  | Pal et al. | 2016 | Survey. |
|  | Toskin et al. | 2017 | Delphi process. |
| **Formal and informal** | Alonso-Padilla et al. | 2020 | Face-to-face meetings, email surveys, Use-case scenarios for each of the test features. |
|  | Denkinger et al. | 2015 | Observations from national tuberculosis programs and surveys, market analysis, surveys, mathematical models were used, landscaping using FIND unpublished data where available to support decision making around optimal test characteristics. |
|  | Dittrich et al. | 2016 | Delphi approach, face-to-face interviews. |
|  | Gal et al. | 2018 | Quantitative and qualitative, online survey, workshops and in-person meetings. |
|  | Jaffar et al. | 2022 | 7-stage process: stages: (1) establishment of QTPP list based on publicly available information, (2) the identification of product quality attributes (QAs) and determination of their specifications based on the existing knowledge and initial analyses of several lots of Keytruda, (3) the determination of product CQAs using QbD risk assessment tools Risk Ranking & Filtering (Tool 1) and Preliminary Hazards Analysis (Tool 2), according to the instructions of ICH Q9 guideline; (4) prior knowledge-based identification of input process parameters (iPPs) and their impacts on CQAs and output process parameters (oPPs) to establish the manufacturing process and control strategy; (5) process evaluation via screening experiments to identify the CQAs that need to be controlled via optimization of their critical process parameters (CPPs); (6) optimization of CPPs to define their optimal design space for CQAs control; and (7) manufacturing process validation and risk acceptance upon the final repeat of risk assessment. |
|  | Lewin et al. | 2021 | Virtual meetings, email correspondence, interviews. In parallel, and a Delphi process through online surveys. |
|  | Pellé et al. | 2020 | Delphi process, online survey, Likert scale, in-person meetings. |
|  | Reipold et al. | 2017 | Key stakeholders mapping, Informal priority-setting exercise with key experts, Defining the key TPP domains, Delphi-like process, Likert scale. |
|  | Saydam and Takka | 2018 | Step-by-step risk analysis methods (qualitative and semi-quantitative). |
|  | Vetter et al. | 2021 | Delphi process, online survey, Likert scale, semi-structured qualitative interviews, virtual meetings. |
|  | Ding et al. | 2017 | Iterative and consensus-decision-making process, expert meetings, online survey. |
| **Informal** | Bengtson et al. | 2020 | Stepwise approach using design-thinking principles, gigamaps (large and information-dense diagrams that act as a bridge between inquiry, design, and implementation), visual depictions of the patient journeys, creation and validation of use Case Scenarios. |
|  | Fongwen et al. | 2021 | Face-to-face consultations, online meetings, semi-structured interviews with experts’ meetings. |
|  | Walsh et al. | 2022 | Virtual meetings. |
|  | Wyber et al. | 2016 | Expert meetings, email correspondence to choose key features. |
|  | Ebels et al. | 2014 | Field research, interviews. |

Table S9: Overview of the involved experts and their fields of expertise. TB: Tuberculosis; NGOs: non-governmental organizations; MOH: medical officer of health; PPP: public-private partnership;

| **Authors** | **Year** | **Experts** |
| --- | --- | --- |
| Adepoyibi et al. | 2018 | National TB Program Managers, laboratory personnel, donors, technical experts (including government, partner organizations, United Nations agencies), project managers/coordinators, and researchers. |
| Alonso-Padilla et al. | 2020 | Expert clinicians working with patients, researchers working in academia, and specialists in industry and product development partnerships (PDPs). |
| Bengtson et al. | 2020 | Health care providers in the field and scientific researchers. |
| Cruz et al. | 2019 | Academics, researchers, international NGOs, international organisations, international PDP, MOH, National programs. |
| Denkinger et al. | 2015 | WHO, researchers, clinicians, policy makers (global and national), and test developers. |
| Dittrich et al. | 2016 | World Health Organization (WHO), ReAct–Action on Antibiotic Resistance, Médecins sans Frontières (MSF) Access Campaign and the Foundation for Innovative New Diagnostics (FIND). |
| Fongwen et al. | 2021 | Partnership for Dengue Control and the Global Dengue, The International Diagnostics Centre (IDC) at the London School of Hygiene and Tropical Medicine (LSHTM). |
| Gal et al. | 2018 | Primary care clinicians, microbiologists, scientists (molecular microbiology, chemistry, and physics), diagnostic market experts and test developers from academia and industry (European Federation of Pharmaceutical Industries and Associations (EFPIA)) in Europe. |
| Kadam et al. | 2020 | Clinical practice, clinical research, diagnostic product development. |
| Lewin et al. | 2021 | Academia, civil society, industry, regulatory, health care providers, funders, stakeholders from implementation fields. |
| Mather et al. | 2019 | Clinical medicine, laboratory medicine, microbiology, diagnostics, public health, and global health. |
| Nsanzabana et al. | 2018 | Academic institutions, public health institutions and industry. |
| Pellé et al. | 2020 | Academics, industry, private and public sectors. |
| Reinhard-Rupp and Klohe | 2017 | Experts from WHO, academics, PPP (Merck, Lygature, Astellas Pharma, Swiss TPH, Farmanguinhos, Simcyp, SCI). |
| Reipold et al. | 2017 | In vitro diagnostics industry or product development partnerships/technical agencies/researchers, advocacy organizations, pharmaceutical industry, implementers/clinicians, national hepatitis programmes, international bodies, and consultants. |
| Vetter et al. | 2021 | Clinicians, researchers, laboratory experts, public health experts, procurer of POC. |
| Walsh et al. | 2022 | WHO experts, stakeholders from Medicines for Malaria Venture, PENTA Foundation, St George’s University, UK, and the Bill and Melinda Gates Foundation. |
| Wyber et al. | 2016 | Clinicians. |
| Ebels et al. | 2014 | Researchers, policymakers, manufacturers, and strategists, program personnel (persons who are working in or with the public health system to implement malaria strategies but who do not directly see patients or handle diagnostic tests), end users. |
| Chua et al. | 2017 | Public health experts, scientific community. |
| Denkinger et al. | 2015 | Researchers, clinicians, policy makers, test developers, and funders. |
| Ding et al. | 2017 | Experts from academic research institutions, national malaria control programmes, the WHO Global Malaria Programme, and WHO. |
| Donadeu et al. | 2017 | Diagnostics manufacturers, NGOs, industry, academia, research organisations/government agencies. |
| Lim et al. | 2018 | Researchers, WHO, implementers, regulators. |
| Pal et al. | 2016 | Diagnostic test end users from the armed forces. |
| Solomon et al. | 2012 | Disease experts, laboratory and field scientists, authorities on diagnostics, control programme managers, mathematical modellers, and health economists. |
| Toskin et al. | 2017 | Representatives from the companies/developers of interest. |

References

1. Adu-Gyamfi CG, Snyman T, Makhathini L, Otwombe K, Darboe F, Penn-Nicholson A, et al. Diagnostic accuracy of plasma kynurenine/tryptophan ratio, measured by enzyme-linked immunosorbent assay, for pulmonary tuberculosis. Int J Infect Dis. 2020 Oct 1;99:441–8.

2. Adu-Gyamfi C, Savulescu D, Mikhathani L, Otwombe K, Salazar-Austin N, Chaisson R, et al. Plasma Kynurenine-to-Tryptophan Ratio, a Highly Sensitive Blood-Based Diagnostic Tool for Tuberculosis in Pregnant Women Living With Human Immunodeficiency Virus (HIV). Clin Infect Dis. 2021 Sep 15;73(6):1027–36.

3. Ahmad R, Xie L, Pyle M, Suarez MF, Broger T, Steinberg D, et al. A rapid triage test for active pulmonary tuberculosis in adult patients with persistent cough. Sci Transl Med. 2019 Oct 23;11(515):eaaw8287.

4. Alonzo LF, Jain P, Hinkley T, Clute-Reinig N, Garing S, Spencer E, et al. Rapid, sensitive, and low-cost detection of Escherichia coli bacteria in contaminated water samples using a phage-based assay. Sci Rep. 2022 May 11;12(1):7741.

5. Armistead JS, Morlais I, Mathias DK, Jardim JG, Joy J, Fridman A, et al. Antibodies to a Single, Conserved Epitope in Anopheles APN1 Inhibit Universal Transmission of Plasmodium falciparum and Plasmodium vivax Malaria. Infect Immun. 2014 Jan 22;82(2):818–29.

6. Azimi T, Franzel L, Probst N. Seizing market shaping opportunities for vaccine cold chain equipment. Vaccine. 2017 Apr 19;35(17):2260–4.

7. Bachman CM, Grant BD, Anderson CE, Alonzo LF, Garing S, Byrnes SA, et al. Clinical validation of an open-access SARS-COV-2 antigen detection lateral flow assay, compared to commercially available assays. PLOS ONE. 2021 Aug 17;16(8):e0256352.

8. Balaña-Fouce R, Pérez Pertejo MY, Domínguez-Asenjo B, Gutiérrez-Corbo C, Reguera RM. Walking a tightrope: drug discovery in visceral leishmaniasis. Drug Discov Today. 2019 May;24(5):1209–16.

9. Bardon AR, Simoni JM, Layman LM, Stekler JD, Drain PK. Perspectives on the utility and interest in a point-of-care urine tenofovir test for adherence to HIV pre-exposure prophylaxis and antiretroviral therapy: an exploratory qualitative assessment among U.S. clients and providers. AIDS Res Ther. 2020 Aug 6;17(1):50.

10. Bartsch SM, Hotez PJ, Hertenstein DL, Diemert DJ, Zapf KM, Bottazzi ME, et al. Modeling the economic and epidemiologic impact of hookworm vaccine and mass drug administration (MDA) in Brazil, a high transmission setting. Vaccine. 2016 Apr 27;34(19):2197–206.

11. Basilico N, Parapini S, Sparatore A, Romeo S, Misiano P, Vivas L, et al. In Vivo and In Vitro Activities and ADME-Tox Profile of a Quinolizidine-Modified 4-Aminoquinoline: A Potent Anti-P. falciparum and Anti-P. vivax Blood-Stage Antimalarial. Mol Basel Switz. 2017 Dec 1;22(12):2102.

12. Bayaa R, Ndiaye MDB, Chedid C, Kokhreidze E, Tukvadze N, Banu S, et al. Multi-country evaluation of RISK6, a 6-gene blood transcriptomic signature, for tuberculosis diagnosis and treatment monitoring. Sci Rep. 2021 Jul 1;11(1):13646.

13. Beck A, Liu H. Macro- and Micro-Heterogeneity of Natural and Recombinant IgG Antibodies. Antibodies. 2019 Mar;8(1):18.

14. Hong-Geller E, Micheva-Viteva S, Benjamin B, Kanta Barman T, Chaira T, K. Paliwal J. Integration of Physicochemical and Pharmacokinetic Parameters in Lead Optimization: A Physiological Pharmacokinetic Model Based Approach. Curr Drug Discov Technol. 2010 Sep 1;7(3):143–53.

15. Bhargav E, Reddy YP, Koteshwara KB. Development and Optimization of Luliconazole Nanostructured Lipid Carriers Based Gel by Quality by Design its Skin Distribution Studies, Dermatokinetic Modeling & In-Vitro and Ex-Vivo Correlation. Curr Drug Deliv. 2021 Aug;18(7):1041–53.

16. Biagini GA, Fisher N, Shone AE, Mubaraki MA, Srivastava A, Hill A, et al. Generation of quinolone antimalarials targeting the *Plasmodium falciparum* mitochondrial respiratory chain for the treatment and prophylaxis of malaria. Proc Natl Acad Sci. 2012 May 22;109(21):8298–303.

17. Biter AB, Weltje S, Hudspeth EM, Seid CA, McAtee CP, Chen WH, et al. Characterization and Stability of Trypanosoma cruzi 24-C4 (Tc24-C4), a Candidate Antigen for a Therapeutic Vaccine Against Chagas Disease. J Pharm Sci. 2018 May;107(5):1468–73.

18. Bolla PK, Clark BA, Juluri A, Cheruvu HS, Renukuntla J. Evaluation of Formulation Parameters on Permeation of Ibuprofen from Topical Formulations Using Strat-M® Membrane. Pharmaceutics. 2020 Feb 13;12(2):151.

19. Bolton GR, Boesch AW, Basha J, LaCasse DP, Kelley BD, Acharya H. Effect of protein and solution properties on the donnan effect during the ultrafiltration of proteins. Biotechnol Prog. 2011 Jan;27(1):140–52.

20. Borges Sebastião I, Bhatnagar B, Tchessalov S. A Kinetic Model for Spray-Freezing of Pharmaceuticals. J Pharm Sci. 2021 May;110(5):2047–62.

21. Breder CD, Du W, Tyndall A. What’s the Regulatory Value of a Target Product Profile? Trends Biotechnol. 2017 Jul;35(7):576–9.

22. Broger T, Basu Roy R, Filomena A, Greef CH, Rimmele S, Havumaki J, et al. Diagnostic Performance of Tuberculosis-Specific IgG Antibody Profiles in Patients with Presumptive Tuberculosis from Two Continents. Clin Infect Dis. 2017 Apr 1;64(7):947–55.

23. Brussee JM, Neodo A, Schulz JD, Coulibaly JT, Pfister M, Keiser J. Pharmacometric Analysis of Tribendimidine Monotherapy and Combination Therapies To Achieve High Cure Rates in Patients with Hookworm Infections. Antimicrob Agents Chemother. 2021 Jan 20;65(2):e00714-20.

24. Buckner FS, Navabi N. Advances in Chagas disease drug development: 2009–2010: Curr Opin Infect Dis. 2010 Dec;23(6):609–16.

25. Burrows JN, Duparc S, Gutteridge WE, Hooft Van Huijsduijnen R, Kaszubska W, Macintyre F, et al. New developments in anti-malarial target candidate and product profiles. Malar J. 2017 Dec;16(1):26.

26. Callahan RL, Mehta NJ, Nanda K, Kopf GS. The new contraceptive revolution: developing innovative products outside of industry†,‡. Biol Reprod. 2020 Aug 4;103(2):157–66.

27. Calvet CM, Choi JY, Thomas D, Suzuki B, Hirata K, Lostracco-Johnson S, et al. 4-aminopyridyl-based lead compounds targeting CYP51 prevent spontaneous parasite relapse in a chronic model and improve cardiac pathology in an acute model of Trypanosoma cruzi infection. Almeida IC, editor. PLoS Negl Trop Dis. 2017 Dec 27;11(12):e0006132.

28. Campbell C. FDA 2011 Process Validation Guidance: Lifecycle Compliance Model. PDA J Pharm Sci Technol. 2014 Mar 1;68(2):185–91.

29. Campbell GA, Vallejo E. Primary Packaging Considerations in Developing Medicines for Children: Oral Liquid and Powder for Constitution. J Pharm Sci. 2015 Jan;104(1):52–62.

30. Charoo NA, Ali AA. Quality risk management in pharmaceutical development. Drug Dev Ind Pharm. 2013 Jul;39(7):947–60.

31. Chavez PF, Lebrun P, Sacré PY, De Bleye C, Netchacovitch L, Cuypers S, et al. Optimization of a pharmaceutical tablet formulation based on a design space approach and using vibrational spectroscopy as PAT tool. Int J Pharm. 2015 May;486(1–2):13–20.

32. Chen WH, Strych U, Hotez PJ, Bottazzi ME. The SARS-CoV-2 Vaccine Pipeline: an Overview. Curr Trop Med Rep. 2020 Jun;7(2):61–4.

33. Chirmule N, Khare R, Khandekar A, Jawa V. Failure Mode and Effects Analysis (FMEA) for Immunogenicity of Therapeutic Proteins. J Pharm Sci. 2020 Oct;109(10):3214–22.

34. Cocco P, Messenger MP, Smith AF, West RM, Shinkins B. Integrating Early Economic Evaluation into Target Product Profile development for medical tests: advantages and potential applications. Int J Technol Assess Health Care. 2021;37(1):e68.

35. Cocco P, Ayaz-Shah A, Messenger MP, West RM, Shinkins B. Target Product Profiles for medical tests: a systematic review of current methods. BMC Med. 2020 Dec;18(1):119.

36. Conde R, Laires R, Gonçalves LG, Rizvi A, Barroso C, Villar M, et al. Discovery of serum biomarkers for diagnosis of tuberculosis by NMR metabolomics including cross-validation with a second cohort. Biomed J. 2022 Aug;45(4):654–64.

37. Cook J, Cruañes MT, Gupta M, Riley S, Crison J. Quality-by-Design: Are We There Yet? AAPS PharmSciTech. 2014 Feb;15(1):140–8.

38. Craciunas L, Pickering O, Chu J, Žurauskienė J, Coomarasamy A. Target Product Profile for an endometrial receptivity test: women’s perspective. Eur J Obstet Gynecol Reprod Biol. 2020 Oct;253:42–7.

39. Csóka I, Pallagi E, Paál TL. Extension of quality-by-design concept to the early development phase of pharmaceutical R&D processes. Drug Discov Today. 2018 Jul;23(7):1340–3.

40. Dartois V, Barry CE. A medicinal chemists’ guide to the unique difficulties of lead optimization for tuberculosis. Bioorg Med Chem Lett. 2013 Sep;23(17):4741–50.

41. Ding L, Brunaugh AD, Stegemann S, Jermain SV, Herpin MJ, Kalafat J, et al. A Quality by Design Framework for Capsule-Based Dry Powder Inhalers. Pharmaceutics. 2021 Aug 6;13(8):1213.

42. Ditter D, Mahler HC, Roehl H, Wahl M, Huwyler J, Nieto A, et al. Characterization of surface properties of glass vials used as primary packaging material for parenterals. Eur J Pharm Biopharm. 2018 Apr;125:58–67.

43. Dostalek M, Prueksaritanont T, Kelley RF. Pharmacokinetic de-risking tools for selection of monoclonal antibody lead candidates. mAbs. 2017 Jul 4;9(5):756–66.

44. Drain PK, Gardiner J, Hannah H, Broger T, Dheda K, Fielding K, et al. Guidance for Studies Evaluating the Accuracy of Biomarker-Based Nonsputum Tests to Diagnose Tuberculosis. J Infect Dis. 2019 Oct 8;220(Supplement_3):S108–15.

45. Durbin A, Wilder-Smith A. An update on Zika vaccine developments. Expert Rev Vaccines. 2017 Aug 3;16(8):781–7.

46. Dybul M, Attoye T, Baptiste S, Cherutich P, Dabis F, Deeks SG, et al. The case for an HIV cure and how to get there. Lancet HIV. 2021 Jan;8(1):e51–8.

47. El Azab W. Contamination Control Strategy: Implementation Road Map. PDA J Pharm Sci Technol. 2021 Oct;75(5):445–53.

48. El-Say KM, Ahmed TA, Aljefri AH, El-Sawy HS, Fassihi R, Abou-Gharbia M. Oleic acid–reinforced PEGylated polymethacrylate transdermal film with enhanced antidyslipidemic activity and bioavailability of atorvastatin: A mechanistic ex-vivo/in-vivo analysis. Int J Pharm. 2021 Oct;608:121057.

49. Emperador DM, Mazzola LT, Wonderly Trainor B, Chua A, Kelly-Cirino C. Diagnostics for filovirus detection: impact of recent outbreaks on the diagnostic landscape. BMJ Glob Health. 2019;4(Suppl 2):e001112.

50. Entrican G, Charlier J, Dalton L, Messori S, Sharma S, Taylor R, et al. Construction of generic roadmaps for the strategic coordination of global research into infectious diseases of animals and zoonoses. Transbound Emerg Dis. 2021 May;68(3):1513–20.

51. Tiozzo Fasiolo L, Manniello MD, Bortolotti F, Buttini F, Rossi A, Sonvico F, et al. Anti-inflammatory flurbiprofen nasal powders for nose-to-brain delivery in Alzheimer’s disease. J Drug Target. 2019 Nov;27(9):984–94.

52. Leticia Fernandez-Carballo B, Escadafal C, MacLean E, Kapasi AJ, Dittrich S. Distinguishing bacterial versus non-bacterial causes of febrile illness - A systematic review of host biomarkers. J Infect. 2021 Apr;82(4):1–10.

53. Francis MJ. A Veterinary Vaccine Development Process Map to assist in the development of new vaccines. Vaccine. 2020 Jun 15;38(29):4512–5.

54. Funk CD, Laferrière C, Ardakani A. Target Product Profile Analysis of COVID-19 Vaccines in Phase III Clinical Trials and Beyond: An Early 2021 Perspective. Viruses. 2021 Mar 5;13(3):418.

55. Georghiou SB, Schumacher SG, Rodwell TC, Colman RE, Miotto P, Gilpin C, et al. Guidance for Studies Evaluating the Accuracy of Rapid Tuberculosis Drug-Susceptibility Tests. J Infect Dis. 2019 Oct 8;220(220 Suppl 3):S126–35.

56. Gerlinger C, Evers T, Rassen J, Wyss R. Using Real-World Data to Predict Clinical and Economic Benefits of a Future Drug Based on its Target Product Profile. Drugs - Real World Outcomes. 2020 Sep;7(3):221–7.

57. Gomez SA, Chapman LAC, Dilger E, Courtenay O, Picado A. Estimating the efficacy of community-wide use of systemic insecticides in dogs to control zoonotic visceral leishmaniasis: A modelling study in a Brazilian scenario. PLoS Negl Trop Dis. 2018 Sep;12(9):e0006797.

58. Goscé L, Winter JR, Taylor GS, Lewis JEA, Stagg HR. Modelling the dynamics of EBV transmission to inform a vaccine target product profile and future vaccination strategy. Sci Rep. 2019 Jun 26;9(1):9290.

59. Grant BD, Anderson CE, Alonzo LF, Garing SH, Williford JR, Baughman TA, et al. A SARS-CoV-2 coronavirus nucleocapsid protein antigen-detecting lateral flow assay. Ito E, editor. PLOS ONE. 2021 Nov 10;16(11):e0258819.

60. Günther G, Saathoff E, Rachow A, Ekandjo H, Diergaardt A, Marais N, et al. Clinical Evaluation of a Line-Probe Assay for Tuberculosis Detection and Drug-Resistance Prediction in Namibia. She RC, editor. Microbiol Spectr. 2022 Jun 29;10(3):e00259-22.

61. Gupta RK, Turner CT, Venturini C, Esmail H, Rangaka MX, Copas A, et al. Concise whole blood transcriptional signatures for incipient tuberculosis: a systematic review and patient-level pooled meta-analysis. Lancet Respir Med. 2020 Apr;8(4):395–406.

62. Halpern V, Stalter RM, Owen DH, Dorflinger LJ, Lendvay A, Rademacher KH. Towards the development of a longer-acting injectable contraceptive: past research and current trends. Contraception. 2015 Jul;92(1):3–9.

63. Ham AS, Nugent ST, Peters JJ, Katz DF, Shelter CM, Dezzutti CS, et al. The rational design and development of a dual chamber vaginal/rectal microbicide gel formulation for HIV prevention. Antiviral Res. 2015 Aug;120:153–64.

64. Ham AS, Rohan LC, Boczar A, Yang L, W. Buckheit K, Buckheit RW. Vaginal Film Drug Delivery of the Pyrimidinedione IQP-0528 for the Prevention of HIV Infection. Pharm Res. 2012 Jul;29(7):1897–907.

65. Ham AS, Ugaonkar SR, Shi L, Buckheit KW, Lakougna H, Nagaraja U, et al. Development of a Combination Microbicide Gel Formulation Containing IQP-0528 and Tenofovir for the Prevention of HIV Infection. J Pharm Sci. 2012 Apr;101(4):1423–35.

66. Hampson LV, Holzhauer B, Bornkamp B, Kahn J, Lange MR, Luo W, et al. A New Comprehensive Approach to Assess the Probability of Success of Development Programs Before Pivotal Trials. Clin Pharmacol Ther. 2022 May;111(5):1050–60.

67. Hemingway J. The role of vector control in stopping the transmission of malaria: threats and opportunities. Philos Trans R Soc B Biol Sci. 2014 Jun 19;369(1645):20130431.

68. Castillo Henríquez L, Vargas Zúñiga R, Carazo Berrocal G, Madrigal Redondo G, Calvo Guzmán B, Baltodano Viales E. Development of immediate release Rupatadine fumarate 10 mg tablets: A Quality by Design (QbD) approach. Drug Dev Ind Pharm. 2019 Oct 3;45(10):1674–81.

69. Hogan AB, Winskill P, Verity R, Griffin JT, Ghani AC. Modelling population-level impact to inform target product profiles for childhood malaria vaccines. BMC Med. 2018 Dec;16(1):109.

70. Hotez PJ, Beaumier CM, Gillespie PM, Strych U, Hayward T, Bottazzi ME. Advancing a vaccine to prevent hookworm disease and anemia. Vaccine. 2016 Jun;34(26):3001–5.

71. Jackson P, Borman P, Campa C, Chatfield M, Godfrey M, Hamilton P, et al. Using the Analytical Target Profile to Drive the Analytical Method Lifecycle. Anal Chem. 2019 Feb 19;91(4):2577–85.

72. Jacobs J, Kühne V, Lunguya O, Affolabi D, Hardy L, Vandenberg O. Implementing COVID-19 (SARS-CoV-2) Rapid Diagnostic Tests in Sub-Saharan Africa: A Review. Front Med. 2020 Oct 30;7:557797.

73. Januskaite P, Xu X, Ranmal SR, Gaisford S, Basit AW, Tuleu C, et al. I Spy with My Little Eye: A Paediatric Visual Preferences Survey of 3D Printed Tablets. Pharmaceutics. 2020 Nov 17;12(11).

74. Kapoor Y, Meyer RF, Ferguson HM, Skomski D, Daublain P, Troup GM, et al. Flexibility in Drug Product Development: A Perspective. Mol Pharm. 2021 Jul 5;18(7):2455–69.

75. Karlen W, Gan H, Chiu M, Dunsmuir D, Zhou G, Dumont GA, et al. Improving the Accuracy and Efficiency of Respiratory Rate Measurements in Children Using Mobile Devices. Penzel T, editor. PLoS ONE. 2014 Jun 11;9(6):e99266.

76. Karp CL, Lans D, Esparza J, Edson EB, Owen KE, Wilson CB, et al. Evaluating the value proposition for improving vaccine thermostability to increase vaccine impact in low and middle-income countries. Vaccine. 2015 Jul;33(30):3471–9.

77. Kauss T, Gaubert A, Boyer C, Ba BB, Manse M, Massip S, et al. Pharmaceutical development and optimization of azithromycin suppository for paediatric use. Int J Pharm. 2013 Jan;441(1–2):218–26.

78. Keiser J, Utzinger J. Antimalarials in the treatment of schistosomiasis. Curr Pharm Des. 2012;18(24):3531–8.

79. Khanolkar A, Thorat V, Raut P, Samanta G. Application of Quality by Design: Development to Manufacturing of Diclofenac Sodium Topical Gel. AAPS PharmSciTech. 2017 Oct;18(7):2754–63.

80. Khurana B, Arora D, Narang RK. FbD Supported Development and In Vitro Evaluation of Carbomer based Resveratrol Loaded Topical Antipsoriatic Nanoemulgel for its Targeted Skin Delivery. Pharm Nanotechnol. 2020;8(2):91–107.

81. Kik SV, Schumacher S, Cirillo DM, Churchyard G, Boehme C, Goletti D, et al. An evaluation framework for new tests that predict progression from tuberculosis infection to clinical disease. Eur Respir J. 2018 Oct;52(4):1800946.

82. Killeen GF, Chitnis N, Moore SJ, Okumu FO. Target product profile choices for intra-domiciliary malaria vector control pesticide products: repel or kill? Malar J. 2011 Dec;10(1):207.

83. Kondo H, Sako K. [Trade-offs in oral drug product development]. Yakugaku Zasshi. 2015;135(2):229–35.

84. Kotraiah V, Phares TW, Browne CD, Pannucci J, Mansour M, Noe AR, et al. Novel Peptide-Based PD1 Immunomodulators Demonstrate Efficacy in Infectious Disease Vaccines and Therapeutics. Front Immunol. 2020;11:264.

85. Kruchten SD, Bacon KM, Lee BY. The impact of human immunodeficiency virus (HIV) co-infection on the economic burden of cutaneous leishmaniasis (CL) in Brazil and potential value of new CL drug treatments. Am J Trop Med Hyg. 2014 Sep;91(3):520–7.

86. Kumar K, Chupakhin V, Vos A, Morrison D, Rassokhin D, Dellwo MJ, et al. Development and implementation of an enterprise-wide predictive model for early absorption, distribution, metabolism and excretion properties. Future Med Chem. 2021 Oct;13(19):1639–54.

87. Kumari P, Lavania S, Tyagi S, Dhiman A, Rath D, Anthwal D, et al. A novel aptamer-based test for the rapid and accurate diagnosis of pleural tuberculosis. Anal Biochem. 2019 Jan;564–565:80–7.

88. Kumari P, Dhiman A, Lavania S, Sharma P, Rath D, Anthwal D, et al. Assessment of DNA aptamers targeting GlcB and HspX antigens for application in the diagnosis of abdominal tuberculosis. Tuberculosis. 2022 May;134:102206.

89. Lafeber I, Ruijgrok EJ, Guchelaar HJ, Schimmel KJM. 3D Printing of Pediatric Medication: The End of Bad Tasting Oral Liquids?-A Scoping Review. Pharmaceutics. 2022 Feb 14;14(2).

90. Lee BY, McGlone SM. Pricing of new vaccines. Hum Vaccin. 2010 Aug;6(8):619–26.

91. Lingg N, Zhang P, Song Z, Bardor M. The sweet tooth of biopharmaceuticals: importance of recombinant protein glycosylation analysis. Biotechnol J. 2012 Dec;7(12):1462–72.

92. Liu H, Rivnay B, Avery K, Myung JH, Kozak D, Landrau N, et al. Optimization of the manufacturing process of a complex amphotericin B liposomal formulation using quality by design approach. Int J Pharm. 2020 Jul;585:119473.

93. Llibre A, Shimakawa Y, Mottez E, Ainsworth S, Buivan TP, Firth R, et al. Development and clinical validation of the Genedrive point-of-care test for qualitative detection of hepatitis C virus. Gut. 2018 Nov;67(11):2017–24.

94. Love MS, Beasley FC, Jumani RS, Wright TM, Chatterjee AK, Huston CD, et al. A high-throughput phenotypic screen identifies clofazimine as a potential treatment for cryptosporidiosis. Geary TG, editor. PLoS Negl Trop Dis. 2017 Feb 3;11(2):e0005373.

95. Luciani F, Galluzzo S, Gaggioli A, Kruse NA, Venneugues P, Schneider CK, et al. Implementing quality by design for biotech products: Are regulators on track? mAbs. 2015;7(3):451–5.

96. Lujan H, Griffin WC, Taube JH, Sayes CM. Synthesis and characterization of nanometer-sized liposomes for encapsulation and microRNA transfer to breast cancer cells. Int J Nanomedicine. 2019;14:5159–73.

97. Lukashevich IS, Paessler S, de la Torre JC. Lassa virus diversity and feasibility for universal prophylactic vaccine. F1000Research. 2019;8:F1000 Faculty Rev-134.

98. Lyu M, Cheng Y, Zhou J, Chong W, Wang Y, Xu W, et al. Systematic evaluation, verification and comparison of tuberculosis‐related non‐coding RNA diagnostic panels. J Cell Mol Med. 2021 Jan;25(1):184–202.

99. Mabille D, Ilbeigi K, Hendrickx S, Ungogo MA, Hulpia F, Lin C, et al. Nucleoside analogues for the treatment of animal trypanosomiasis. Int J Parasitol Drugs Drug Resist. 2022 Aug;19:21–30.

100. MacLean E, Broger T, Yerlikaya S, Fernandez-Carballo BL, Pai M, Denkinger CM. A systematic review of biomarkers to detect active tuberculosis. Nat Microbiol. 2019 May;4(5):748–58.

101. Makepeace BL, Jensen SA, Laney SJ, Nfon CK, Njongmeta LM, Tanya VN, et al. Immunisation with a multivalent, subunit vaccine reduces patent infection in a natural bovine model of onchocerciasis during intense field exposure. PLoS Negl Trop Dis. 2009 Nov 10;3(11):e544.

102. Malebo HM, D’Alessandro S, Ebstie YA, Sorè H, Tenoh Guedoung AR, Katani SJ, et al. In vitro Multistage Malaria Transmission Blocking Activity of Selected Malaria Box Compounds. Drug Des Devel Ther. 2020;14:1593–607.

103. Manga S, Perales R, Reaño M, D’Ambrosio L, Migliori GB, Amicosante M. Performance of a lateral flow immunochromatography test for the rapid diagnosis of active tuberculosis in a large multicentre study in areas with different clinical settings and tuberculosis exposure levels. J Thorac Dis. 2016 Nov;8(11):3307–13.

104. Marcantonio DH, Matteson A, Presler M, Burke JM, Hagen DR, Hua F, et al. Early Feasibility Assessment: A Method for Accurately Predicting Biotherapeutic Dosing to Inform Early Drug Discovery Decisions. Front Pharmacol. 2022;13:864768.

105. Marie AA, Salim MM, Kamal AH, Hammad SF, Elkhoudary MM. Analytical quality by design based on design space in reversed-phase-high performance liquid chromatography analysis for simultaneous estimation of metformin, linagliptin and empagliflozin. R Soc Open Sci. 2022 Jun;9(6):220215.

106. Marshall JM, Raban RR, Kandul NP, Edula JR, León TM, Akbari OS. Winning the Tug-of-War Between Effector Gene Design and Pathogen Evolution in Vector Population Replacement Strategies. Front Genet. 2019;10:1072.

107. Martín-Escolano J, Marín C, Rosales MJ, Tsaousis AD, Medina-Carmona E, Martín-Escolano R. An Updated View of the *Trypanosoma cruzi* Life Cycle: Intervention Points for an Effective Treatment. ACS Infect Dis. 2022 Jun 10;8(6):1107–15.

108. Martín-Escolano R, Molina-Carreño D, Plano D, Espuelas S, Rosales MJ, Moreno E, et al. Library of Selenocyanate and Diselenide Derivatives as In Vivo Antichagasic Compounds Targeting Trypanosoma cruzi Mitochondrion. Pharmaceuticals. 2021 May 1;14(5):419.

109. Martinez-Marquez D, Delmar Y, Sun S, Stewart RA. Exploring Macroporosity of Additively Manufactured Titanium Metamaterials for Bone Regeneration with Quality by Design: A Systematic Literature Review. Materials. 2020 Oct 27;13(21):4794.

110. Mduluza T, Mutapi F. Putting the treatment of paediatric schistosomiasis into context. Infect Dis Poverty. 2017 Dec;6(1):85.

111. Mendelsohn SC, Fiore-Gartland A, Penn-Nicholson A, Mulenga H, Mbandi SK, Borate B, et al. Validation of a host blood transcriptomic biomarker for pulmonary tuberculosis in people living with HIV: a prospective diagnostic and prognostic accuracy study. Lancet Glob Health. 2021 Jun;9(6):e841–53.

112. Meyer AJ, Ochom E, Turimumahoro P, Byanyima P, Sanyu I, Lalitha R, et al. C-Reactive Protein Testing for Active Tuberculosis among Inpatients without HIV in Uganda: a Diagnostic Accuracy Study. Diekema DJ, editor. J Clin Microbiol. 2020 Dec 17;59(1):e02162-20.

113. Michel MC, Michel-Reher MB, Hein P. A Systematic Review of Inverse Agonism at Adrenoceptor Subtypes. Cells. 2020 Aug 19;9(9):1923.

114. Milner E, McCalmont W, Bhonsle J, Caridha D, Carroll D, Gardner S, et al. Structure–activity relationships amongst 4-position quinoline methanol antimalarials that inhibit the growth of drug sensitive and resistant strains of Plasmodium falciparum. Bioorg Med Chem Lett. 2010 Feb;20(4):1347–51.

115. Valverde Mordt O, Tarral A, Strub-Wourgaft N. Development and Introduction of Fexinidazole into the Global Human African Trypanosomiasis Program. Am J Trop Med Hyg. 2022 Mar 15;106(5 Suppl):61–6.

116. Moreira FMF, Verma R, Pereira Dos Santos PC, Leite A, da Silva Santos A, de Araujo RCP, et al. Blood-based host biomarker diagnostics in active case finding for pulmonary tuberculosis: A diagnostic case-control study. EClinicalMedicine. 2021 Mar;33:100776.

117. Mukadi-Bamuleka D, Bulabula-Penge J, De Weggheleire A, Jacobs BKM, Edidi-Atani F, Mambu-Mbika F, et al. Field performance of three Ebola rapid diagnostic tests used during the 2018-20 outbreak in the eastern Democratic Republic of the Congo: a retrospective, multicentre observational study. Lancet Infect Dis. 2022 Jun;22(6):891–900.

118. Mulenga H, Zauchenberger CZ, Bunyasi EW, Mbandi SK, Mendelsohn SC, Kagina B, et al. Performance of diagnostic and predictive host blood transcriptomic signatures for Tuberculosis disease: A systematic review and meta-analysis. PloS One. 2020;15(8):e0237574.

119. Muller C, Mazel V, Dausset C, Busignies V, Bornes S, Nivoliez A, et al. Study of the Lactobacillus rhamnosus Lcr35® properties after compression and proposition of a model to predict tablet stability. Eur J Pharm Biopharm. 2014 Nov;88(3):787–94.

120. Murphy G, Pilcher CD, Keating SM, Kassanjee R, Facente SN, Welte A, et al. Moving towards a reliable HIV incidence test – current status, resources available, future directions and challenges ahead. Epidemiol Infect. 2017 Apr;145(5):925–41.

121. Mutavhatsindi H, van der Spuy GD, Malherbe ST, Sutherland JS, Geluk A, Mayanja-Kizza H, et al. Validation and Optimization of Host Immunological Bio-Signatures for a Point-of-Care Test for TB Disease. Front Immunol. 2021;12:607827.

122. Muzembo BA, Kitahara K, Debnath A, Okamoto K, Miyoshi SI. Accuracy of cholera rapid diagnostic tests: a systematic review and meta-analysis. Clin Microbiol Infect Off Publ Eur Soc Clin Microbiol Infect Dis. 2022 Feb;28(2):155–62.

123. Nukala PK, Palekar S, Patki M, Patel K. Abuse Deterrent Immediate Release Egg-Shaped Tablet (Egglets) Using 3D Printing Technology: Quality by Design to Optimize Drug Release and Extraction. AAPS PharmSciTech. 2019 Jan 15;20(2):80.

124. Ogoma SB, Moore SJ, Maia MF. A systematic review of mosquito coils and passive emanators: defining recommendations for spatial repellency testing methodologies. Parasit Vectors. 2012 Dec 7;5:287.

125. Olías-Molero AI, de la Fuente C, Cuquerella M, Torrado JJ, Alunda JM. Antileishmanial Drug Discovery and Development: Time to Reset the Model? Microorganisms. 2021 Dec 2;9(12).

126. Olliaro P, Seiler J, Kuesel A, Horton J, Clark JN, Don R, et al. Potential drug development candidates for human soil-transmitted helminthiases. PLoS Negl Trop Dis. 2011 Jun;5(6):e1138.

127. Orlandini S, Pinzauti S, Furlanetto S. Application of quality by design to the development of analytical separation methods. Anal Bioanal Chem. 2013 Jan;405(2–3):443–50.

128. Page S, Coupe A, Barrett A. An industrial perspective on the design and development of medicines for older patients. Int J Pharm. 2016 Oct;512(2):352–4.

129. Pan-Ngum W, Kinyanjui T, Kiti M, Taylor S, Toussaint JF, Saralamba S, et al. Predicting the relative impacts of maternal and neonatal respiratory syncytial virus (RSV) vaccine target product profiles: A consensus modelling approach. Vaccine. 2017 Jan;35(2):403–9.

130. Pantelias A, King JD, Lammie P, Weil GJ. Development and Introduction of the Filariasis Test Strip: A New Diagnostic Test for the Global Program to Eliminate Lymphatic Filariasis. Am J Trop Med Hyg. 2022 Mar 15;106(5 Suppl):56–60.

131. Paolantonacci P, Appourchaux P, Claudel B, Ollivier M, Dennett R, Siret L. Development of a Premium Quality Plasma-derived IVIg (IQYMUNE(®)) Utilizing the Principles of Quality by Design-A Worked-through Case Study. PDA J Pharm Sci Technol. 2018 Apr;72(2):176–87.

132. Patel SK, Rohan LC. On-demand microbicide products: design matters. Drug Deliv Transl Res. 2017 Dec;7(6):775–95.

133. Penn-Nicholson A, Georghiou SB, Ciobanu N, Kazi M, Bhalla M, David A, et al. Detection of isoniazid, fluoroquinolone, ethionamide, amikacin, kanamycin, and capreomycin resistance by the Xpert MTB/XDR assay: a cross-sectional multicentre diagnostic accuracy study. Lancet Infect Dis. 2022 Feb;22(2):242–9.

134. Penn-Nicholson A, Hraha T, Thompson EG, Sterling D, Mbandi SK, Wall KM, et al. Discovery and validation of a prognostic proteomic signature for tuberculosis progression: A prospective cohort study. Chaisson R, editor. PLOS Med. 2019 Apr 16;16(4):e1002781.

135. Pham MD, Wise A, Garcia ML, Van H, Zheng S, Mohamed Y, et al. Improving the coverage and accuracy of syphilis testing: The development of a novel rapid, point-of-care test for confirmatory testing of active syphilis infection and its early evaluation in China and South Africa. eClinicalMedicine. 2020 Jul;24:100440.

136. Pham NM, Rusch S, Temiz Y, Beck HP, Karlen W, Delamarche E. Immuno-gold silver staining assays on capillary-driven microfluidics for the detection of malaria antigens. Biomed Microdevices. 2019 Mar;21(1):24.

137. Phillips AN, Cambiano V, Nakagawa F, Ford D, Apollo T, Murungu J, et al. Point-of-Care Viral Load Testing for Sub-Saharan Africa: Informing a Target Product Profile. Open Forum Infect Dis. 2016 May 1;3(3):ofw161.

138. Pidathala C, Amewu R, Pacorel B, Nixon GL, Gibbons P, Hong WD, et al. Identification, design and biological evaluation of bisaryl quinolones targeting Plasmodium falciparum type II NADH:quinone oxidoreductase (PfNDH2). J Med Chem. 2012 Mar 8;55(5):1831–43.

139. Politis SN, Colombo P, Colombo G, Rekkas DM. Design of experiments (DoE) in pharmaceutical development. Drug Dev Ind Pharm. 2017;43(6):889–901.

140. Polli JE, Cook JA, Davit BM, Dickinson PA, Argenti D, Barbour N, et al. Summary workshop report: Facilitating oral product development and reducing regulatory burden through novel approaches to assess bioavailability/bioequivalence. AAPS J. 2012 Sep;14(3):627–38.

141. Pulkstenis E, Patra K, Zhang J. A Bayesian paradigm for decision-making in proof-of-concept trials. J Biopharm Stat. 2017;27(3):442–56.

142. Qin ZZ, Ahmed S, Sarker MS, Paul K, Adel ASS, Naheyan T, et al. Tuberculosis detection from chest x-rays for triaging in a high tuberculosis-burden setting: an evaluation of five artificial intelligence algorithms. Lancet Digit Health. 2021 Sep;3(9):e543–54.

143. Alfimeprase. Drugs RD. 2008;9(3):185–90.

144. Rathod V, Stagner WC, Gajera B, Haware RV. Hybridized nanoamorphous micellar dispersion using a QbD-DM(3) linked rational product design strategy for ritonavir: A BCS IV drug. Int J Pharm. 2020 Oct 15;588:119727.

145. Raw AS, Lionberger R, Yu LX. Pharmaceutical equivalence by design for generic drugs: modified-release products. Pharm Res. 2011 Jul;28(7):1445–53.

146. Rawal M, Singh A, Amiji MM. Quality-by-Design Concepts to Improve Nanotechnology-Based Drug Development. Pharm Res. 2019 Sep 3;36(11):153.

147. Rayfield WJ, Kandula S, Khan H, Tugcu N. Impact of Freeze/Thaw Process on Drug Substance Storage of Therapeutics. J Pharm Sci. 2017 Aug;106(8):1944–51.

148. Roudier B, Davit B, Schütz H, Cardot JM. Impact of data base structure in a successful in vitro-in vivo correlation for pharmaceutical products. AAPS J. 2015 Jan;17(1):24–34.

149. Salave S, Rana D, Benival D. Encapsulation of Anabolic Peptide in Lipid Nano Vesicles for Osteoporosis. Curr Protein Pept Sci. 2022 Aug 22;

150. Sam T, Ernest TB, Walsh J, Williams JL. A benefit/risk approach towards selecting appropriate pharmaceutical dosage forms – An application for paediatric dosage form selection. Int J Pharm. 2012 Oct;435(2):115–23.

151. Sammut-Powell C, Reynard C, Allen J, McDermott J, Braybrook J, Parisi R, et al. Examining the effect of evaluation sample size on the sensitivity and specificity of COVID-19 diagnostic tests in practice: a simulation study. Diagn Progn Res. 2022 Apr 25;6(1):12.

152. Santos LL, Wu EL, Grinias KM, Koetting MC, Jain P. Developability profile framework for lead candidate selection in topical dermatology. Int J Pharm. 2021 Jul 15;604:120750.

153. Schiefer A, Hübner MP, Krome A, Lämmer C, Ehrens A, Aden T, et al. Corallopyronin A for short-course anti-wolbachial, macrofilaricidal treatment of filarial infections. PLoS Negl Trop Dis. 2020 Dec;14(12):e0008930.

154. Schlesinger EB, Bernards DA, Chen HH, Feindt J, Cao J, Dix D, et al. Device design methodology and formulation of a protein therapeutic for sustained release intraocular delivery. Bioeng Transl Med. 2019 Jan;4(1):152–63.

155. Schneider JE, Boehme C, Borisch B, Dittrich S. Application of a simple point-of-care test to reduce UK healthcare costs and adverse events in outpatient acute respiratory infections. J Med Econ. 2020 Jul;23(7):673–82.

156. Selen A, Dickinson PA, Müllertz A, Crison JR, Mistry HB, Cruañes MT, et al. The biopharmaceutics risk assessment roadmap for optimizing clinical drug product performance. J Pharm Sci. 2014 Nov;103(11):3377–97.

157. Shah P, Goodyear B, Haq A, Puri V, Michniak-Kohn B. Evaluations of Quality by Design (QbD) Elements Impact for Developing Niosomes as a Promising Topical Drug Delivery Platform. Pharmaceutics. 2020 Mar 9;12(3):246.

158. Shoukat A, Vilches T, Moghadas SM. Cost-effectiveness of a potential Zika vaccine candidate: a case study for Colombia. BMC Med. 2018 Jul 3;16(1):100.

159. Silva J, Mendes M, Cova T, Sousa J, Pais A, Vitorino C. Unstructured Formulation Data Analysis for the Optimization of Lipid Nanoparticle Drug Delivery Vehicles. AAPS PharmSciTech. 2018 Jul;19(5):2383–94.

160. Simpson S, Kaufmann MC, Glozman V, Chakrabarti A. Disease X: accelerating the development of medical countermeasures for the next pandemic. Lancet Infect Dis. 2020 May;20(5):e108–15.

161. Singhai VD, Maheshwari R, Sharma S, Paliwal S. Employment of Quality by Design Approach via Response Surface Methodology to Optimize and Develop Modified-release Formulation of Hydrochlorothiazide. Curr Comput Aided Drug Des. 2021;17(2):266–80.

162. Soeiro M de NC. Perspectives for a new drug candidate for Chagas disease therapy. Mem Inst Oswaldo Cruz. 2022;117:e220004.

163. Specht S, Pfarr KM, Arriens S, Hübner MP, Klarmann-Schulz U, Koschel M, et al. Combinations of registered drugs reduce treatment times required to deplete Wolbachia in the Litomosoides sigmodontis mouse model. PLoS Negl Trop Dis. 2018 Jan;12(1):e0006116.

164. Spooner E, Reddy T, Mchunu N, Reddy S, Daniels B, Ngomane N, et al. Point-of-care CD4 testing: Differentiated care for the most vulnerable. J Glob Health. 2022;12:04004.

165. Stegemann S. Patient centric drug product design in modern drug delivery as an opportunity to increase safety and effectiveness. Expert Opin Drug Deliv. 2018 Jun 3;15(6):619–27.

166. Stegemann S, Sheehan L, Rossi A, Barrett A, Paudel A, Crean A, et al. Rational and practical considerations to guide a target product profile for patient-centric drug product development with measurable patient outcomes – A proposed roadmap. Eur J Pharm Biopharm. 2022 Aug;177:81–8.

167. Stevenson D, Cochrane T. Implementation of QbD Part 1 - Setting product specifications. Regul Rapp. 2011;8(2):16–8.

168. Subramanian VB, Katari NK, Ponnam V, Konduru N, Dongala T, Marisetti VM, et al. Stability-indicating reversed-phase-HPLC method development and validation for sacubitril/valsartan complex in the presence of impurities and degradation products: Robustness by quality-by-design approach. Biomed Chromatogr BMC. 2022 Jan;36(1):e5240.

169. Sulaiman TNS, Larasati D, Nugroho AK, Choiri S. Assessment of the Effect of PLGA Co-polymers and PEG on the Formation and Characteristics of PLGA-PEG-PLGA Co-block Polymer Using Statistical Approach. Adv Pharm Bull. 2019 Aug;9(3):382–92.

170. Sumner T, Fiore-Gartland A, Hatherill M, Houben RMGJ, Scriba TJ, White RG. The effect of new Mycobacterium tuberculosis infection on the sensitivity of prognostic TB signatures. Int J Tuberc Lung Dis Off J Int Union Tuberc Lung Dis. 2021 Dec 1;25(12):1001–5.

171. Sutherland JS, van der Spuy G, Gindeh A, Thuong NTT, Namuganga A, Owolabi O, et al. Diagnostic Accuracy of the Cepheid 3-gene Host Response Fingerstick Blood Test in a Prospective, Multi-site Study: Interim Results. Clin Infect Dis Off Publ Infect Dis Soc Am. 2022 Jul 6;74(12):2136–41.

172. Swain S, Parhi R, Jena BR, Babu SM. Quality by Design: Concept to Applications. Curr Drug Discov Technol. 2019;16(3):240–50.

173. Tan L, Chin SF, Miner VW, Dong L, Gupta S, Fields SM. Determination of apomorphine freebase in sublingual tablets by proton nuclear magnetic resonance spectroscopy. J Pharm Biomed Anal. 2016 Sep;129:378–82.

174. Tari LW. The Utility of Structural Biology in Drug Discovery. In: Tari LW, editor. Structure-Based Drug Discovery [Internet]. Totowa, NJ: Humana Press; 2012 [cited 2023 Dec 21]. p. 1–27. (Methods in Molecular Biology; vol. 841). Available from: https://link.springer.com/10.1007/978-1-61779-520-6_1

175. Teng Z, Gupta N, Hua Z, Liu G, Samnotra V, Venkatakrishnan K, et al. Model-Based Meta-Analysis for Multiple Myeloma: A Quantitative Drug-Independent Framework for Efficient Decisions in Oncology Drug Development. Clin Transl Sci. 2018 Mar;11(2):218–25.

176. Thakkar R, Thakkar R, Pillai A, Ashour EA, Repka MA. Systematic screening of pharmaceutical polymers for hot melt extrusion processing: a comprehensive review. Int J Pharm. 2020 Feb;576:118989.

177. Than YM, Titapiwatanakun V. Tailoring immediate release FDM 3D printed tablets using a quality by design (QbD) approach. Int J Pharm. 2021 Apr 15;599:120402.

178. Tietje K, Hawkins K, Clerk C, Ebels K, McGray S, Crudder C, et al. The essential role of infection-detection technologies for malaria elimination and eradication. Trends Parasitol. 2014 May;30(5):259–66.

179. Tolley EE, McKenna K, Mackenzie C, Ngabo F, Munyambanza E, Arcara J, et al. Preferences for a potential longer-acting injectable contraceptive: perspectives from women, providers, and policy makers in Kenya and Rwanda. Glob Health Sci Pract. 2014 May;2(2):182–94.

180. Troiano G, Nolan J, Parsons D, Van Geen Hoven C, Zale S. A Quality by Design Approach to Developing and Manufacturing Polymeric Nanoparticle Drug Products. AAPS J. 2016 Nov;18(6):1354–65.

181. Turner CT, Gupta RK, Tsaliki E, Roe JK, Mondal P, Nyawo GR, et al. Blood transcriptional biomarkers for active pulmonary tuberculosis in a high-burden setting: a prospective, observational, diagnostic accuracy study. Lancet Respir Med. 2020 Apr;8(4):407–19.

182. Turner JA, Ruscoe CNE, Perrior TR. Discovery to Development: Insecticides for Malaria Vector Control. CHIMIA. 2016 Oct 26;70(10):684.

183. Tyndall A, Du W, Breder CD. Regulatory watch: The target product profile as a tool for regulatory communication: advantageous but underused. Nat Rev Drug Discov. 2017 Mar;16(3):156.

184. Vannice KS, Giersing BK, Kaslow DC, Griffiths E, Meyer H, Barrett A, et al. Meeting Report: WHO consultation on considerations for regulatory expectations of Zika virus vaccines for use during an emergency. Vaccine. 2019 Nov 28;37(50):7443–50.

185. Visser JC, Dohmen WMC, Hinrichs WLJ, Breitkreutz J, Frijlink HW, Woerdenbag HJ. Quality by design approach for optimizing the formulation and physical properties of extemporaneously prepared orodispersible films. Int J Pharm. 2015 May 15;485(1–2):70–6.

186. Wang T, McAuslane N, Goettsch WG, Leufkens HGM, De Bruin ML. Challenges and Opportunities for Companies to Build HTA/Payer Perspectives Into Drug Development Through the Use of a Dynamic Target Product Profile. Front Pharmacol. 2022 Jul 18;13:948161.

187. Warman AJ, Rito TS, Fisher NE, Moss DM, Berry NG, O’Neill PM, et al. Antitubercular pharmacodynamics of phenothiazines. J Antimicrob Chemother. 2013 Apr;68(4):869–80.

188. Warsinske HC, Rao AM, Moreira FMF, Santos PCP, Liu AB, Scott M, et al. Assessment of Validity of a Blood-Based 3-Gene Signature Score for Progression and Diagnosis of Tuberculosis, Disease Severity, and Treatment Response. JAMA Netw Open. 2018 Oct 5;1(6):e183779.

189. Watson TJ, Nosal R. Scientific opportunities through quality by design. In: Chemical Engineering in the Pharmaceutical Industry [Internet]. 2019. p. 1067–71. Available from: https://www.scopus.com/inward/record.uri?eid=2-s2.0-85103345185&doi=10.1002%2f9781119600800.ch48&partnerID=40&md5=78fb3feb17e15f902e1b355632e2f7fa

190. Wen Y, Jawa V. The Impact of Product and Process Related Critical Quality Attributes on Immunogenicity and Adverse Immunological Effects of Biotherapeutics. J Pharm Sci. 2021 Mar;110(3):1025–41.

191. White NJ, Nosten FH. SERCAP: is the perfect the enemy of the good? Malar J. 2021 Jun 24;20(1):281.

192. Winter JR, Taylor GS, Thomas OG, Jackson C, Lewis JEA, Stagg HR. Predictors of Epstein-Barr virus serostatus in young people in England. BMC Infect Dis. 2019 Nov 28;19(1):1007.

193. Wyatt PG, Gilbert IH, Read KD, Fairlamb AH. Target validation: linking target and chemical properties to desired product profile. Curr Top Med Chem. 2011;11(10):1275–83.

194. Xu Y, Constantine F, Yuan Y, Pritchett YL. ASIED: a Bayesian adaptive subgroup-identification enrichment design. J Biopharm Stat. 2020 Jul 3;30(4):623–38.

195. Yang PY, Hui CJ, Tien DJ, Snowden AW, Derfus GE, Opel CF. Accurate definition of control strategies using cross validated stepwise regression and Monte Carlo simulation. J Biotechnol. 2019;306:100006.

196. Yao B, Zhang D, Cao W, Yang G, Li W, Hui X, et al. Drifts in N-Linked Glycosylation Result in ADCC Potency Variation of Perjeta® from August 2020 to October 2021 in China. Siemianowicz K, editor. BioMed Res Int. 2022 Apr 30;2022:1–13.

197. Yost E, Mazel V, Sluga KK, Nagapudi K, Muliadi AR. Beyond Brittle/Ductile Classification: Applying Proper Constitutive Mechanical Metrics to Understand the Compression Characteristics of Pharmaceutical Materials. J Pharm Sci. 2022 Jul;111(7):1984–91.

198. Yu LX, Amidon G, Khan MA, Hoag SW, Polli J, Raju GK, et al. Understanding Pharmaceutical Quality by Design. AAPS J. 2014 Jul;16(4):771–83.

199. Chua A, Prat I, Nuebling CM, Wood D, Moussy F. Update on Zika Diagnostic Tests and WHO’s Related Activities. De Silva AM, editor. PLoS Negl Trop Dis. 2017 Feb 2;11(2):e0005269.

200. Denkinger CM, Kik SV, Cirillo DM, Casenghi M, Shinnick T, Weyer K, et al. Defining the Needs for Next Generation Assays for Tuberculosis. J Infect Dis. 2015 Apr;211(suppl_2):S29–38.

201. Ding XC, Ade MP, Baird JK, Cheng Q, Cunningham J, Dhorda M, et al. Defining the next generation of Plasmodium vivax diagnostic tests for control and elimination: Target product profiles. Pimenta PF, editor. PLoS Negl Trop Dis. 2017 Apr 3;11(4):e0005516.

202. Donadeu M, Fahrion AS, Olliaro PL, Abela-Ridder B. Target product profiles for the diagnosis of Taenia solium taeniasis, neurocysticercosis and porcine cysticercosis. Garcia HH, editor. PLoS Negl Trop Dis. 2017 Sep 11;11(9):e0005875.

203. Ebels KB, Clerk C, Crudder CH, McGray S, Magnuson K, Tietje K, et al. Incorporating user needs into product development for improved infection detection for malaria elimination programs. In: IEEE Global Humanitarian Technology Conference (GHTC 2014) [Internet]. San Jose, CA: IEEE; 2014 [cited 2023 Nov 2]. p. 555–60. Available from: http://ieeexplore.ieee.org/document/6970338/

204. Lim MD, Brooker SJ, Belizario VY, Gay-Andrieu F, Gilleard J, Levecke B, et al. Diagnostic tools for soil-transmitted helminths control and elimination programs: A pathway for diagnostic product development. Maruyama H, editor. PLoS Negl Trop Dis. 2018 Mar 1;12(3):e0006213.

205. Pal S, Jasper LE, Lawrence KL, Walter M, Gilliland T, Dauner AL, et al. Assessing the Dengue Diagnosis Capability Gap in the Military Health System. Mil Med. 2016 Aug;181(8):756–66.

206. Solomon AW, Engels D, Bailey RL, Blake IM, Brooker S, Chen JX, et al. A Diagnostics Platform for the Integrated Mapping, Monitoring, and Surveillance of Neglected Tropical Diseases: Rationale and Target Product Profiles. McCarthy JS, editor. PLoS Negl Trop Dis. 2012 Jul 31;6(7):e1746.

207. Toskin I, Murtagh M, Peeling RW, Blondeel K, Cordero J, Kiarie J. Advancing prevention of sexually transmitted infections through point-of-care testing: target product profiles and landscape analysis. Sex Transm Infect. 2017 Dec;93(S4):S69–80.

208. Alafeef M, Pan D. Diagnostic Approaches For COVID-19: Lessons Learned and the Path Forward. ACS Nano. 2022 Aug 23;16(8):11545–76.

209. Alonso-Padilla J, Abril M, Alarcón De Noya B, Almeida IC, Angheben A, Araujo Jorge T, et al. Target product profile for a test for the early assessment of treatment efficacy in Chagas disease patients: An expert consensus. Santiago HDC, editor. PLoS Negl Trop Dis. 2020 Apr 23;14(4):e0008035.

210. Amasya G, Badilli U, Aksu B, Tarimci N. Quality by design case study 1: Design of 5-fluorouracil loaded lipid nanoparticles by the W/O/W double emulsion — Solvent evaporation method. Eur J Pharm Sci. 2016 Mar;84:92–102.

211. Apolinário AC, Ferraro RB, De Oliveira CA, Pessoa Jr A, De Oliveira Rangel-Yagui C. Quality-by-Design Approach for Biological API Encapsulation into Polymersomes Using “Off-the-Shelf” Materials: a Study on L-Asparaginase. AAPS PharmSciTech. 2019 Aug;20(6):251.

212. Arnold SLM. Target Product Profile and Development Path for Shigellosis Treatment with Antibacterials. ACS Infect Dis. 2021 May 14;7(5):948–58.

213. Arora D, Nanda S. Quality by design driven development of resveratrol loaded ethosomal hydrogel for improved dermatological benefits via enhanced skin permeation and retention. Int J Pharm. 2019 Aug;567:118448.

214. Arranja A, Gouveia LF, Gener P, Rafael DF, Pereira C, Schwartz S, et al. Self-assembly PEGylation assists SLN-paclitaxel delivery inducing cancer cell apoptosis upon internalization. Int J Pharm. 2016 Mar;501(1–2):180–9.

215. Awotwe-Otoo D, Agarabi C, Wu GK, Casey E, Read E, Lute S, et al. Quality by design: Impact of formulation variables and their interactions on quality attributes of a lyophilized monoclonal antibody. Int J Pharm. 2012 Nov;438(1–2):167–75.

216. Beg S, Katare OP, Singh B. Formulation by design approach for development of ultrafine self-nanoemulsifying systems of rosuvastatin calcium containing long-chain lipophiles for hyperlipidemia management. Colloids Surf B Biointerfaces. 2017 Nov;159:869–79.

217. Bell M, Webster L, Woodland A. Research Techniques Made Simple: An Introduction to Drug Discovery for Dermatology. J Invest Dermatol. 2019 Nov;139(11):2252-2257.e1.

218. Bengtson M, Bharadwaj M, Bosch AT, Nyakundi H, Matoke-Muhia D, Dekker C, et al. Matching Development of Point-of-Care Diagnostic Tests to the Local Context: A Case Study of Visceral Leishmaniasis in Kenya and Uganda. Glob Health Sci Pract. 2020 Sep 30;8(3):549–65.

219. Bergström F, Lindmark B. Accelerated drug discovery by rapid candidate drug identification. Drug Discov Today. 2019 Jun;24(6):1237–41.

220. Bernatchez JA, Tran LT, Li J, Luan Y, Siqueira-Neto JL, Li R. Drugs for the Treatment of Zika Virus Infection. J Med Chem. 2020 Jan 23;63(2):470–89.

221. Burrows JN, Duparc S, Gutteridge WE, Hooft Van Huijsduijnen R, Kaszubska W, Macintyre F, et al. New developments in anti-malarial target candidate and product profiles. Malar J. 2017 Dec;16(1):26.

222. Burrows J, Slater H, Macintyre F, Rees S, Thomas A, Okumu F, et al. A discovery and development roadmap for new endectocidal transmission-blocking agents in malaria. Malar J. 2018 Dec;17(1):462.

223. Campbell A, Brieva T, Raviv L, Rowley J, Niss K, Brandwein H, et al. Concise Review: Process Development Considerations for Cell Therapy. Stem Cells Transl Med. 2015 Oct 1;4(10):1155–63.

224. Carballar-Lejarazú R, Ogaugwu C, Tushar T, Kelsey A, Pham TB, Murphy J, et al. Next-generation gene drive for population modification of the malaria vector mosquito, *Anopheles gambiae*. Proc Natl Acad Sci. 2020 Sep 15;117(37):22805–14.

225. Chang RK, Raw A, Lionberger R, Yu L. Generic Development of Topical Dermatologic Products, Part II: Quality by Design for Topical Semisolid Products. AAPS J. 2013 Jul;15(3):674–83.

226. Chappidi SR, Bhargav E, Marikunte V, Chinthaginjala H, Vijaya Jyothi M, Pisay M, et al. A Cost Effective (QbD) Approach in the Development and Optimization of Rosiglitazone Maleate Mucoadhesive Extended Release Tablets – In Vitro and Ex Vivo. Adv Pharm Bull. 2019 Jun 1;9(2):281–8.

227. Charoo NA, Shamsher AAA, Zidan AS, Rahman Z. Quality by design approach for formulation development: A case study of dispersible tablets. Int J Pharm. 2012 Feb;423(2):167–78.

228. Chudiwal SS, Dehghan MHG. Quality by design approach for development of suspension nasal spray products: a case study on budesonide nasal suspension. Drug Dev Ind Pharm. 2016 Oct 2;42(10):1643–52.

229. Chudiwal SS, Dehghan MHG. Quality by design (QbD) approach for design and development of drug-device combination products: a case study on flunisolide nasal spray. Pharm Dev Technol. 2018 Nov 26;23(10):1077–87.

230. Chudiwal VS, Shahi S, Chudiwal S. Development of sustained release gastro-retentive tablet formulation of nicardipine hydrochloride using quality by design (QbD) approach. Drug Dev Ind Pharm. 2018 May 4;44(5):787–99.

231. Costa CP, Cunha S, Moreira JN, Silva R, Gil-Martins E, Silva V, et al. Quality by design (QbD) optimization of diazepam-loaded nanostructured lipid carriers (NLC) for nose-to-brain delivery: Toxicological effect of surface charge on human neuronal cells. Int J Pharm. 2021 Sep;607:120933.

232. Crcarevska M, Dimitrovska A, Sibinovska N, Mladenovska K, Slavevska Raicki R, Glavas Dodov M. Implementation of quality by design principles in the development of microsponges as drug delivery carriers: Identification and optimization of critical factors using multivariate statistical analyses and design of experiments studies. Int J Pharm. 2015 Jul;489(1–2):58–72.

233. Cruz I, Albertini A, Barbeitas M, Arana B, Picado A, Ruiz-Postigo JA, et al. Target Product Profile for a point-of-care diagnostic test for dermal leishmaniases. Parasite Epidemiol Control. 2019 May;5:e00103.

234. Cunha S, Costa CP, Loureiro JA, Alves J, Peixoto AF, Forbes B, et al. Double Optimization of Rivastigmine-Loaded Nanostructured Lipid Carriers (NLC) for Nose-to-Brain Delivery Using the Quality by Design (QbD) Approach: Formulation Variables and Instrumental Parameters. Pharmaceutics. 2020 Jun 28;12(7):599.

235. Dailey P, Osborn J, Ashley E, Baron E, Dance D, Fusco D, et al. Defining System Requirements for Simplified Blood Culture to Enable Widespread Use in Resource-Limited Settings. Diagnostics. 2019 Jan 11;9(1):10.

236. Dalal R, Shah J, Gorain B, Choudhury H, Jacob S, Mehta TA, et al. Development and Optimization of Asenapine Sublingual Film Using QbD Approach. AAPS PharmSciTech. 2021 Oct;22(7):244.

237. Deng Y, Zhong G, Wang Y, Wang N, Yu Q, Yu X. Quality by design approach for the preparation of fat-soluble vitamins lipid injectable emulsion. Int J Pharm. 2019 Nov;571:118717.

238. Denkinger CM, Dolinger D, Schito M, Wells W, Cobelens F, Pai M, et al. Target Product Profile of a Molecular Drug-Susceptibility Test for Use in Microscopy Centers. J Infect Dis. 2015 Apr;211(suppl_2):S39–49.

239. Dittrich S, Tadesse BT, Moussy F, Chua A, Zorzet A, Tängdén T, et al. Target Product Profile for a Diagnostic Assay to Differentiate between Bacterial and Non-Bacterial Infections and Reduce Antimicrobial Overuse in Resource-Limited Settings: An Expert Consensus. Yansouni C, editor. PLOS ONE. 2016 Aug 25;11(8):e0161721.

240. Dormenval C, Lokras A, Cano-Garcia G, Wadhwa A, Thanki K, Rose F, et al. Identification of Factors of Importance for Spray Drying of Small Interfering RNA-Loaded Lipidoid-Polymer Hybrid Nanoparticles for Inhalation. Pharm Res. 2019 Oct;36(10):142.

241. Fongwen N, Wilder-Smith A, Gubler DJ, Ooi EE, T. Salvana EM, De Lamballerie X, et al. Target product profile for a dengue pre-vaccination screening test. Gawarammana I, editor. PLoS Negl Trop Dis. 2021 Jul 29;15(7):e0009557.

242. Funk CD, Laferrière C, Ardakani A. A Snapshot of the Global Race for Vaccines Targeting SARS-CoV-2 and the COVID-19 Pandemic. Front Pharmacol. 2020 Jun 19;11:937.

243. Gal M, Francis NA, Hood K, Villacian J, Goossens H, Watkins A, et al. Matching diagnostics development to clinical need: Target product profile development for a point of care test for community-acquired lower respiratory tract infection. Gupta V, editor. PLOS ONE. 2018 Aug 1;13(8):e0200531.

244. García-Basteiro AL, DiNardo A, Saavedra B, Silva DR, Palmero D, Gegia M, et al. Point of care diagnostics for tuberculosis. Pulmonology. 2018 Mar;24(2):73–85.

245. Garg B, Katare OP, Beg S, Lohan S, Singh B. Systematic development of solid self-nanoemulsifying oily formulations (S-SNEOFs) for enhancing the oral bioavailability and intestinal lymphatic uptake of lopinavir. Colloids Surf B Biointerfaces. 2016 May;141:611–22.

246. Garg S, Tambwekar KR, Vermani K, Kandarapu R, Garg A, Waller DP, et al. Development Pharmaceutics of Microbicide Formulations. Part II: Formulation, Evaluation, and Challenges. AIDS Patient Care STDs. 2003 Aug;17(8):377–99.

247. Gavan A, Porfire A, Marina C, Tomuta I. Formulation and pharmaceutical development of quetiapine fumarate sustained release matrix tablets using a QbD approach. Acta Pharm. 2017 Mar 1;67(1):53–70.

248. Gurumukhi VC, Bari SB. Development of ritonavir-loaded nanostructured lipid carriers employing quality by design (QbD) as a tool: characterizations, permeability, and bioavailability studies. Drug Deliv Transl Res. 2022 Jul;12(7):1753–73.

249. Ha JM, Seo JW, Kim SH, Kim JY, Park CW, Rhee YS, et al. Implementation of Quality by Design for Formulation of Rebamipide Gastro-retentive Tablet. AAPS PharmSciTech. 2017 Nov;18(8):3129–39.

250. Hales D, Vlase L, Porav SA, Bodoki A, Barbu-Tudoran L, Achim M, et al. A quality by design (QbD) study on enoxaparin sodium loaded polymeric microspheres for colon-specific delivery. Eur J Pharm Sci. 2017 Mar;100:249–61.

251. Hastings IM, Hodel EM. Pharmacological considerations in the design of anti-malarial drug combination therapies – is matching half-lives enough? Malar J. 2014 Dec;13(1):62.

252. Heal DJ, Smith SL. Prospects for new drugs to treat binge-eating disorder: Insights from psychopathology and neuropharmacology. J Psychopharmacol (Oxf). 2022 Jun;36(6):680–703.

253. Hernandez-Morales I, Van Loock M. An Industry Perspective on Dengue Drug Discovery and Development. In: Hilgenfeld R, Vasudevan SG, editors. Dengue and Zika: Control and Antiviral Treatment Strategies [Internet]. Singapore: Springer Singapore; 2018 [cited 2023 Nov 2]. p. 333–53. (Advances in Experimental Medicine and Biology; vol. 1062). Available from: http://link.springer.com/10.1007/978-981-10-8727-1_23

254. Huston CD, Spangenberg T, Burrows J, Willis P, Wells TNC, Van Voorhis W. A Proposed Target Product Profile and Developmental Cascade for New Cryptosporidiosis Treatments. Vinetz JM, editor. PLoS Negl Trop Dis. 2015 Oct 8;9(10):e0003987.

255. Ignjatović J, Đuriš J, Cvijić S, Dobričić V, Montepietra A, Lombardi C, et al. Development of solid lipid microparticles by melt-emulsification/spray-drying processes as carriers for pulmonary drug delivery. Eur J Pharm Sci. 2021 Jan;156:105588.

256. Ingvarsson PT, Yang M, Mulvad H, Nielsen HM, Rantanen J, Foged C. Engineering of an Inhalable DDA/TDB Liposomal Adjuvant: A Quality-by-Design Approach Towards Optimization of the Spray Drying Process. Pharm Res. 2013 Nov;30(11):2772–84.

257. Ivanova Reipold E, Easterbrook P, Trianni A, Panneer N, Krakower D, Ongarello S, et al. Optimising diagnosis of viraemic hepatitis C infection: the development of a target product profile. BMC Infect Dis. 2017 Nov;17(S1):707.

258. Jaffar-Aghaei M, Khanipour F, Maghsoudi A, Sarvestani R, Mohammadian M, Maleki M, et al. QbD-guided pharmaceutical development of Pembrolizumab biosimilar candidate PSG-024 propelled to industry meeting primary requirements of comparability to Keytruda®. Eur J Pharm Sci. 2022 Jun;173:106171.

259. Jambulingam T. The R&D Marketing Interface in Biopharma and MedTech. J Commer Biotechnol [Internet]. 2019 Sep 7 [cited 2023 Nov 2];24(4). Available from: https://www.commercialbiotechnology.com/index.php/jcb/article/view/924

260. Javed MN, Kohli K, Amin S. Risk Assessment Integrated QbD Approach for Development of Optimized Bicontinuous Mucoadhesive Limicubes for Oral Delivery of Rosuvastatin. AAPS PharmSciTech. 2018 Apr;19(3):1377–91.

261. Joshi M, Yadav KS, Prabhakar B. Quality by Design Approach for Development and Optimization of Rifampicin Loaded Bovine Serum Albumin Nanoparticles and Characterization. Curr Drug Deliv. 2021 Dec 30;18(9):1338–51.

262. Kadam R, White W, Banks N, Katz Z, Dittrich S, Kelly-Cirino C. Target Product Profile for a mobile app to read rapid diagnostic tests to strengthen infectious disease surveillance. Yansouni C, editor. PLOS ONE. 2020 Jan 29;15(1):e0228311.

263. Kakade P, Gite S, Patravale V. Development of Atovaquone Nanosuspension: Quality by Design Approach. Curr Drug Deliv. 2020 Mar 12;17(2):112–25.

264. Kraan H, Van Der Stel W, Kersten G, Amorij JP. Alternative administration routes and delivery technologies for polio vaccines. Expert Rev Vaccines. 2016 Aug 2;15(8):1029–40.

265. Kuk DH, Ha ES, Ha DH, Sim WY, Lee SK, Jeong JS, et al. Development of a Resveratrol Nanosuspension Using the Antisolvent Precipitation Method without Solvent Removal, Based on a Quality by Design (QbD) Approach. Pharmaceutics. 2019 Dec 17;11(12):688.

266. Lambert WJ. Considerations in Developing a Target Product Profile for Parenteral Pharmaceutical Products. AAPS PharmSciTech. 2010 Sep;11(3):1476–81.

267. Leng D, Thanki K, Fattal E, Foged C, Yang M. Engineering of budesonide-loaded lipid-polymer hybrid nanoparticles using a quality-by-design approach. Int J Pharm. 2018 Sep;548(2):740–6.

268. Lewin SR, Attoye T, Bansbach C, Doehle B, Dubé K, Dybul M, et al. Multi-stakeholder consensus on a target product profile for an HIV cure. Lancet HIV. 2021 Jan;8(1):e42–50.

269. Lokras A, Thakur A, Wadhwa A, Thanki K, Franzyk H, Foged C. Optimizing the Intracellular Delivery of Therapeutic Anti-inflammatory TNF-α siRNA to Activated Macrophages Using Lipidoid-Polymer Hybrid Nanoparticles. Front Bioeng Biotechnol. 2021 Jan 14;8:601155.

270. Macintyre F, Ramachandruni H, Burrows JN, Holm R, Thomas A, Möhrle JJ, et al. Injectable anti-malarials revisited: discovery and development of new agents to protect against malaria. Malar J. 2018 Dec;17(1):402.

271. Malvolti S, Malhame M, Mantel CF, Le Rutte EA, Kaye PM. Human leishmaniasis vaccines: Use cases, target population and potential global demand. Laouini D, editor. PLoS Negl Trop Dis. 2021 Sep 21;15(9):e0009742.

272. Manjunatha UH, Chao AT, Leong FJ, Diagana TT. Cryptosporidiosis Drug Discovery: Opportunities and Challenges. ACS Infect Dis. 2016 Aug 12;2(8):530–7.

273. Mateus D, Marto J, Trindade P, Gonçalves H, Salgado A, Machado P, et al. Improved Morphine-Loaded Hydrogels for Wound-Related Pain Relief. Pharmaceutics. 2019 Feb 12;11(2):76.

274. Mather RG, Hopkins H, Parry CM, Dittrich S. Redefining typhoid diagnosis: what would an improved test need to look like? BMJ Glob Health. 2019 Oct;4(5):e001831.

275. Mercuri AM. Quality by Design Applied to Ocular Solid Lipid Nanoparticles Containing a Hydrophilic Peptide Prepared via Hot High Pressure Homogeniser. Curr Drug Deliv. 2016 Nov 8;13(8):1247–60.

276. Mirani AG, Patankar SP, Kadam VJ. Risk-based approach for systematic development of gastroretentive drug delivery system. Drug Deliv Transl Res. 2016 Oct;6(5):579–96.

277. Mishra SM, Rohera BD. An integrated, quality by design (QbD) approach for design, development and optimization of orally disintegrating tablet formulation of carbamazepine. Pharm Dev Technol. 2017 Oct 3;22(7):889–903.

278. Mo AX, Colley DG. Workshop report: Schistosomiasis vaccine clinical development and product characteristics. Vaccine. 2016 Feb;34(8):995–1001.

279. Nakas A, Dalatsi AM, Kapourani A, Kontogiannopoulos KN, Assimopoulou AN, Barmpalexis P. Quality Risk Management and Quality by Design for the Development of Diclofenac Sodium Intra-articular Gelatin Microspheres. AAPS PharmSciTech. 2020 May;21(4):127.

280. Namjoshi S, Dabbaghi M, Roberts MS, Grice JE, Mohammed Y. Quality by Design: Development of the Quality Target Product Profile (QTPP) for Semisolid Topical Products. Pharmaceutics. 2020 Mar 23;12(3):287.

281. Nazari K, Mehta P, Arshad MS, Ahmed S, Andriotis EG, Singh N, et al. Quality by Design Micro-Engineering Optimisation of NSAID-Loaded Electrospun Fibrous Patches. Pharmaceutics. 2019 Dec 18;12(1):2.

282. Németh Z, Pallagi E, Dobó DG, Csóka I. A Proposed Methodology for a Risk Assessment-Based Liposome Development Process. Pharmaceutics. 2020 Nov 29;12(12):1164.

283. Nsanzabana C, Ariey F, Beck HP, Ding XC, Kamau E, Krishna S, et al. Molecular assays for antimalarial drug resistance surveillance: A target product profile. Huijben S, editor. PLOS ONE. 2018 Sep 20;13(9):e0204347.

284. Oh GH, Park JH, Shin HW, Kim JE, Park YJ. Quality-by-design approach for the development of telmisartan potassium tablets. Drug Dev Ind Pharm. 2018 May 4;44(5):837–48.

285. Pallagi E, Ambrus R, Szabó-Révész P, Csóka I. Adaptation of the quality by design concept in early pharmaceutical development of an intranasal nanosized formulation. Int J Pharm. 2015 Aug;491(1–2):384–92.

286. Pallagi E, Jójárt-Laczkovich O, Németh Z, Szabó-Révész P, Csóka I. Application of the QbD-based approach in the early development of liposomes for nasal administration. Int J Pharm. 2019 May;562:11–22.

287. Patadia R, Vora C, Mittal K, Mashru RC. Quality by Design Empowered Development and Optimisation of Time-Controlled Pulsatile Release Platform Formulation Employing Compression Coating Technology. AAPS PharmSciTech. 2017 May;18(4):1213–27.

288. Patel GM, Shelat PK, Lalwani AN. QbD based development of proliposome of lopinavir for improved oral bioavailability. Eur J Pharm Sci. 2017 Oct;108:50–61.

289. Patel HP, Chaudhari PS, Gandhi PA, Desai BV, Desai DT, Dedhiya PP, et al. Nose to brain delivery of tailored clozapine nanosuspension stabilized using (+)-alpha-tocopherol polyethylene glycol 1000 succinate: Optimization and in vivo pharmacokinetic studies. Int J Pharm. 2021 May;600:120474.

290. Patel H, Patel K, Tiwari S, Pandey S, Shah S, Gohel M. Quality by Design (QbD) Approach for Development of Co-Processed Excipient Pellets (MOMLETS) By Extrusion-Spheronization Technique. Recent Pat Drug Deliv Formul. 2016 Dec 7;10(3):192–206.

291. Pellé KG, Rambaud-Althaus C, D’Acremont V, Moran G, Sampath R, Katz Z, et al. Electronic clinical decision support algorithms incorporating point-of-care diagnostic tests in low-resource settings: a target product profile. BMJ Glob Health. 2020 Feb;5(2):e002067.

292. Peraman R, Bhadraya K, Reddy Yp, Reddy Cs, Lokesh T. Analytical quality by design approach in RP-HPLC method development for the assay of etofenamate in dosage forms. Indian J Pharm Sci. 2015;77(6):751.

293. Perrier Q, Piquemal M, Leenhardt J, Choisnard L, Mazet R, Desruet MD, et al. A quality by design approach for the qualification of automating compounding device for parenteral nutrition. Eur J Pharm Sci. 2022 Dec;179:106275.

294. Porrás AI, Yadon ZE, Altcheh J, Britto C, Chaves GC, Flevaud L, et al. Target Product Profile (TPP) for Chagas Disease Point-of-Care Diagnosis and Assessment of Response to Treatment. Debrabant A, editor. PLoS Negl Trop Dis. 2015 Jun 4;9(6):e0003697.

295. Reinhard-Rupp J, Klohe K. Developing a comprehensive response for treatment of children under 6 years of age with schistosomiasis: research and development of a pediatric formulation of praziquantel. Infect Dis Poverty. 2017 Dec;6(1):122.

296. Romano J, Manning J, Hemmerling A, McGrory E, Young Holt B. Prioritizing multipurpose prevention technology development and investments using a target product profile. Antiviral Res. 2013 Dec;100:S32–8.

297. Russell C, Hussain M, Huen D, Rahman AS, Mohammed AR. Profiling gene expression dynamics underpinning conventional testing approaches to better inform pre-clinical evaluation of an age appropriate spironolactone formulation. Pharm Dev Technol. 2021 Jan 2;26(1):101–9.

298. Salami K, Gsell PS, Olayinka A, Maiga D, Formenty P, Smith PG, et al. Meeting report: WHO consultation on accelerating Lassa fever vaccine development in endemic countries, Dakar, 10–11 September 2019. Vaccine. 2020 May;38(26):4135–41.

299. Saydam M, Takka S. Development and *in vitro* evaluation of pH-independent release matrix tablet of weakly acidic drug valsartan using quality by design tools. Drug Dev Ind Pharm. 2018 Dec 2;44(12):1905–17.

300. Simões A, Veiga F, Figueiras A, Vitorino C. A practical framework for implementing Quality by Design to the development of topical drug products: Nanosystem-based dosage forms. Int J Pharm. 2018 Sep;548(1):385–99.

301. Simões A, Veiga F, Vitorino C, Figueiras A. A Tutorial for Developing a Topical Cream Formulation Based on the Quality by Design Approach. J Pharm Sci. 2018 Oct;107(10):2653–62.

302. Singh B, Kaur A, Dhiman S, Garg B, Khurana RK, Beg S. QbD-Enabled Development of Novel Stimuli-Responsive Gastroretentive Systems of Acyclovir for Improved Patient Compliance and Biopharmaceutical Performance. AAPS PharmSciTech. 2016 Apr;17(2):454–65.

303. Staunton KM, Liu J, Townsend M, Desnoyer M, Howell P, Crawford JE, et al. Designing Aedes (Diptera: Culicidae) Mosquito Traps: The Evolution of the Male Aedes Sound Trap by Iterative Evaluation. Insects. 2021 Apr 27;12(5):388.

304. Swindells S, Siccardi M, Barrett SE, Olsen DB, Grobler JA, Podany AT, et al. Long-acting formulations for the treatment of latent tuberculous infection: opportunities and challenges. Int J Tuberc Lung Dis. 2018 Feb 1;22(2):125–32.

305. Sylvester B, Tefas L, Vlase L, Tomuţă I, Porfire A. A Quality by Design (QbD) approach to the development of a gradient high-performance liquid chromatography for the simultaneous assay of curcuminoids and doxorubicin from long-circulating liposomes. J Pharm Biomed Anal. 2018 Sep;158:395–404.

306. Taipale-Kovalainen K, Karttunen AP, Ketolainen J, Korhonen O. Lubricant based determination of design space for continuously manufactured high dose paracetamol tablets. Eur J Pharm Sci. 2018 Mar;115:1–10.

307. Tanaka T, Hanaoka H, Sakurai S. Optimization of the quality by design approach for gene therapy products: A case study for adeno-associated viral vectors. Eur J Pharm Biopharm. 2020 Oct;155:88–102.

308. Thakkar R, Ashour EA, Shukla A, Wang R, Chambliss WG, Bandari S, et al. A Comparison Between Lab-Scale and Hot-Melt-Extruder-Based Anti-inflammatory Ointment Manufacturing. AAPS PharmSciTech. 2020 Jul;21(5):200.

309. Thanki K, Papai S, Lokras A, Rose F, Falkenberg E, Franzyk H, et al. Application of a Quality-By-Design Approach to Optimise Lipid-Polymer Hybrid Nanoparticles Loaded with a Splice-Correction Antisense Oligonucleotide: Maximising Loading and Intracellular Delivery. Pharm Res. 2019 Mar;36(3):37.

310. Thanki K, Zeng X, Justesen S, Tejlmann S, Falkenberg E, Van Driessche E, et al. Engineering of small interfering RNA-loaded lipidoid-poly( DL -lactic-co-glycolic acid) hybrid nanoparticles for highly efficient and safe gene silencing: A quality by design-based approach. Eur J Pharm Biopharm. 2017 Nov;120:22–33.

311. The malERA Consultative Group on Vaccines. A Research Agenda for Malaria Eradication: Vaccines. PLoS Med. 2011 Jan 25;8(1):e1000398.

312. Timpe C, Stegemann S, Barrett A, Mujumdar S. Challenges and opportunities to include patient‐centric product design in industrial medicines development to improve therapeutic goals. Br J Clin Pharmacol. 2020 Oct;86(10):2020–7.

313. Torregrosa A, Ochoa-Andrade AT, Parente ME, Vidarte A, Guarinoni G, Savio E. Development of an emulgel for the treatment of rosacea using quality by design approach. Drug Dev Ind Pharm. 2020 Feb 1;46(2):296–308.

314. Vetter B, Beran D, Boulle P, Chua A, De La Tour R, Hattingh L, et al. Development of a target product profile for a point-of-care cardiometabolic device. BMC Cardiovasc Disord. 2021 Dec;21(1):486.

315. Villamagna AH, Gore SJ, Lewis JS, Doggett JS. The Need for Antiviral Drugs for Pandemic Coronaviruses From a Global Health Perspective. Front Med. 2020 Dec 22;7:596587.

316. Vitoria M, Rangaraj A, Ford N, Doherty M. Current and future priorities for the development of optimal HIV drugs. Curr Opin HIV AIDS. 2019 Mar;14(2):143–9.

317. Vliegenthart ADB, Antoine DJ, Dear JW. Target biomarker profile for the clinical management of paracetamol overdose. Br J Clin Pharmacol. 2015 Sep;80(3):351–62.

318. Vora C, Patadia R, Mittal K, Mashru R. Risk based approach for design and optimization of stomach specific delivery of rifampicin. Int J Pharm. 2013 Oct;455(1–2):169–81.

319. Waghule T, Dabholkar N, Gorantla S, Rapalli VK, Saha RN, Singhvi G. Quality by design (QbD) in the formulation and optimization of liquid crystalline nanoparticles (LCNPs): A risk based industrial approach. Biomed Pharmacother. 2021 Sep;141:111940.

320. Walsh J, Masini T, Huttner B, Moja L, Penazzato M, Cappello B. Assessing the Appropriateness of Formulations on the WHO Model List of Essential Medicines for Children: Development of a Paediatric Quality Target Product Profile Tool. Pharmaceutics. 2022 Feb 22;14(3):473.

321. Walsh J, Schaufelberger D, Iurian S, Klein S, Batchelor H, Turner R, et al. Path towards efficient paediatric formulation development based on partnering with clinical pharmacologists and clinicians, a conect4children expert group white paper. Br J Clin Pharmacol. 2022 Dec;88(12):5034–51.

322. Wang Y, Müllertz A, Rantanen J. Structured approach for designing drug-loaded solid products by binder jetting 3D printing. Eur J Pharm Sci. 2022 Nov;178:106280.

323. Wyber R, Boyd BJ, Colquhoun S, Currie BJ, Engel M, Kado J, et al. Preliminary consultation on preferred product characteristics of benzathine penicillin G for secondary prophylaxis of rheumatic fever. Drug Deliv Transl Res. 2016 Oct;6(5):572–8.

324. Zhang E, Xie L, Qin P, Lu L, Xu Y, Gao W, et al. Quality by Design–Based Assessment for Analytical Similarity of Adalimumab Biosimilar HLX03 to Humira®. AAPS J. 2020 May;22(3):69.

325. Zidan A, Ahmed O, Aljaeid B. Nicotinamide polymeric nanoemulsified systems: a quality-by-design case study for a sustained antimicrobial activity. Int J Nanomedicine. 2016 Apr;1501.

326. Abdulgader SM, Okunola AO, Ndlangalavu G, Reeve BWP, Allwood BW, Koegelenberg CFN, et al. Diagnosing Tuberculosis: What Do New Technologies Allow Us to (Not) Do? Respiration. 2022;101(9):797–813.

327. Adepoyibi T, Lilis L, Greb H, Boyle D. Which attributes within target product profiles for tuberculosis diagnostics are the most important to focus on? Int J Tuberc Lung Dis. 2018 Apr 1;22(4):425–8.

328. Gurumukhi VC, Bari SB. Quality by design (QbD)–based fabrication of atazanavir-loaded nanostructured lipid carriers for lymph targeting: bioavailability enhancement using chylomicron flow block model and toxicity studies. Drug Deliv Transl Res. 2022 May;12(5):1230–52.

329. Monath TP, Kortekaas J, Watts DM, Christofferson RC, Desiree LaBeaud A, Gowen BB, et al. Theoretical risk of genetic reassortment should not impede development of live, attenuated Rift Valley fever (RVF) vaccines commentary on the draft WHO RVF Target Product Profile. Vaccine X. 2020 Aug;5:100060.

330. Neale G, Gaihre S, O’Gorman P, Price RK, Balzategi AG, Barrientos CH, et al. Review of recent innovations in portable child growth measurement devices for use in low- and middle-income countries. J Med Eng Technol. 2021 Nov 17;45(8):642–55.

331. Utzinger J, Becker SL, Van Lieshout L, Van Dam GJ, Knopp S. New diagnostic tools in schistosomiasis. Clin Microbiol Infect. 2015 Jun;21(6):529–42.

332. Beg S, Sandhu PS, Batra RS, Khurana RK, Singh B. QbD-based systematic development of novel optimized solid self-nanoemulsifying drug delivery systems (SNEDDS) of lovastatin with enhanced biopharmaceutical performance. Drug Deliv. 2015 Aug 18;22(6):765–84.

333. Ghaffari A, Meurant R, Ardakani A. COVID-19 Point-of-Care Diagnostics That Satisfy Global Target Product Profiles. Diagnostics. 2021 Jan 12;11(1):115.

334. Martín-Escolano J, Medina-Carmona E, Martín-Escolano R. Chagas Disease: Current View of an Ancient and Global Chemotherapy Challenge. ACS Infect Dis. 2020 Nov 13;6(11):2830–43.

335. Singh G. Target Product Profile and Clinical Development Plan. In: Pharmaceutical Medicine and Translational Clinical Research [Internet]. Elsevier; 2018 [cited 2023 Nov 2]. p. 65–80. Available from: https://linkinghub.elsevier.com/retrieve/pii/B9780128021033000055

336. Beg S, Saini S, Bandopadhyay S, Katare OP, Singh B. QbD-driven development and evaluation of nanostructured lipid carriers (NLCs) of Olmesartan medoxomil employing multivariate statistical techniques. Drug Dev Ind Pharm. 2018 Mar 4;44(3):407–20.
